# Global, regional, and national burden of 12 mental disorders in 204 countries and territories, 1990–2019: a systematic analysis for the Global Burden of Disease Study 2019

**DOI:** 10.1016/S2215-0366(21)00395-3

**Published:** 2022-02

**Authors:** GBD 2019 Mental Disorders Collaborators

## Abstract

**Background:**

The mental disorders included in the Global Burden of Diseases, Injuries, and Risk Factors Study (GBD) 2019 were depressive disorders, anxiety disorders, bipolar disorder, schizophrenia, autism spectrum disorders, conduct disorder, attention-deficit hyperactivity disorder, eating disorders, idiopathic developmental intellectual disability, and a residual category of other mental disorders. We aimed to measure the global, regional, and national prevalence, disability-adjusted life-years (DALYS), years lived with disability (YLDs), and years of life lost (YLLs) for mental disorders from 1990 to 2019.

**Methods:**

In this study, we assessed prevalence and burden estimates from GBD 2019 for 12 mental disorders, males and females, 23 age groups, 204 countries and territories, between 1990 and 2019. DALYs were estimated as the sum of YLDs and YLLs to premature mortality. We systematically reviewed PsycINFO, Embase, PubMed, and the Global Health Data Exchange to obtain data on prevalence, incidence, remission, duration, severity, and excess mortality for each mental disorder. These data informed a Bayesian meta-regression analysis to estimate prevalence by disorder, age, sex, year, and location. Prevalence was multiplied by corresponding disability weights to estimate YLDs. Cause-specific deaths were compiled from mortality surveillance databases. The Cause of Death Ensemble modelling strategy was used to estimate death rate by age, sex, year, and location. The death rates were multiplied by the years of life expected to be remaining at death based on a normative life expectancy to estimate YLLs. Deaths and YLLs could be calculated only for anorexia nervosa and bulimia nervosa, since these were the only mental disorders identified as underlying causes of death in GBD 2019.

**Findings:**

Between 1990 and 2019, the global number of DALYs due to mental disorders increased from 80·8 million (95% uncertainty interval [UI] 59·5–105·9) to 125·3 million (93·0–163·2), and the proportion of global DALYs attributed to mental disorders increased from 3·1% (95% UI 2·4–3·9) to 4·9% (3·9–6·1). Age-standardised DALY rates remained largely consistent between 1990 (1581·2 DALYs [1170·9–2061·4] per 100 000 people) and 2019 (1566·2 DALYs [1160·1–2042·8] per 100 000 people). YLDs contributed to most of the mental disorder burden, with 125·3 million YLDs (95% UI 93·0–163·2; 14·6% [12·2–16·8] of global YLDs) in 2019 attributable to mental disorders. Eating disorders accounted for 17 361·5 YLLs (95% UI 15 518·5–21 459·8). Globally, the age-standardised DALY rate for mental disorders was 1426·5 (95% UI 1056·4–1869·5) per 100 000 population among males and 1703·3 (1261·5–2237·8) per 100 000 population among females. Age-standardised DALY rates were highest in Australasia, Tropical Latin America, and high-income North America.

**Interpretation:**

GBD 2019 showed that mental disorders remained among the top ten leading causes of burden worldwide, with no evidence of global reduction in the burden since 1990. The estimated YLLs for mental disorders were extremely low and do not reflect premature mortality in individuals with mental disorders. Research to establish causal pathways between mental disorders and other fatal health outcomes is recommended so that this may be addressed within the GBD study. To reduce the burden of mental disorders, coordinated delivery of effective prevention and treatment programmes by governments and the global health community is imperative.

**Funding:**

Bill & Melinda Gates Foundation, Australian National Health and Medical Research Council, Queensland Department of Health, Australia.

## Introduction

Mental disorders are increasingly recognised as leading causes of disease burden.^1^ The *Lancet* Commission on global mental health and sustainable development^1^ emphasised mental health as a fundamental human right and essential to the development of all countries. The Commission called for more investment in mental health services as part of universal health coverage, and better integration of these services into the global response to other health priorities.^1^ To meet the men-tal health needs of individual countries in a way that prioritises transformation of health systems, in-depth understanding of the scale of the impact of these disorders is essential,^2^ including their distribution in the population, the health burden imposed, and their broader health consequences.

**Research in context**

**Evidence before this study**

The last comprehensive review of the global burden of mental disorders was published on Nov 9, 2013 using the Global Burden of Diseases, Injuries, and Risk Factors Study (GBD) 2010 findings, and in subsequent years there have been important updates to the burden estimation methodology and epidemiological datasets. GBD 2019 estimated the prevalence and burden due to 12 mental disorders by age, sex, year, and location. High-level findings of GBD 2019 were published in a capstone publication by Vos and colleagues in 2020, which covered all diseases and injuries. We searched PubMed, PsycINFO, Embase, and PROSPERO for papers on the global burden of mental disorders published between Oct 17, 2020 and Oct 6, 2021. We used the following search terms: (((“Mental disorders”[Title/Abstract]) AND (Global[Title/Abstract],)) AND (2019[Title/Abstract])) AND ((((“GBD 2019”[Title/Abstract]) OR (Disability[Title/Abstract])) OR (Prevalence[Title/Abstract])) OR (Burden[Title/Abstract])). For the PROSPERO search the following additional filters were applied: Health area of review: mental health and behavioural conditions; Type and method of review: epidemiologic, systematic review, meta-analysis, and review of reviews. Our search yielded 102 studies, of which 12 were relevant to our research aim. Of these 12 studies, two reported GBD 2019 results for eating disorders in China, and mental disorders in Mexico. We found no publications dedicated to findings of GBD 2019 mental disorders globally or for any other location by age, sex, and year.

**Added value of this study**

Using the most up-to-date information on the prevalence and burden of mental disorders across the global population, excluding substance use disorders and suicide, for 2019, we observed similar disparities in the burden of mental disorders as in 1990. Mental disorders remained among the leading causes of burden globally. Disability-adjusted life-years (DALYs) for mental disorders were evident across all age groups, emerging before age 5 years in individuals with idiopathic intellectual disability and autism spectrum disorders, and continued to be evident at older ages in people with depressive disorders, anxiety disorders, and schizophrenia. We identified priority areas for improvement of the epidemiological data and burden estimation methodology for mental disorders, and provided recommendations as to how these areas might be addressed.

**Implications of all the available evidence**

GBD 2019 confirmed that a large proportion of the world's disease burden is attributable to mental disorders and found no evidence of a global reduction in that burden since 1990, despite research demonstrating that interventions can achieve a reduction in the burden. Our findings highlighted the limitations of measures for estimating years of life lost for determining the effects of mental disorders on premature mortality. Research is needed to improve these measures to provide a more accurate picture of the true burden due to mental disorders.

The Global Burden of Diseases, Injuries, and Risk Factors Study (GBD) 2019 is a comprehensive international effort that includes the measurement of the burden of mental disorders. GBD 2019 used the disability-adjusted life-year (DALY) metric, which measures the gap between the current health of the population and a normative standard life expectancy spent in full health. GBD 2019 built on previous iterations of the GBD study by incorporating new data and methodological improvements. The study enabled systematic comparison of the prevalence and burden due to 369 diseases and injuries.^3^, ^4^, ^5^ Between 1990 and 2019, a reduction in DALYs from communicable, maternal, neonatal, and nutritional diseases has been offset by an increase in burden due to non-communicable diseases, including mental disorders.^3^, ^4^, ^5^

The last comprehensive review of the global burden of mental disorders was published on the basis of GBD 2010 findings, in which the combined burden of mental and substance use disorders was presented.^6^ Mental and substance use disorders are a heterogeneous group of disorders. Health systems in many countries organise their services for these disorder groups separately, whereas in resource-poor settings, these disorders are often grouped within essential health care packages and delivery platforms. In this study we focused on mental disorders, which allowed us to present a more detailed analysis of their distribution and burden by age, sex, location, and year than that provided by previous publications.^5^, ^6^ This supplements GBD 2016 findings for substance use disorders published separately.^7^ There have also been significant updates to the burden estimation methodology and epidemiological datasets underpinning GBD findings since this publication.^6^

In this study, we aimed to facilitate access and interpretation of GBD 2019 estimates for stakeholders, including governments and international agencies, researchers, and clinicians involved in the identification, management, and prevention of mental disorders; present and evaluate the methods used to estimate the burden of mental disorders; and highlight priority areas for improvement in the mental disorder burden estimation methodology.

## Methods

### Overview

GBD 2019 estimated incidence, prevalence, mortality, years lived with disability (YLDs), years of life lost (YLLs), and DALYs for 369 diseases and injuries, for males and females, 23 age groups, 21 regions, 204 countries and territories, from 1990 onwards.

Our analysis was done as part of the GBD Collaborator Network and in accordance with the GBD protocol. All GBD 2019 analyses adhered to the Guidelines for Accurate and Transparent Health Estimates Reporting (appendix p 2).^8^ Comprehensive explanations of burden estimation methods have been published elsewhere.^3^, ^4^, ^5^ Here, we summarise the methodology for estimating the health burden due to mental disorders.

### Case definitions

The 369 diseases and injuries included in GBD 2019 are organised into a four-level cause hierarchy. Causes within each level are mutually exclusive and collectively exhaustive. The four levels within the cause hierarchy and the position of each mental disorder within the hierarchy are presented in the appendix (p 5). The mental disorders included in GBD 2019 were depressive disorders (major depressive disorder and dysthymia), anxiety disorders (a combined estimate of all subtypes), bipolar disorder (a combined estimate of all subtypes), schizophrenia, autism spectrum disorders, conduct disorder, attention-deficit hyperactivity disorder (ADHD), eating disorders (anorexia nervosa and bulimia nervosa), idiopathic developmental intellectual disability (estimated as part of the broader intellectual disability impairment envelope in GBD 2019, comprising intellectual disability from any unknown source after all other sources of intellectual disability are accounted for), and a residual category of other mental disorders (an aggregate group of personality disorders). To allow for comparability in measurement, case definitions predominantly adhered to DSM-IV-TR or ICD-10) criteria, since these were used by the majority of included mental health surveys. The mental disorders included in GBD 2019 and their definitions are further explained in the appendix (p 4).

### Estimation of YLDs

YLDs were estimated by multiplying prevalence estimates at varying levels of severity by an appropriate disability weight. Disability weights quantified the amount of health loss associated with each sequela (or consequence of a disease or injury).^5^ A flowchart presenting the methodology for estimating YLDs is shown in the appendix (p 6).

#### Data sources

To compile the epidemiological datasets required to estimate YLDs for each disorder, we did a systematic literature review of PsycINFO, Embase, and PubMed supplemented with searches of the grey literature, and expert consultation. No language restrictions were used. The keywords used in our search of electronic databases are shown in the appendix (p 7). We also reviewed data sources archived in the Global Health Data Exchange, major multinational survey data catalogues, and data sources recommended by GBD collaborators who reviewed the results of our search for each disorder. Included data sources were surveys reporting estimates of mental disorder prevalence, incidence, remission, or excess mortality. We included surveys published from Jan 1, 1980 that used probability sampling to capture a representative sample of the general population. Selection bias and non-response were considered as part of the assessment for eligibility for inclusion and weighted estimates were prioritised during data extraction. Surveys with recruitment strategies producing samples with a different risk profile for mental disorders compared with the general population, such as surveys that used non-probabilistic sampling or reported on population subgroups were excluded. Treatment samples were considered only if the source was likely to capture all cases of the disorder in the population—eg, for autism spectrum disorders with a bias correction. Studies utilising different versions of DSM and ICD were included. To estimate prevalence, we included estimates reporting past-year prevalence or less for all disorders. Owing to the risk of recall bias for many disorders in measures of lifetime prevalence, this measure was included only for bipolar disorder and autism spectrum disorders using prospective design.^9^

#### Epidemiological disease models

The epidemiological data obtained from our systematic reviews were analysed in two steps. For step 1, we tested and adjusted for biases in epidemiological estimates reported between studies. For step 2, “gold-standard” (ie, estimates using the desired data-collection methodology and not requiring bias adjustments) and adjusted estimates were modelled within a meta-regression analysis.

For each disorder, we identified the major sources of bias in the extracted data, on the basis of known sources of measurement error such as recall type (point, 12-month, or lifetime prevalence), survey instrument (diagnostic or symptom scale), and survey interviewer (lay or clinician). Estimates with these biases were considered alternative estimates to gold-standard estimates and were adjusted. The adjustment factor was the pooled ratio between gold-standard estimates and these alternative estimates. We compiled studies reporting both the gold-standard estimate and the alternative estimate (eg, point prevalence and 12-month prevalence) and calculated the ratios within these studies. We also looked for pairs of gold-standard and alternative estimates between studies, matched by age (0—99 years), sex, location (across 82 locations), and year (1980 onwards), and calculated the ratio between these estimates.

Prevalence ratios between alternative estimates were used together with prevalence ratios between gold-standard and alternative estimates and analysed within a network. Prevalence ratios between alternative estimates were especially useful for alternative estimate types with few gold-standard to alternative ratios available. Direct versus indirect effects were inspected for transitivity and indirect estimates excluded when these effects were markedly different. We did network meta-analyses of these ratios using meta-regression—Bayesian, regularised, trimmed to calculate pooled ratios between gold-standard estimates and alternative estimates.^10^ These pooled ratios were used as an adjustment factor to correct alternative estimates before analysis. More detailed information about the bias correction process is presented elsewhere.^5^

The gold-standard and adjusted estimates were modelled using DisMod-MR 2.1, a Bayesian meta-regression tool that pools data from different sources to produce internally consistent estimates of prevalence, incidence, remission, and excess mortality by age, sex, location, and year.^11^ As part of this process, estimates were generated for locations where high-quality raw epidemiological data were unavailable by using the modelled output from surrounding locations.^11^ As per the GBD protocol, an uncertain estimate is preferable to no estimate when data are sparse or not available, because no estimate would result in no health loss from that condition being recorded. DisMod-MR 2.1 also uses location-level covariates to predict prevalence by location. We included location-level covariates for major depressive disorder and anxiety disorders. The first covariate identified the mean mortality rate in the previous ten years due to war and terrorism for each GBD location, considering the known association between conflict and elevated prevalence of major depressive disorder and anxiety disorders.^12^ The second covariate used the Gallup Negative Experience Index, which measures past-day experiences of physical pain, worry, sadness, stress, and anger from population surveys conducted within the Gallup Initiative.^13^ The covariate was included as a method to test for an association between negative emotions at a location level and major depressive disorder and anxiety disorder prevalence. The third covariate used the proportion of major depressive disorder burden caused by two risk factors, intimate partner violence and childhood sexual abuse, to inform the estimation of prevalence. The choice of scale for location-level covariates differed by disorder and covariate. Both the untransformed and log-transformed covariates were tested as part of the modelling process for each disorder. The final decision for scale was determined based on the coefficient, statistical significance, and skew of the location-level covariate. The priors used to inform the DisMod-MR 2.1 models for each disorder are summarised in the appendix (p 8). More information on DisMod-MR 2.1, covariates, and priors is presented elsewhere.^5^, ^11^

#### Severity proportions

Severity proportions were calculated to reflect the varying levels of disability (or sequelae) associated with a specific disorder—eg, mild, moderate, and severe presentations. For conduct disorder, ADHD, autism spectrum disorders, bipolar disorder, and schizophrenia, severity distributions were obtained from meta-analyses of survey data.^14^, ^15^ For depressive disorders, anxiety disorders, and other mental disorders, we used individual-level survey data from the US National Epidemiologic Survey on Alcohol and Related Conditions^16^ or the 1997 Australian National Survey of Mental Health and Wellbeing.^5^, ^17^, ^18^ No severity distribution was estimated for eating disorders. Severity proportions were applied to the total prevalent cases estimated by DisMod-MR 2.1 to obtain prevalence estimates for each level of severity (appendix p 9). Further details on severity proportions have been presented elsewhere.^5^, ^18^

#### Disability weights

Severity-specific prevalence estimates were multiplied by a corresponding disability weight to estimate YLDs.^5^ We used disability weights derived from community-based surveys in Bangladesh, Indonesia, Peru, Tanzania, the USA, Hungary, Italy, Sweden, and the Netherlands, and an open web-based survey available in English, Spanish, and Mandarin. In these surveys, participants were presented with pairs of health state descriptions and asked to select the “healthier” state. Responses were anchored on a scale ranging from 0 (perfect health) to 1 (death) using additional population health equivalence questions that compared the benefits of lifesaving and disease-prevention programmes for several health states. The analysis of pair-wise comparisons indicated the relative position of health states to each other, and the population health equivalence questions were required to anchor those relative positions as values on a 0 to 1 scale. Sequela-specific health state descriptions and disability weights have been summarised in the appendix (p 9). More information on the disability weights analysis has been presented elsewhere.^5^

#### Comorbidity adjustments

Since burden attributable to each GBD cause was estimated separately, a simulation method was used to adjust for comorbidity. The co-occurrence of different diseases and injuries was estimated by simulating populations of 40 000 individuals by location, age, sex, and year. Simulated individuals within each population were exposed to the independent probability of having any combination of sequelae in GBD 2019. The comorbidity correction estimated the difference between the average disability weight of individuals experiencing one sequela and the multiplicatively combined disability weight of those experiencing multiple sequelae. The average comorbidity correction estimated for each sequela was applied to the respective location-specific, age-specific, sex-specific, and year-specific YLDs. Further information about comorbidity correction used in GBD has been presented elsewhere.^5^

### Estimation of YLLs

YLLs were calculated by multiplying cause-specific deaths by the years of life expected to be remaining at death based on a normative life expectancy.^5^ The GBD 2019 cause of death database contained vital registration, verbal autopsy, cancer registry, police records, sibling history, surveillance, and survey or census data collected since 1980. The Cause of Death Ensemble modelling strategy was used to model cause of death data by location, age, sex, and year. Deaths were scaled to total mortality. Normative life tables were generated using data on the lowest observed death rates for any age group within all GBD locations with a total population greater than 5 million.^3^

Each death in GBD could be allocated to only one underlying cause as per ICD categorisation of causes of death. Deaths and YLLs could be calculated only for anorexia nervosa and bulimia nervosa, since these were the only mental disorders identified as underlying causes of death. Deaths and YLL estimates are not reflective of all premature mortality in individuals with mental disorders where the direct cause of death is another disease or injury. For example, suicide was categorised separately under injuries and not included within the mental disorders group. A method for capturing the proportion of premature deaths from other diseases or injuries which can be causally attributed to the mental disorder experienced by a person is not yet available for our estimation of YLLs.

### Estimation of DALYs

Overall, we included 29 incidence, 1075 prevalence, 52 remission, 1930 cause of death, and 149 severity or other types of data sources in the estimation of YLDs, YLLs, and DALYs for mental disorders. The number of data sources available by disorder and parameter is summarised in the appendix (p 11). Further information on data sources has also been presented elsewhere.^19^, ^20^, ^21^, ^22^, ^23^, ^24^, ^25^

DALYs were calculated as the sum of YLDs and YLLs. For mental disorders not recognised as causes of death, YLLs were not estimated and YLDs approximated DALYs. Age-standardised rates per 100 000 people were estimated using the GBD world population age standard. Change in prevalence and burden across time was estimated by comparing the change in age-standardised rate and the change in total numbers. The GBD 2019 geographical hierarchy included 204 countries and territories aggregated into 21 regions and seven super-regions. YLDs, YLLs, and DALYs were estimated at all levels of this geographical hierarchy, by sex, for 23 age groups (age 0 to 95 years and older), and for every year from 1990 to 2019. We estimated 95% uncertainty intervals (UIs) for all estimates derived from the 25th and 975th ordinals of 1000 draws of the posterior distribution at each step of the burden estimation process. Microsoft Excel or the maptools package in R (version 3.6.3) were used to generate all tables and figures.

### Role of the funding source

The funders of this study had no role in study design, data collection, data analysis, data interpretation, or the writing of the report.

## Results

Here, we present the main GBD findings for mental disorders. All GBD 2019 outputs by age, sex, year, location are available in a set of interactive online visualisations.^25^

Mental disorders accounted for 654·8 million estimated cases (95% UI 603·6–708·1) in 1990 and 970·1 million cases (900·9–1044·4) in 2019, corresponding to an increase of 48·1% between 1990 and 2019 (table 1). No marked increases were identified in the age-standardised prevalence of any mental disorder between 1990 and 2019.

**Table 1:** Global prevalence and age-standardised prevalence for mental disorders in 1990 and 2019

	**1990**		**2019**	
	Prevalence, in millions (95% UI)	Age-standardised prevalence per 100 000 people (95% UI)	Prevalence, in millions (95% UI)	Age-standardised prevalence per 100 000 people (95% UI)*
**Mental disorders**				
Total	654·8 (603·6–708·1)	12 579·3 (11 634·4–13 552·2)	970·1 (900·9–1044·4)	12 262·0 (11 382·9–13 213·3)
Male	317·8 (290·8–346·7)	12 020·0 (11 061·2–13 042·4)	462·2 (427·5–499·7)	11 727·3 (10 835·7–12 693·9)
Female	337·0 (310·1–363·8)	13 100·4 (12 114·8–14 090·9)	507·9 (471·2–547·4)	12 760·0 (11 831·7–13 763·1)
**Anxiety disorders**				
Total	194·9 (165·1–231·2)	3791·6 (3194·0–4476·6)	301·4 (252·6–356·0)	3779·5 (3181·1–4473·3)
Male	73·4 (61·3–87·0)	2839·2 (2388·7–3332·9)	113·9 (95·4–135·1)	2859·8 (2397·0–3379·9)
Female	121·5 (102·0–144·7)	4732·2 (3983·0–5605·5)	187·5 (157·7–221·6)	4694·7 (3945·6–5576·9)
**Depressive disorders**				
Total	170·8 (152·7–190·4)	3486·2 (3140·8–3855·7)	279·6 (251·6–310·3)	3440·1 (3097·0–3817·6)
Male	65·6 (58·5–73·2)	2700·7 (2432·1–2987·4)	109·2 (98·0–121·4)	2713·3 (2438·3–3013·1)
Female	105·2 (94·3–117·3)	4262·5 (3844·6–4730·0)	170·4 (153·6–188·7)	4158·4 (3746·9–4616·3)
**Other mental disorders**				
Total	67·7 (52·7–86·5)	1434·7 (1116·4–1822·6)	117·2 (90·8–148·7)	1428·7 (1108·4–1816·1)
Male	39·9 (30·8–51·0)	1702·3 (1323·7–2155·4)	68·3 (53·0–86·6)	1690·1 (1311·0–2138·8)
Female	27·8 (21·4–35·4)	1173·9 (909·9–1485·8)	48·9 (37·8–61·8)	1173·1 (905·6–1484·9)
**Idiopathic developmental intellectual disability**				
Total	92·8 (58·3–128·6)	1641·9 (1028·1–2278·2)	107·6 (65·8–150·4)	1426·6 (873·6–1991·7)
Male	47·7 (29·4–66·7)	1657·2 (1017·0–2325·9)	54·9 (32·8–77·6)	1436·4 (860·4–2027·8)
Female	45·2 (29·2–61·6)	1625·3 (1048·2–2220·8)	52·7 (33·1–72·8)	1415·4 (891·3–1954·5)
**Attention-deficit hyperactivity disorder**				
Total	72·4 (52·9–96·4)	1240·5 (909·6–1647·1)	84·7 (62·5–111·3)	1131·9 (831·7–1494·5)
Male	52·6 (38·6–70·7)	1768·3 (1304·2–2350·6)	61·5 (45·4–80·9)	1611·6 (1184·8–2134·1)
Female	19·8 (14·2–26·4)	693·4 (497·9–918·5)	23·2 (16·8–31·0)	631·0 (455·7–846·5)
**Conduct disorder**				
Total	32·7 (23·6–42·5)	537·9 (388·2–699·0)	40·1 (29·0–52·0)	559·0 (405·0–722·3)
Male	21·6 (16·1–27·7)	694·7 (517·7–891·4)	26·3 (19·6–33·4)	711·2 (530·5–904·0)
Female	11·1 (7·4–15·3)	374·0 (248·7–515·5)	13·8 (9·1–19·0)	397·3 (263·8–545·5)
**Bipolar disorder**				
Total	24·8 (20·6–29·4)	490·1 (411·0–576·5)	39·5 (33·0–46·8)	489·8 (407·5–580·6)
Male	11·6 (9·6–13·8)	459·4 (384·9–540·6)	18·8 (15·7–22·3)	466·9 (388·5–552·9)
Female	13·2 (10·9–15·5)	520·9 (435·1–613·3)	20·7 (17·3–24·6)	512·8 (425·6–609·0)
**Autism spectrum disorders**				
Total	20·3 (16·9–24·2)	372·8 (309·1–444·9)	28·3 (23·5–33·8)	369·4 (305·9–441·2)
Male	15·6 (13·0–18·6)	571·2 (473·8–679·6)	21·6 (18·0–25·8)	560·1 (465·2–667·3)
Female	4·7 (3·8–5·7)	173·4 (140·9–211·5)	6·7 (5·4–8·2)	176·3 (143·0–214·5)
**Schizophrenia**				
Total	14·2 (12·2–16·5)	289·9 (249·8–333·2)	23·6 (20·2–27·2)	287·4 (246·2–330·9)
Male	7·5 (6·4–8·7)	304·5 (262·6–350·0)	12·4 (10·6–14·3)	302·7 (259·7–348·4)
Female	6·7 (5·8–7·7)	274·9 (236·9–315·5)	11·2 (9·6–12·9)	272·0 (232·7–313·7)
**Eating disorders**				
Total	8·5 (6·4–10·9)	150·5 (113·1–192·1)	13·6 (10·2–17·5)	174·0 (130·1–222·1)
Male	2·8 (2·0–3·7)	96·7 (69·1–128·0)	4·7 (3·3–6·2)	117·9 (84·6–156·1)
Female	5·7 (4·3–7·2)	205·8 (156·2–258·6)	9·0 (6·8–11·3)	231·5 (175·1–291·4)

95% UI=95% uncertainty interval.

* Disorders ordered from highest to lowest on the basis of total age-standardised rates in 2019.

The age-standardised prevalence for the aggregate of mental disorders was largely consistent for males and females in 2019 (11 727·3 cases per 100 000 people [95% UI 10 835·7–12 693·9] in males *vs* 12 760·0 cases per 100 000 people [11 831·7–13 763·1] in females). Depressive disorders, anxiety disorders, and eating disorders were more common in females than males. ADHD and autism spectrum disorders were more common in males. Across both sex and year, the two most common mental disorders were depressive disorders and anxiety disorders. The least common were schizophrenia and eating disorders (table 1).

Table 2 presents age-standardised prevalence by mental disorder and region in 2019. For the aggregate of mental disorders, Australasia, Tropical Latin America, and high-income North America had the highest prevalence. Across individual disorders, other regional patterns emerged. The prevalence of depressive disorders was high in sub-Saharan Africa (4540·4 cases per 100 000 people [95% UI 4038·1–5112·4]) and north Africa and the Middle East (4348·9 cases per 100 000 people [3807·3–4971·1]) in addition to Australasia, Tropical Latin America, and high-income North America (table 2). The age-standardised prevalence of eating disorders, ADHD, conduct disorder, and autism spectrum disorders was highest in high-income regions. Bipolar disorder and schizophrenia prevalence varied to a lesser extent across regions. Disorder-specific prevalence by country is presented in the appendix (pp 12, 24).

**Table 2:** Age-standardised prevalence per 100 000 people by mental disorder and region, 2019

		**Schizophrenia**	**Depressive disorders**	**Anxiety disorders**	**Bipolar disorder**	**Eating disorders**	**Autism spectrum disorders**	**Attention-deficit hyperactivity disorder**	**Conduct disorder**	**Idiopathic developmental intellectual disability**	**Other mental disorders**
**Central Europe, eastern Europe, and central Asia**	**10 517·2 (9743·6–11 353·4)**	**282·1 (236·0–331·1)**	**3081·4 (2747·1–3442·3)**	**2993·3 (2501·3–3562·5)**	**526·7 (430·6–630·9)**	**150·2 (111·1–193·9)**	**385·5 (317·8–462·4)**	**1072·8 (764·2–1453·7)**	**604·7 (440·8–780·4)**	**606·4 (286·3–930·5)**	**1401·5 (1076·5–1783·5)**
Central Asia	10 129·5 (9344·1–11 003·9)	274·7 (220·3–333·4)	3186·5 (2807·9–3644·1)	2221·6 (1751·5–2773·5)	513·6 (401·5–647·5)	126·5 (93·1–163·6)	374·8 (308·0–450·9)	1059·1 (758·1–1421·8)	584·8 (420·8–764·5)	861·5 (475·4–1258·4)	1454·7 (1127·4–1861·2)
Central Europe	10 254·7 (9459·9–11 144·7)	292·0 (241·1–345·2)	2601·0 (2309·7–2956·2)	3276·1 (2685·6–3986·5)	556·7 (449·1–675·6)	173·0 (127·0–222·8)	373·6 (308·4–446·9)	1072·0 (764·8–1442·1)	598·1 (435·7–774·8)	460·0 (188·1–732·9)	1427·2 (1097·6–1821·7)
Eastern Europe	10 828·1 (10 048·4–11 656·1)	279·3 (238·2–323·2)	3316·4 (2964·2–3683·9)	3188·5 (2727·1–3719·6)	516·2 (434·7–603·7)	151·1 (112·4–195·3)	397·3 (328·3–476·0)	1084·2 (774·0–1496·1)	621·8 (458·7–802·8)	533·9 (225·4–844·9)	1358·5 (1038·2–1723·1)
**High income**	**14 054·7 (13 090·5–15 168·3)**	**333·0 (286·4–382·8)**	**3659·9 (3307·4–4062·6)**	**5058·3 (4242·7–6047·4)**	**773·5 (660·3–887·4)**	**444·7 (340·2–554·1)**	**599·7 (502·2–709·4)**	**1693·1 (1235·0–2269·9)**	**588·9 (429·5–763·0)**	**404·1 (136·6–690·0)**	**1642·0 (1277·1–2080·5)**
Australasia	17 506·7 (16 261·0–19 026·6)	388·5 (357·3–422·1)	4284·3 (3764·6–4908·9)	6031·9 (4885·4–7447·5)	1182·1 (993·7–1373·2)	969·2 (796·6–1149·7)	436·1 (363·8–521·2)	3248·8 (2476·1–4108·9)	617·0 (484·1–785·3)	318·4 (100·3–548·7)	1858·8 (1535·2–2216·7)
High-income Asia Pacific	9796·9 (9077·4–10 606·6)	301·5 (253·8–352·5)	2084·3 (1885·6–2313·1)	2616·4 (2184·4–3108·2)	601·0 (496·6–706·0)	379·2 (288·7–481·0)	634·3 (528·8–756·7)	1453·2 (1052·4–1958·7)	558·6 (405·5–730·5)	180·2 (27·2–357·2)	1516·2 (1172·5–1933·5)
High-income North America	15 445·8 (14 406·4–16 693·3)	418·9 (363·8–479·2)	4270·3 (3867·9–4743·3)	5559·9 (4693·5–6582·6)	621·2 (579·5–663·6)	424·7 (316·0–540·5)	640·0 (537·7–756·4)	2096·8 (1505·1–2838·7)	549·4 (386·7–720·7)	435·0 (136·7–745·1)	1792·5 (1372·9–2247·9)
Southern Latin America	13 056·6 (12 197·9–14 002·5)	313·4 (251·9–380·9)	2777·3 (2492·5–3111·5)	5125·8 (4459·8–5885·1)	1024·5 (794·6–1273·0)	340·4 (253·8–434·8)	482·5 (400·8–579·0)	1289·0 (934·1–1738·4)	573·0 (416·6–741·2)	524·2 (198·9–847·0)	1590·1 (1226·8–2047·4)
Western Europe	14 528·7 (13 440·8–15 749·2)	272·6 (229·9–318·0)	3851·3 (3448·1–4296·6)	5626·6 (4632·7–6814·1)	901·8 (735·7–1069·3)	470·3 (363·3–586·6)	581·3 (488·2–686·4)	1363·5 (992·1–1824·1)	639·6 (468·4–822·6)	448·8 (168·7–746·0)	1556·7 (1202·2–1993·6)
Latin America and Caribbean*	13 804·2 (12 793·5–14 941·6)	277·8 (234·0–325·5)	3417·1 (3079·4–3791·4)	5502·3 (4625·9–6588·7)	963·7 (794·2–1138·9)	231·4 (170·9–298·0)	350·4 (288·8–419·7)	1813·3 (1327·6–2443·9)	573·8 (416·0–745·6)	381·2 (144·8–626·3)	1398·2 (1072·1–1777·0)
Andean Latin America	13 562·0 (12 372·6–15 010·6)	276·2 (221·3–334·9)	2725·6 (2380·0–3105·2)	5497·3 (4467·8–6893·1)	910·5 (700·6–1142·2)	281·6 (201·2–378·0)	342·1 (282·4–410·5)	2116·8 (1537·0–2831·8)	571·8 (410·8–742·3)	419·5 (166·0–669·4)	1461·4 (1132·7–1868·4)
Central Latin America	11 806·9 (10 931·6–12 775·4)	279·6 (234·1–328·8)	3198·5 (2865·7–3562·3)	3930·7 (3253·4–4782·6)	854·0 (703·0–1015·8)	224·9 (165·7–292·3)	350·9 (288·8–419·5)	1403·7 (1033·9–1903·0)	575·9 (421·2–746·1)	351·4 (125·8–584·8)	1405·1 (1078·9–1791·6)
Tropical Latin America	15 909·4 (14 825·7–17 139·0)	277·7 (237·7–320·2)	3799·4 (3464·3–4168·9)	7378·6 (6296·1–8605·9)	1111·1 (933·7–1288·1)	231·9 (173·2–296·5)	353·9 (292·0–425·3)	1945·0 (1418·3–2672·7)	574·9 (414·7–751·3)	357·2 (126·1–596·2)	1360·5 (1039·5–1723·8)
Caribbean	14 362·4 (13 110·6–15 834·0)	271·4 (218·7–329·3)	3673·6 (3212·5–4178·7)	4400·7 (3522·5–5499·8)	908·2 (695·0–1141·6)	193·9 (141·5–252·4)	343·8 (283·7–413·6)	3064·4 (2247·0–4115·1)	559·3 (405·6–723·1)	602·9 (284·1–929·8)	1459·5 (1131·2–1866·5)
North Africa and Middle East*	14 937·8 (13 736·6–16 219·8)	248·2 (203·9–294·9)	4348·9 (3807·3–4971·1)	5135·7 (4164·9–6267·2)	758·8 (595·7–939·1)	216·9 (159·7–280·2)	304·4 (251·2–366·1)	1245·1 (909·8–1667·4)	591·9 (433·4–762·5)	1850·5 (1157·7–2571·2)	1462·8 (1128·4–1867·2)
**South Asia**	**13 106·0 (11 801·3–14 243·3)**	**283·5 (242·5–328·7)**	**3794·7 (3416·0–4199·7)**	**3045·5 (2594·5–3547·2)**	**361·4 (303·7–423·5)**	**126·7 (92·9–163·9)**	**290·0 (238·4–349·2)**	**609·4 (431·3–832·3)**	**538·2 (383·9–711·9)**	**3555·1 (2434·9–4716·8)**	**1378·6 (1054·5–1748·1)**
**Southeast Asia, east Asia, and Oceania**	**10 520·4 (9722·0 −11 377·3)**	**305·9 (265·8–349·2)**	**2723·9 (2451·5–3022·4)**	**3292·9 (2801·9–3821·7)**	**226·9 (189·5–267·8)**	**111·2 (82·1–143·7)**	**348·1 (287·9–417·2)**	**1622·4 (1212·9–2135·6)**	**511·4 (367·3–666·5)**	**577·5 (288·8–875·4)**	**1383·7 (1059·1–1752·9)**
East Asia	10 566·7 (9749·8–11 424·3)	309·2 (272·8–348·0)	2720·1 (2449·9–3004·9)	3180·7 (2712·3–3663·7)	182·0 (153·6–211·1)	112·7 (83·6–145·3)	367·8 (304·4–441·9)	2038·0 (1531·9–2662·2)	465·0 (326·9–609·6)	399·1 (163·6–639·3)	1371·0 (1048·4–1737·9)
Southeast Asia	10 551·5 (9724·2–11 420·5)	298·5 (249·6–353·1)	2610·6 (2302·9–2958·4)	3633·2 (3024·1–4315·0)	331·4 (272·5–399·6)	109·6 (81·4–141·3)	312·5 (257·7–374·4)	1000·5 (723·4–1365·7)	571·7 (417·2–745·2)	886·1 (491·8–1289·8)	1405·6 (1080·0–1791·7)
Oceania	11 599·7 (10 568·4–12 793·7)	273·9 (220·9–333·9)	3044·8 (2622·9–3541·7)	4006·8 (3182·9–4990·4)	265·1 (206·8–333·3)	84·5 (61·2–109·3)	289·0 (235·5–349·0)	1131·3 (802·6–1567·5)	535·1 (374·8–698·5)	1213·3 (745·5–1695·1)	1471·1 (1139·9–1879·3)
**Sub-Saharan Africa**	**11 934·6 (11 080·2–12 879·4)**	**214·2 (178·2–254·3)**	**4540·4 (4038·1–5112·4)**	**3462·6 (2839·1–4184·2)**	**566·4 (458·1–690·1)**	**106·7 (78·3–137·7)**	**373·5 (307·4–447·6)**	**583·8 (414·2–797·0)**	**592·7 (430·2–763·1)**	**806·1 (398·8–1237·4)**	**1415·7 (1088·2–1808·5)**
Central sub-Saharan Africa	13 396·2 (12 307·9–14 613·9)	208·5 (166·2–253·9)	5536·9 (4801·3–6307·6)	3864·0 (3089·6–4826·5)	554·3 (432·0–696·3)	93·7 (68·8–120·7)	370·8 (303·3–446·9)	569·6 (403·3–776·8)	588·6 (432·7–757·8)	1052·6 (572·8–1570·3)	1456·9 (1129·1–1864·0)
Eastern sub-Saharan Africa	12 616·5 (11 687·3–13 609·8)	210·8 (174·3–250·2)	4849·2 (4317·2–5416·8)	3716·3 (3050·0–4530·6)	595·6 (480·3–722·6)	92·6 (68·1–119·6)	378·4 (311·7–454·4)	572·4 (404·0–779·4)	597·0 (436·2–766·8)	997·0 (537·0–1504·4)	1419·2 (1091·7–1813·0)
Southern sub-Saharan Africa	11 453·9 (10 687·1–12 279·3)	220·9 (187·5–256·8)	4166·3 (3736·3–4612·3)	3658·0 (3100·4–4307·8)	553·2 (459·0–654·1)	151·2 (111·9–196·6)	371·6 (304·9–447·7)	575·3 (404·0–789·5)	617·9 (456·6–801·4)	443·4 (176·1–722·3)	1379·9 (1057·1–1747·4)
Western sub-Saharan Africa	11 000·7 (10 217·1–11 866·6)	217·1 (181·1–256·5)	4075·4 (3633·0–4556·1)	3066·5 (2532·6–3683·3)	546·6 (445·2–661·4)	114·4 (84·0–148·0)	370·6 (305·5–443·3)	599·6 (421·8–832·2)	586·7 (423·0–763·5)	626·0 (282·1–1001·2)	1408·6 (1081·2–1797·9)
**Global**	**12 262·0 (11 382·9–13 213·3)**	**287·4 (246·2–330·9)**	**3440·1 (3097·0–3817·6)**	**3779·5 (3181·1–4473·3)**	**489·8 (407·5–580·6)**	**174·0 (130·1–222·1)**	**369·4 (305·9–441·2)**	**1131·9 (831·7–1494·5)**	**559·0 (405·0–722·3)**	**1426·6 (873·6–1991·7)**	**1428·7 (08·4–1816·1)**

95% uncertainty intervals are shown in parentheses. Bolding indicates global estimates or GBD super-regions. GBD=Global Burden of Diseases, Injuries, and Risk Factors Study.

Eating disorders were the cause of 318·3 deaths (95% UI 285·7–386·0) worldwide in 2019. Anorexia nervosa accounted for most of these deaths (268·7 deaths [242·5–326·9]). The remaining deaths (49·6 [36·4–72·2]) were due to bulimia nervosa. Eating disorders were the only mental disorders for which YLLs could be estimated.

Mental disorders accounted for 125·3 million DALYs (95% UI 93·0–163·2) in 2019, equating to an age-standardised DALY rate of 1566·2 (1160·1–2042·8) per 100 000 people, or 4·9% (3·9–6·1) of global DALYs. The number and proportion of DALYs due to mental disorders increased from 1990 (80·8 million DALYs [59·5–105·9]; 3·1% [2·4–3·9] of global DALYs), although the age-standardised DALY rates remained largely consistent since 1990 (1581·2 DALYs [1170·9–2061·4] per 100 000 people). Estimated DALYs for mental disorders do not represent fatal burden because they comprised almost entirely YLDs. A total of 125·3 million YLDs (95% UI 93·0–163·2) were attributable to mental disorders, equating to 14·6% (12·2–16·8) of global YLDs in 2019. YLLs were estimated only for eating disorders, which accounted for 17 361·5 YLLs (15 518·5–21 459·8).

**Figure 1:**
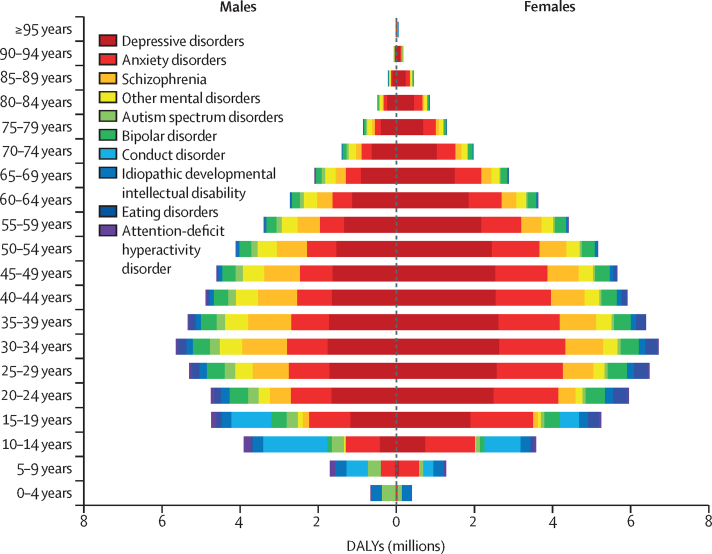
Global DALYs by mental disorder, sex, and age, 2019 DALYs=disability-adjusted life-years.

Globally, the age-standardised DALY rate for mental disorders was 1426·5 (95% UI 1056·4–1869·5) per 100 000 population among males and 1703·3 (1261·5–2237·8) per 100 000 population among females. Depressive disorders accounted for the largest proportion of mental disorder DALYs in 2019 (37·3% [32·3–43·0]), followed by anxiety disorders (22·9% [18·6–27·5]) and schizophrenia (12·2% [9·6–15·2]; figure 1; appendix p 38). Burden due to mental disorders was present across all age groups, emerging before 5 years of age in people with idiopathic intellectual disability and autism spectrum disorders, and continued to be evident at older ages in people with depressive disorders, anxiety disorders, and schizophrenia. Although the relative contribution of each disorder varied by age and sex, the number of DALYs increased steadily during childhood and adolescence, peaked between 25 and 34 years, and decreased steadily after age 35 years. Figure 1 shows global DALYs by disorder, age, and sex in 2019.

**Figure 2:**
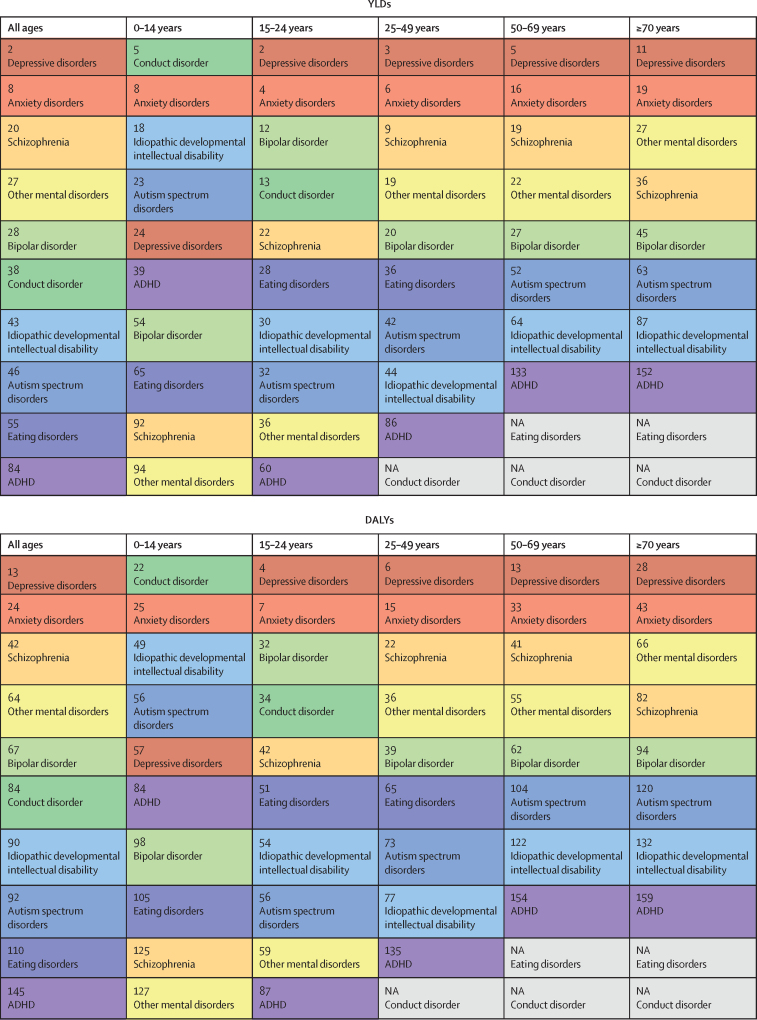
Rankings of YLD and DALY rates for mental disorders by all ages and five age groups for both sexes combined, 2019 Mental disorders were ranked out of all Level 3 causes within the Global Burden of Diseases, Injuries, and Risk Factors Study. Disorders are ordered from highest to lowest ranking for the all ages group. Each colour represents a different mental disorder. Grey cells marked NA show disorders for which burden was not estimated within the age group. ADHD=attention-deficit hyperactivity disorder. DALYs=disability-adjusted life-years. YLDs=years lived with disability.

Globally, mental disorders were the 13th leading cause of DALYs in 1990, and the seventh leading cause of DALYs in 2019 (figure 2). At the disorder level, depressive disorders were ranked 13th among the top 25 leading causes of DALYs in 2019. Mental disorders were the second leading cause of YLDs worldwide in both 1990 and 2019. At the disorder level, of the top 25 leading causes of YLDs in 2019, depressive disorders were ranked second, anxiety disorders ranked eighth, and schizophrenia was ranked 20th. Within mental disorders, depressive disorders ranked the highest in all age groups with the exception of the 0–14-year age category, for which conduct disorder was the leading cause of burden. The rankings of mental disorders differed by sex and age (appendix pp 39, 40).

**Figure 3:**
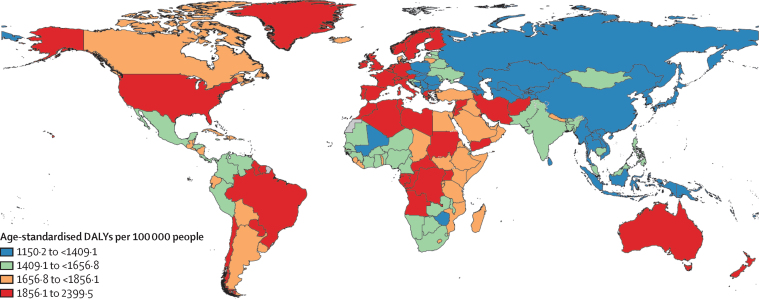
Age-standardised DALYs per 100 000 attributable to mental disorders, 2019 DALYs=disability-adjusted life-years.

The global distribution of mental disorder DALYs in 2019 by country was similar to the trends in prevalence of mental disorders. The highest DALY rates were observed in the USA, Australia, New Zealand, Brazil, selected locations within western Europe (eg, Greenland, Portugal, Greece, Ireland, Spain), sub-Saharan Africa (eg, Uganda), and north Africa and the Middle East (eg, Palestine, Lebanon, Iran; figure 3). The lowest DALY rates were observed in locations in southeast Asia (eg, Vietnam, Myanmar, Indonesia), east Asia (eg, Taiwan [province of China], China, North Korea), high-income Asia Pacific (eg, Brunei), and central Asia (eg, Azerbaijan). Although variation was observed in country-specific DALY rates, they were within overlapping bounds of uncertainty when compared with the global mean (appendix p 41).

## Discussion

In 2019, we observed similar disparities in the global distribution and burden of mental disorders as in 1990. Depressive and anxiety disorders remained among the leading causes of burden worldwide (ranked 13th and 24th leading causes of DALYs, respectively) with prevalence estimates and disability weights comparatively higher than many other diseases. Schizophrenia impacted a smaller proportion of the global population than depressive and anxiety disorders, but the disability weight for an acute state of psychosis was the highest estimated across the GBD study. The persistently high prevalence of these disorders, in addition to bipolar disorder and eating disorders, is especially concerning, because they not only impair health in their own right, but also increase the risk of other health outcomes, such as suicide (rated as the 18th leading cause of mortality in GBD 2019).^5^

We found no marked variation in burden by sex for bipolar disorder and schizophrenia. The burden of depressive disorders, anxiety disorders, and eating disorders was greater in females than males. Burden of autism spectrum disorders and ADHD was greater in males than females. In 2019, 80·6% of the burden due to mental disorders occurred among individuals of working age (16–65 years). Around 9·2% of the remaining burden occurred in people aged younger than 16 years. In 2019, 23·2% of children and adolescents worldwide were located in sub-Saharan Africa, where mental disorders in these age groups pose considerable challenges for economies that already have limited resources dedicated to mental health at a developmental stage when the implementation of prevention and early intervention strategies for mental disorders is crucial.

Overall, DALY rates for mental disorders were high in many high-income countries and were lowest in parts of sub-Saharan Africa and Asia, where the coverage of epidemiological data was lowest, and therefore there is more uncertainty surrounding estimates. Disorder-specific trends were also identified. For example, DALYs for depressive and anxiety disorders were high in countries with high rates of childhood sexual abuse,^4^ intimate partner violence,^4^ and conflict and war.^12^

The age-standardised DALY rates for mental disorders remained fairly constant between 1990 and 2019, but the overall number of DALYs increased by 55·1%. This growth is expected to continue due to population growth and highlights the need for health systems, especially those in low-income and middle-income countries, to deliver the treatment and care needed for this growing population. Effective intervention packages for mental disorders exist. These interventions have the potential to reduce the burden due to mental disorders by decreasing the severity of symptoms, increasing remission, or reducing the risk of mortality.^26^ However, at the global level, there are substantial shortages in access to these services, and in the resources allocated for their scale-up, as well as various barriers to care such as perceived need for care and stigma surrounding mental health problems.^27^, ^28^ In high-income countries where increases in the uptake of treatment for mental disorders have been observed since 1990, treatment is still not reaching minimally adequate standards or those in the population who need it the most.^27^ To reduce the burden of mental disorders, we need to expand the delivery of effective prevention and treatment programmes with established efficacy^26^ to cover more of the population for the necessary duration.

The emergence of the COVID-19 pandemic in 2020 has created an environment in which many determinants of poor mental health outcomes have been exacerbated. Epidemiological research suggests that the direct psychological effects of the pandemic and the long-term impacts on the economic and social circumstances of a population might increase the prevalence of common mental disorders.^29^ Efforts to establish the dataset and methodology from which the impact of the COVID-19 pandemic on the burden of mental disorders can be quantified within the GBD study have been summarised elsewhere.^30^ Our findings demonstrated that mental disorders already imposed a substantial burden before the COVID-19 pandemic. Although it is important to consider the impact of COVID-19 on mental health, the existing unmet mental health needs of the population must also be considered as we focus on recovery from this pandemic. Our GBD 2019 results serve as a stark reminder for countries to re-evaluate their mental health service response more broadly.

The burden estimation methodology for mental disorders used in this study had some key limitations and identified priority areas for improvement. First, despite the considerable amount of new epidemiological data incorporated since our previous GBD 2010 publication on the burden of mental disorders,^6^ some estimates continued to rely on sparse datasets, and high-quality survey data remain scarce for many countries. On the basis of burden of disease analyses done since GBD 2010, we remain concerned about the quality of epidemiological data available for mental disorders. Our systematic literature review made use of inclusion criteria imposing minimum standards to data collection methodology across studies. We recommend that these standards be considered by researchers undertaking new mental health surveys, specifically with regard to decisions around case definitions, instruments, sampling strategy, and standard of reporting.

Second, it was difficult to quantify and remove all variation due to measurement error in our prevalence estimates. We corrected for known sources of bias caused by survey methods but few datapoints were available to inform such adjustments for some disorders and other important sources of variation in prevalence remain unquantified. For example, it is difficult to disentangle reasons for cross-national differences in our burden estimates. The importance of cross-culturally comparable case definitions and case-finding for mental disorders has been emphasised,^31^ but the epidemiological data informing burden estimates are limited in this respect. The use of DSM and ICD classifications, which ensures consistency in case definitions across studies, might not be sensitive to all cultural contexts.^32^ The cross-cultural applicability of our case definitions and data collection methodology need to be considered in future research. The uncertainty intervals reported here do not incorporate these sources of bias that are difficult to quantify, including measurement bias not captured by our bias corrections, selection bias due to missing data, and model specification bias.

Third, our estimation of severity distributions was derived from few studies, mostly from high-income countries. Imposing severity distributions from high-income countries to all locations is likely to have underestimated burden in countries with little or no access to treatment and needs to be reconsidered. Raw data on the severity distribution of mental disorders by location that would facilitate this work is not available. However, alternative work to model the impact of access to health care on the severity of mental disorders is ongoing within the GBD study.

Fourth, the majority of the epidemiological data within our datasets adhered to DSM-IV and ICD-10 diagnostic classifications. With the emergence of more epidemiological surveys using DSM-5 and ICD-11 classifications, work to account for the impact of changes to diagnostic classifications within our GBD estimates will be undertaken.

Fifth, the mental disorders included in GBD 2019 were those with sufficient epidemiological data at a global level required for burden of disease analysis. Personality disorders were captured through the residual group of other mental disorders in GBD 2019, with limited sources available to inform their prevalence and disability weight analysis. Binge eating disorder and the group of ‘other specified feeding or eating disorders’ are likely to explain a substantial proportion of eating disorder burden currently not captured by GBD analyses.^33^ Efforts to compile the required datasets and analyses for formal inclusion of these disorders in the GBD study is underway.

Sixth, our study did not consider substance use disorders or neurological disorders. An evaluation of the burden imposed by this broader group of disorders was done for the 2016 review of Disease Control Priorities.^26^

Seventh, the differential mortality gap for individuals with mental disorders needs to be reflected within the GBD framework. Within the mental disorder group, deaths were estimated for only eating disorders. These estimated deaths are extremely low, and not reflective of premature mortality in individuals with eating disorders, or with other mental disorders where the direct cause of death is another disease or injury. Alternative mortality-based metrics have shown that excess deaths in people with mental disorders occur not just from suicide and other external causes but also from infectious diseases, neoplasms, diabetes, and circulatory system and respiratory diseases.^34^, ^35^ These deaths are assigned to those causes within the GBD 2019. A method for capturing the proportion of premature deaths from physical health causes that can be causally attributed to the mental disorder experienced by a person is not yet available for our estimation of YLLs. However, where the evidence exists, it is feasible to use comparative risk assessment to quantify the contribution of mental disorders to premature mortality. Supplementary GBD 2010 analyses found that the inclusion of attributable suicide DALYs would have increased the overall burden of mental and substance use disorders from 7·4% to 8·3% of all global DALYs, increasing their global ranking from fifth to third.^36^ An update to this work using GBD 2020 estimates is in progress, and the first publication using the application of meta-regression techniques to summarise the relative-risk of mental disorders as risk factors for suicide is available.^37^ Further work to establish causal pathways between mental disorders and other health outcomes is required to enable replication of this analysis for other fatal outcomes within the GBD study.

Eighth, broader limitations in the GBD study should be acknowledged. Our definition of disability reflects health loss but not welfare loss. Estimates therefore do not capture the full impact of mental disorders on society. Disability weights were derived from brief descriptions of disease states that might not capture the full complexity of symptoms, across settings. Replication of the disability weight survey across more locations, containing more lay descriptions associated with mental disorders, is required to investigate the generalisability of estimates. We assume independent distributions of comorbid conditions when adjusting YLDs for comorbidity within GBD 2019. This assumption is a limitation especially for mental disorders since comorbidity distributions might be dependent on the combination of disorders experienced. Efforts to incorporate dependent comorbidity within the GBD study have been challenging owing to scarcity of data to inform the correlation structure of prevalence consistently for all diseases and injuries. Even within mental disorders, additional research is required as information on dependent comorbidity is available for only a small subset of possible combinations of disorders and is limited to specific age groups and populations.

The findings of GBD 2019 emphasise the large proportion of the global disease burden attributable to mental disorders and the global disparities in that burden. Furthermore, there was no evidence of global reduction in the burden since 1990, despite evidence-based interventions that can reduce the burden across age, sex, and geographical locations. The ongoing impact of the COVID-19 pandemic is likely to increase the global burden of mental disorders. A coordinated response by governments and the global health community is urgently needed to address the present and future mental health treatment gap.

Correspondence to: Dr Alize Ferrari, Queensland Centre for Mental Health Research, University of Queensland, Brisbane, QLD 4108, Australia **a.ferrari@uq.edu.au**

## Data sharing

To download the data used in these analyses, please visit the Global Health Data Exchange GBD 2019 website.

## Declaration of interests

C Kieling reports grants from MQ: Transforming Mental Health, the Royal Academy of Engineering, the US Academy of Medical Sciences, the US National Institutes of Health, Conselho Nacional de Desenvolvimento Científico e Tecnológico, the UK Medical Research Council, and Fundação de Amparo à Pesquisa do Estado do Rio Grande do Sul; and consulting fees from the United Nations Children's Fund, outside the submitted work. P B Mitchell reports grants from the Australian National Health and Medical Research Council; and payment or honoraria for lectures, presentations, speakers bureaus, manuscript writing, or educational events from Janssen Australia, outside the submitted work. G C Patton reports support for the present manuscript from the Australia National Health and Medical Research Council. J B Soriano reports participation in the Institute for Health Metrics and Evaluation's Tobacco Advisory board, outside the submitted work. D J Stein reports royalties or licenses from Elsevier and the American Psychiatric Press; consulting fees from Johnson & Johnson, Lundbeck, Sanofi, and Vistagen; and payment or honoraria for lectures, presentations, speakers bureaus, manuscript writing or educational events from Servier and Takeda, outside the submitted work. M B Stein reports grants or contracts from the US National Institute of Mental Health, US Department of Defense, and US Department of Veterans Affairs; consulting fees from Aptinyx, Acadia Pharmaceuticals, Bionomics, Boehringer-Ingelheim, Clexio, EmpowerPharm, Engrail, GW Pharmaceuticals, Janssen, Kazz Pharmaceuticals, and Roche/Genentech; stocks from Pfizer; holds stock options in Epivario and Oxeia Biopharmaceuticals, and owns mutual funds that might contain pharmaceutical stocks; and is the Editor-in-Chief of *Depression and Anxiety*, Deputy Editor of *Biological Psychiatry*, and Co-Editor-in-Chief of UptoDate (Psychiatry), outside the submitted work. C E I Szoeke acknowledges support for the present manuscript from National Health and Medical Research Council Australia funding (1032350 and 1062133) paid to the University of Melbourne; and acknowledges payment for expert testimony from the Victorian Department of Health, and for leadership or fiduciary role in board, society, committee or advocacy group, paid or unpaid with the American Medical Association, outside the submitted work. All other authors declare no competing interests.
