# Supplementary material for: Global, regional, and national burden of 12 mental disorders in 204 countries and territories, 1990–2019: a systematic analysis for the Global Burden of Disease Study 2019
Source: Lancet Psychiatry. 2022 Feb;9(2):137–50. doi: 10.1016/S2215-0366(21)00395-3 (PMC8776563; doi:10.1016/S2215-0366(21)00395-3)
Supplement: Supplementary appendix [file mmc1.pdf]

THE LANCET
Psychiatry

Supplementary appendix

This appendix formed part of the original submission and has been peer reviewed. We post it as supplied by the authors.

Supplement to: GBD 2019 Mental Disorders Collaborators. Global, regional, and national burden of 12 mental disorders in 204 countries and territories, 1990–2019: a systematic analysis for the Global Burden of Disease Study 2019. *Lancet Psychiatry* 2022; published online Jan 10. https://doi.org/10.1016/S2215-0366(21)00395-3.

Appendix

eTable 1: GATHER checklist of information included, with description of compliance and location of information for GBD 2019

eTable 2: Mental disorders included in GBD 2019

eFigure 1: GBD 2019 cause hierarchy for mental disorders

eFigure 2: Summary of non-fatal burden estimation methodology for mental disorders

eTable 3: Keywords used in the systematic literature searches

eTable 4: Priors used to inform the DisMod-MR 2.1 models for each mental disorder

eTable 5: GBD 2019 sequela-specific health state descriptions, disability weights, and severity splits for mental disorders

eTable 6: Total data sources included in the GBD 2019 estimation of DALYs for each mental disorder

eTable 7: Prevalent cases in thousands, with 95% uncertainty intervals, by mental disorder and location in 2019

eTable 8: Age-standardised prevalence per 100,000, with 95% uncertainty intervals, by mental disorder and location in 2019

eFigure 3: Proportion of global mental disorder DALYs attributable to each disorder for both sexes and all ages in 2019

eTable 9a: All-cause rankings of YLD and DALY rates for mental disorders for males by all ages and five age groups in 2019

eTable 9b: All-cause rankings of YLD and DALY rates for mental disorders for females by all ages and five age groups in 2019

eTable 10: Age-standardised rates and number of DALYs for mental disorders by location in 2019

etext1: Author contributions

eTable 1: GATHER checklist of information included, with description of compliance and location of information for GBD 2019

#	GATHER checklist item	Description of compliance	Reference
**Objectives and funding**			
1	Define the indicators, populations, and time periods for which estimates were made.	Narrative provided in paper and appendix describing indicators, definitions, and populations	Main text (Methods) and appendix
2	List the funding sources for the work.	Funding sources listed in paper	Main text (Methods)
**Data Inputs**			
For all data inputs from multiple sources that are synthesised as part of the study:			
3	Describe how the data were identified and how the data were accessed.	Narrative description of data seeking methods provided	Main text (Methods) and appendix
4	Specify the inclusion and exclusion criteria. Identify all ad-hoc exclusions.	Narrative about inclusion and exclusion criteria by data type provided; ad hoc exclusions in cause-specific write-ups	Main text (Methods) and appendix
5	Provide information on all included data sources and their main characteristics. For each data source used, report reference information or contact name/institution, population represented, data collection method, year(s) of data collection, sex and age range, diagnostic criteria or measurement method, and sample size, as relevant.	An interactive, online data source tool that provides metadata for data sources by component, geography, cause, risk, or impairment has been developed	Data Input Sources Tool: http://ghdx.healthdata.org/gbd-2019/data-input-sources
6	Identify and describe any categories of input data that have potentially important biases (e.g., based on characteristics listed in item 5).	Summary of known biases by cause included in appendix	Main text (Methods and Limitations) and appendix
For data inputs that contribute to the analysis but were not synthesised as part of the study:			
7	Describe and give sources for any other data inputs.	Included in online data source tool	Data Input Sources Tool: http://ghdx.healthdata.org/gbd-2019/data-input-sources
For all data inputs:			
8	Provide all data inputs in a file format from which data can be efficiently extracted (e.g., a spreadsheet as opposed to a PDF), including all relevant meta-data listed in item 5. For any data inputs that cannot be shared due to ethical or legal reasons, such as third-party ownership, provide a contact name or the name of the institution that retains the right to the data.	Downloads of input data available through online tools, including data visualisation tools and data query tools; input data not available in tools will be made available upon request	GBD 2019 Data Resources: http://ghdx.healthdata.org/gbd-2019
**Data analysis**			
9	Provide a conceptual overview of the data analysis method. A diagram may be helpful.	Flow diagrams of the overall methodological processes, as well as cause-specific modelling processes, have been provided	Main text (Methods) and appendix
10	Provide a detailed description of all steps of the analysis, including mathematical formulae. This description should cover, as relevant, data cleaning, data pre-processing, data adjustments and weighting of data sources, and mathematical or statistical model(s).	Flow diagrams and corresponding methodological write-ups for each cause, as well as the databases and modelling processes, have been provided	Main text (Methods) and appendix
11	Describe how candidate models were evaluated and how the final model(s) were selected.	Provided in the methodological write-ups	Previously published in capstone paper appendices
12	Provide the results of an evaluation of model performance, if done, as well as the results of any relevant sensitivity analysis.	Provided in the methodological write-ups	Previously published in capstone paper appendices
13	Describe methods for calculating uncertainty of the estimates. State which sources of uncertainty were, and were not, accounted for in the uncertainty analysis.	Appendix	Methods and previously published in capstone paper appendices
14	State how analytic or statistical source code used to generate estimates can be accessed.	Appendix	http://ghdx.healthdata.org/gbd-2019/code
**Results and Discussion**			
15	Provide published estimates in a file format from which data can be efficiently extracted.	GBD 2019 results are available through online	Main text, appendix and GBD 2019 Data Resources: http://ghdx.healthdata.org/gbd-

		data visualisation tools,<br>the Global Health Data<br>Exchange, and the online data query tool	2019
16	Report a quantitative measure of the uncertainty of the estimates (e.g. uncertainty intervals).	Uncertainty intervals are provided with all results	Main text, appendix, and GBD 2019 Data Resources: http://ghdx.healthdata.org/gbd-2019
17	Interpret results in light of existing evidence. If updating a previous set of estimates, describe the reasons for changes in estimates.	Discussion of methodological changes<br>between GBD rounds<br>provided in the narrative<br>of the manuscript and appendix	Main text (Methods and Discussion) and appendix
18	Discuss limitations of the estimates. Include a discussion of any modelling assumptions or data limitations that affect interpretation of the estimates.	Discussion of limitations<br>provided in the narrative of the main paper, as well as in the methodological write-ups<br>in the appendix	Main text (Limitations) and appendix

eTable 2: Mental disorders included in GBD 2019

Cause	Definition	Corresponding DSM-IV-TR and ICD-10 codes
Major depressive disorder	Involves the presence of at least one major depressive episode, which is the experience of either depressed mood or loss of interest/pleasure, for most of every day, for at least two weeks.	DSM-IV-TR: 296.21–24, 296.31–34<br>ICD-10: F32.0–9, F33.0–9
Dysthymia	Involves the experience of chronically depressed mood for most of the day, more days than not, for at least two years (or at least one year in children and adolescents).	DSM-IV-TR: 300.4<br>ICD-10: F34.1
Anxiety disorders	Involves experiences of intense fear and distress, typically in combination with other physiological symptoms. Anxiety disorders were modelled as a single cause for "any" anxiety disorder to avoid the double-counting of individuals meeting criteria for more than one anxiety disorder. Epidemiological estimates reporting an outcome for "any" or "total" anxiety disorders were included if they reported on at least three anxiety disorders.	DSM-IV-TR: 300.0-300.3, 208.3, 309.21, 309.81<br>ICD-10: F40-42, F43.0, F43.1, F93.0-93.2, F93.8.
Schizophrenia	Involves the experience of positive symptoms (e.g., delusions, hallucinations, thought disorder) and negative symptoms (e.g., flat affect, loss of interest, and emotional withdrawal).	DSM-IV-TR: 295.10-295.30, 295.60, 295.90<br>ICD 10: F20
Bipolar disorder	Bipolar I is characterised by at least one manic episode, which can also alternate with a major depressive episode. Bipolar II is characterised by hypomanic episodes alternating with major depressive episodes. Cyclothymia is characterised by subsyndromal hypomanic and major depressive episode. Bipolar disorder not otherwise specified is characterised by clinically significant symptoms of bipolar disorder which do not meet criteria for the other diagnoses. We estimated burden for the entire spectrum of bipolar disorder simultaneously, rather than individually for each subtype of the disorder. At a minimum, epidemiological studies needed to report on bipolar I and bipolar II.	DSM-IV-TR: 296.0–296.7, 296.89, 301.13<br>ICD-10: F30.0-F30.9, F31.0–F31.6, F31.8–F31.9, F34.0
Anorexia nervosa	Characterised by refusal to maintain body weight at or above a minimally normal weight for age and height, intense fear of gaining weight, and disturbance in the way in which one's body weight or shape is experienced.	DSM-IV-TR: 307.1<br>ICD-10: F50.0-50.1
Bulimia nervosa	Characterised by recurrent episodes of binge eating and inappropriate compensatory behaviour to prevent weight gain. These must occur, on average, at least twice a week for three months.	DSM-IV-TR: 307.51<br>ICD-10: F50.2
Conduct disorder	Occurs in those under 18 years of age and is characterised by a pattern of antisocial behaviour that violates the basic rights of others or major age-appropriate societal norms.	DSM-IV-TR: 312.81-312.89<br>ICD-10: F91
Attention-deficit/hyperactivity disorder	Characterised by persistent inattention and/or hyperactivity-impulsivity. Diagnosis requires six or more symptoms of inattention or hyperactivity-impulsivity to have persisted for at least six months, in two or more settings; with at least some impairing symptoms being present prior to 7 years of age.	DSM-IV-TR: 314.0, 314.01<br>ICD-10: F90
Autism Spectrum Disorder	Characterised by pervasive impairment in several areas of development, including social interaction and communication skills, along with restricted and repetitive patterns of behaviours and/or interests. Symptoms must be present in the early developmental period, cause clinically significant impairment, and not be better explained by intellectual impairment or global developmental delay.	DSM-IV-TR: 299.00, 299.80, 299.8, 299.8, 299.10<br>ICD-10: F84.0, F84.1, F84.2, F84.3, F84.4, F84.5, F84.8, F84.9
Other mental disorders	A residual cause within GBD which incorporates disability from an aggregate group of personality disorders. Personality disorders are characterised by pervasive, inflexible and maladaptive patterns of behaviour and inner experience which are markedly different from what is considered to be acceptable in the individual's culture.	DSM-IV-TR: 300.3, 301.0; 301.2, 301.22, 301.5–301.9<br>ICD-10: F60
Idiopathic developmental intellectual disability	Idiopathic developmental intellectual disability modelled as part of the intellectual disability impairment envelop in GBD 2019. Idiopathic developmental intellectual disability arises from any unknown source after the prevalence of all other sources of Intellectual Disability is accounted for.	Involves borderline (IQ Score 70-85), mild (IQ score 50 to 69), moderate (IQ Score 35-49), severe (IQ score 20-34), and profound (IQ score 0-19) idiopathic intellectual disability.

Note: DSM-IV-TR: Diagnostic and Statistical Manual of Mental Disorders Fourth Edition, Text Revision; ICD-10: International Classification of Diseases and Related Health Problems 10th Revision.

eFigure 1: GBD 2019 cause hierarchy for mental disorders

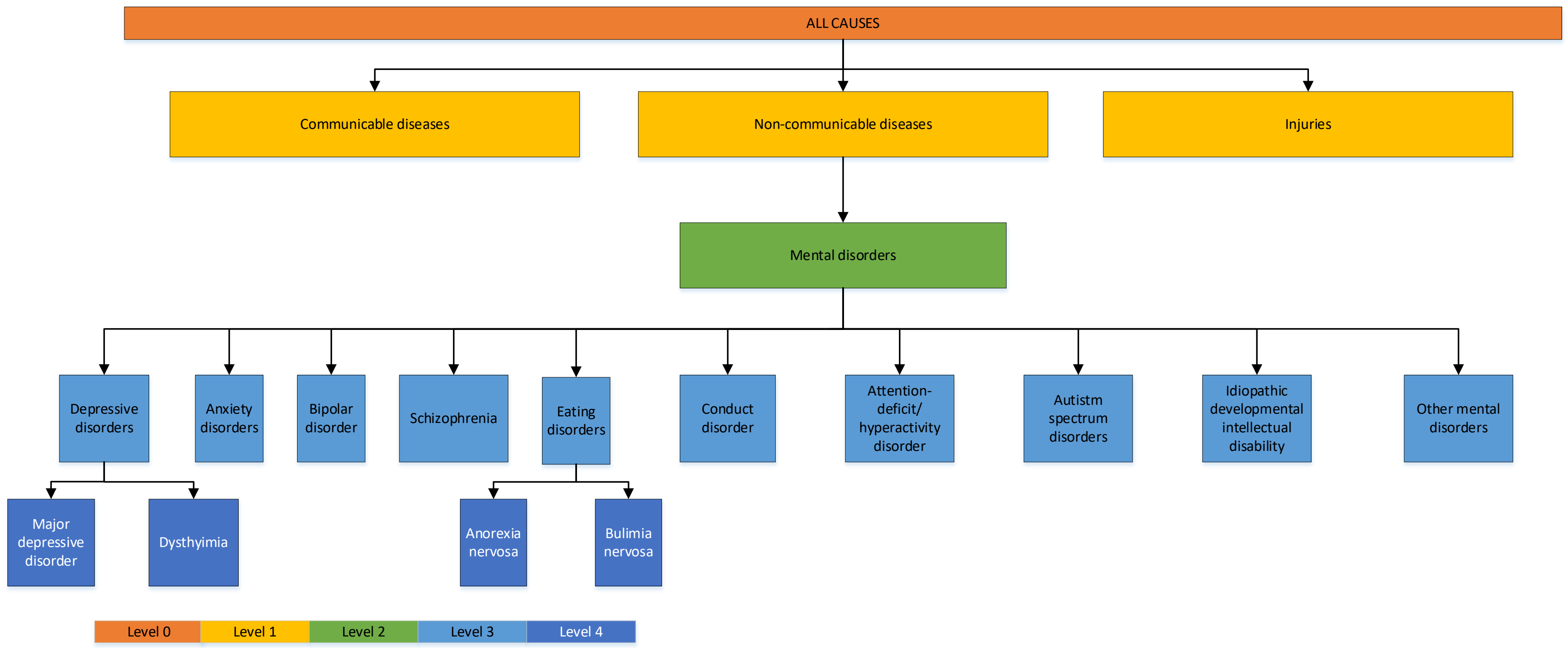

eFigure 2: Summary of non-fatal burden estimation methodology for mental disorders

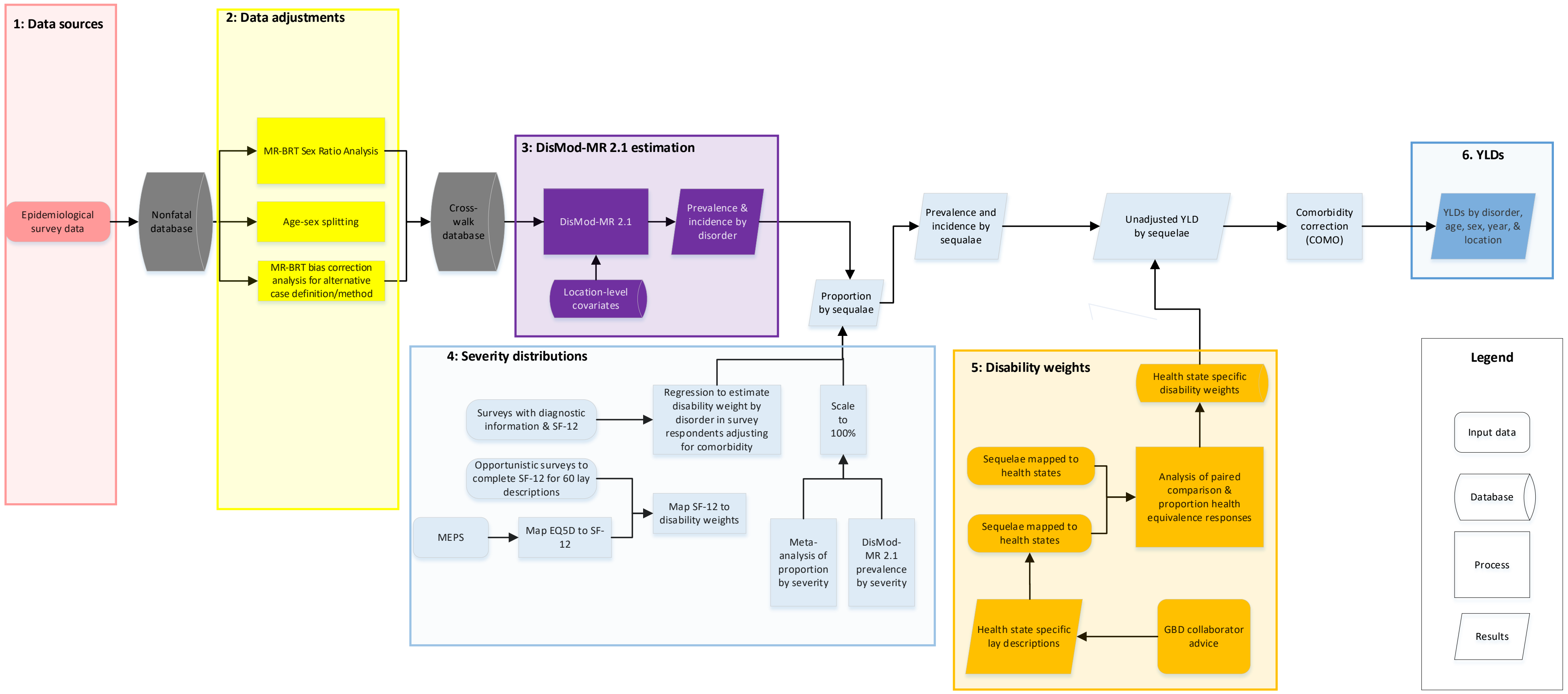

Note. MR-BRT: meta-regression—Bayesian, regularised, trimmed; CoD: Cause of death; SF-12: Short Form 12; MEPS: Medical Expenditure Panel Survey; EQ5D: European Quality of Life-5 Dimension; YLDs: years lived with disability.

eTable 3: Keywords used in the systematic literature searches

Disorder	Dates searched	Keyword
Major depressive disorder,<br>Dysthymia,<br>Bipolar disorder	1st January 1980 to 26th April 2018	((depress*[Title/Abstract]) OR dysthymi*[Title/Abstract]) OR bipolar[Title/Abstract]) OR manic[Title/Abstract]) OR mania[Title/Abstract]) OR "mood disorders"[Title/Abstract]) OR "mood disorder"[Title/Abstract]) OR mood disorders[MeSH Terms]) OR depressive disorders[MeSH Terms]) OR depressive disorders, major[MeSH Terms]) OR bipolar disorders[MeSH Terms]) OR dysthymic disorders[MeSH Terms])) **AND** ((inciden*[Title/Abstract]) OR prevalen*[Title/Abstract]) OR mortalit*[Title/Abstract]) OR death*[Title/Abstract]) OR recurren*[Title/[Abstract]) OR (remission[Title/[Abstract]) OR (remit*[Title/Abstract]) OR (duration [Title/Abstract]) OR (epidemiolog*[Title/Abstract]) OR "prevalence"[Mesh]) OR "mortality*"[Mesh]) OR "incidence"[Mesh]) OR "epidemiology"[Mesh:NoExp]) OR "morbidity"[Mesh:NoExp))) Filters: Publication date from 2016/09/01"[PDat] : "3000/12/31"[PDat]))
Anxiety disorders	1st January 1980 to 5th November 2018	(("agoraphobias"[Title/Abstract] OR "specific phobia"[Title/Abstract] OR "specific phobias"[Title/Abstract] OR "panic disorder"[Title/Abstract] OR "panic disorders"[Title/Abstract] OR "agoraphobia"[Title/Abstract] OR "social phobia"[Title/Abstract] OR "social phobias"[Title/Abstract] OR "generalized anxiety disorder"[Title/Abstract] OR "generalized anxiety disorders"[Title/Abstract] OR "obsessive compulsive disorder"[Title/Abstract] OR "obsessive compulsive disorders"[Title/Abstract] OR "ocd"[Title/Abstract] OR "generalised anxiety disorder"[Title/Abstract] OR "generalised anxiety disorders"[Title/Abstract] OR "ptsd"[Title/Abstract] OR "post traumatic stress disorder"[Title/Abstract] OR "post traumatic stress disorders"[Title/Abstract] OR "posttraumatic stress disorder"[Title/Abstract] OR "anxiety disorders"[Title/Abstract] OR "anxiety disorder"[Title/Abstract] OR "Anxiety Disorders"[Mesh] **AND** ((inciden*[Title/Abstract]) OR prevalen*[Title/Abstract]) OR mortalit*[Title/Abstract]) OR death*[Title/Abstract]) OR recurren*[Title/[Abstract]) OR (remission[Title/[Abstract]) OR (remit*[Title/Abstract]) OR (duration [Title/Abstract]) OR (epidemiolog*[Title/Abstract]) OR "prevalence"[Mesh]) OR "mortality*"[Mesh]) OR "incidence"[Mesh]) OR "epidemiology"[Mesh:NoExp]) OR "morbidity"[Mesh:NoExp))) Filters: Publication date from 2016/09/01"[PDat] : "3000/12/31"[PDat]))
Schizophrenia	1st January 1980 to 30th November 2018	((Schizophrenia[Title]) **AND** (((((epidemiology) OR epidemiology [MeSH Terms]) OR prevalence[Title/Abstract]) OR incidence[Title/Abstract]) OR mortality[Title/Abstract]) OR remission[Title/Abstract])) AND (("2013"[Date - Publication] : "2014"[Date - Publication]).
Anorexia nervosa,<br>bulimia nervosa	1st January 1980 to 30th November 2018	(("anorexia nervosa"[Title/Abstract]) OR "bulimia nervosa"[Title/Abstract]) OR "eating disorders"[Title/Abstract]) OR "eating disorder"[Title/Abstract]) OR "binge eating disorder"[Title/Abstract]) OR anorexic[Title/Abstract]) OR bulimic[Title/Abstract]) OR binge eating disorder[MeSH Terms]) OR anorexia nervosa[MeSH Terms]) OR bulimia nervosa[MeSH Terms]) OR eating disorders[MeSH Terms] **AND** ((epidemiology[Title/Abstract]) OR (incidence[Title/Abstract]) OR (prevalence[Title/Abstract]) OR (mortality[Title/Abstract]) OR (remission[Title/Abstract]))
Autism spectrum disorders	1st January 1980 to 11th August 2017	((autis*[Title/Abstract]) OR asperger*[Title/Abstract]) OR ((pervasive[Title/Abstract]) AND disorder*[Title/Abstract]))) OR "Child Development Disorders, Pervasive"[Mesh])) **AND** ((prevalen*[Title/Abstract]) OR mortalit*[Title/Abstract]) OR death*[Title/Abstract]) OR inciden*[Title/Abstract]) OR remission[Title/Abstract]) OR duration[Title/Abstract]) OR remit*[Title/Abstract]) OR epidemiolog*[Title/Abstract]) OR "Prevalence"[Mesh]) OR "Mortality"[Mesh]) OR "Incidence"[Mesh]) OR "Epidemiology"[Mesh:NoExp]) OR "Morbidity"[Mesh:NoExp])
Attention-deficit/hyperactivity disorder,<br>Conduct disorder	1st January 1980 to 10th October 2018	((attention deficit and disruptive behavior disorders[MeSH Terms]))) OR (((attention*[Title/Abstract]) AND disorder[Title/Abstract]) AND hyperactiv*[Title/Abstract])) OR hyperkinetic[Title/Abstract]) OR adhd[Title/Abstract]) OR conduct disorder[Title/Abstract]) OR conduct disorders[Title/Abstract]) OR ((disruptive[Title/Abstract]) AND disorder[Title/Abstract])) OR externali*[Title/Abstract] **AND** ((prevalen*[Title/Abstract]) OR mortalit*[Title/Abstract]) OR death*[Title/Abstract]) OR inciden*[Title/Abstract]) OR remission[Title/Abstract]) OR duration[Title/Abstract]) OR remit*[Title/Abstract]) OR epidemiolog*[Title/Abstract]) OR "Prevalence"[Mesh]) OR "Mortality"[Mesh]) OR "Incidence"[Mesh]) OR "Epidemiology"[Mesh:NoExp]) OR "Morbidity"[Mesh:NoExp] Filters: Publication date from 2016/09/01"[PDat] : "3000/12/31"[PDat]))

Note: Idiopathic developmental intellectual disability was modelled as part of the intellectual disability impairment envelope. Data for other mental disorders were sourced from three national surveys.

eTable 4: Priors used to inform the DisMod-MR 2.1 models for each mental disorder

Disorder	Incidence	Remission	Excess Mortality
Major depressive disorder	0 for ages 0 to 3	1.3 to 1.6 for ages 0 to 100	-
Dysthymia	0 for ages 0 to 3	0 to 0.2 for ages 0 to 100	0 for ages 0 to 100
Anxiety disorders	0 for ages 0 to 2<br>0 for ages 95 to 100	0 to 0.2 for ages 0 to 100	0 for ages 0 to 100
Schizophrenia	0 for ages 0 to 10<br>0 for ages 80 to 100	0 to 0.04 for ages 0 to 100	0 for ages 0 to 10
Bipolar disorder	0 for ages 0 to 10	0 to 0.05 for ages 0 to 100	0 to 0.1 for ages 0 to 100
Anorexia nervosa	0 for ages 0 to 4<br>0 for ages 50 to 100	0 to 0.6 for ages 4 to 50	-
Bulimia nervosa	0 for ages 0 to 9<br>0 for ages 40 to 100	-	-
Attention-deficit/ hyperactivity disorder	0 for ages 0 to 2<br>0 for ages 12 to 100	0 for ages 0 to 11	0 for ages 0 to 100
Conduct disorder	0 for ages 0 to 4<br>0 to 0.03 for ages 4 to 18<br>0 for ages 18 to 100	0 to 0.4 for ages 4 to 17	0 for ages 0 to 100
Autism spectrum disorder	0 for ages 0 to 100	0 for ages 0 to 100	0 to 0.01 for ages 0 to 100
Other mental disorders	0 for ages 0 to 14	0 to 0.01 for ages 0 to 100	0 for ages 0 to 100

Note. Priors were informed by the following considerations: case definitions for mental disorders, age limits observed within the raw data for each parameter, the extent to which the prior was able to guide the modelled outputs towards the raw data. All priors were reviewed in consultation with disorder experts.

eTable 5: GBD 2019 sequela-specific health state descriptions, disability weights, and severity splits for mental disorders

Health state	Lay health state description	Disability weight	Severity split
**Major depressive disorder**			
Asymptomatic	Implying a disability weight of 0 after comorbidity adjustments.	0	13% (10%-17%)
Mild	Feels persistent sadness and has lost interest in usual activities. The person sometimes sleeps badly, feels tired, or has trouble concentrating but still manages to function in daily life with extra effort.	0.145 (0.099-0.209)	59% (49%-69%)
Moderate	Has constant sadness and has lost interest in usual activities. The person has some difficulty in daily life, sleeps badly, has trouble concentrating, and sometimes thinks about harming himself (or herself).	0.396 (0.267-0.531)	17% (13%-22%)
Severe	Has overwhelming, constant sadness and cannot function in daily life. The person sometimes loses touch with reality and wants to harm or kill himself (or herself).	0.658 (0.477-0.807)	10% (3%-20%)
**Dysthymia**			
Asymptomatic	Implying a disability weight of 0 after comorbidity adjustments.	0	29% (23%-36%)
Symptomatic dysthymia[a]	Feels persistent sadness and has lost interest in usual activities. The person sometimes sleeps badly, feels tired, or has trouble concentrating but still manages to function in daily life with extra effort.	0.145 (0.099-0.209)	71% (64%-77%)
**Bipolar disorder**			
Manic/hypomanic	Is hyperactive, hears and believes things that are not real, and engages in impulsive and aggressive behaviour that endangers the person and others.	0.492 (0.341-0.646)	21% (12%-33%),
Depressive[b]	Has constant sadness and has lost interest in usual activities. The person has some difficulty in daily life, sleeps badly, has trouble concentrating, and sometimes thinks about harming himself (or herself).	0.396 (0.267-0.531)	23% (10%-39%)
Residual	Has mild mood swings, irritability, and some difficulty with daily activities.	0.032 (0.018-0.051)	52% (28%-77%)
**Anxiety disorders**			
Asymptomatic	Implying a disability weight of 0 after comorbidity adjustments.	0	29% (28%-30%)
Mild	Feels mildly anxious and worried, which makes it slightly difficult to concentrate, remember things, and sleep. The person tires easily but is able to perform daily activities.	0.03 (0.018-0.046)	39% (34%-44%)
Moderate	Feels anxious and worried, which makes it difficult to concentrate, remember things, and sleep. The person tires easily and finds it difficult to perform daily activities.	0.133 (0.091-0.186)	19% (16%-23%)
Severe	Constantly feels very anxious and worried, which makes it difficult to concentrate, remember things, and sleep. The person has lost pleasure in life and thinks about suicide.	0.523 (0.362-0.677)	13% (9%-17%)
**Schizophrenia**			
Acute	Hears and sees things that are not real and is afraid, confused, and sometimes violent. The person has great difficulty with communication and daily activities, and sometimes wants to harm or kill himself (or herself).	0.778 (0.606-0.9)	63% (29%-91%)
Residual	Hears and sees things that are not real and has trouble communicating. The person can be forgetful, has difficulty with daily activities, and thinks about hurting himself (or herself).	0.588 (0.411-0.754)	37% (9%-71%)
**Anorexia nervosa**			
	Feels an overwhelming need to starve and exercises excessively to lose weight. The person is very thin, weak, and anxious.	0.224 (0.150-0.312)	-
**Bulimia nervosa**			
	Has uncontrolled overeating followed by guilt, starving, and vomiting to lose weight.	0.223 (0.149-0.311)	-
**Autism**			
	Has severe problems interacting with others and difficulty understanding simple questions or directions. The person has great difficulty with basic daily activities and becomes distressed by any change in routine.	0.262 (0.176-0.365)	43% (36%-51%)
**Asperger's syndrome and other autism spectrum disorders**			
	Has difficulty interacting with other people and is slow to understand or respond to questions. The person is often preoccupied with one thing and has some difficulty with basic daily activities.	0.104 (0.071-0.147)	57% (49%-64%)
**Attention-deficit/hyperactivity disorder**			
Asymptomatic	Implying a disability weight of 0 after comorbidity adjustments.	0	72% (65%-79%)
Symptomatic ADHD	Is hyperactive and has difficulty concentrating, remembering things, and completing tasks.	0.045 (0.028-0.066)	28% (21%-35%)
**Conduct disorder**			
Asymptomatic	Implying a disability weight of 0 after comorbidity adjustments.	0	48% (35%-61%)
Symptomatic conduct disorder	Has frequent behaviour problems, which are sometimes violent. The person often has difficulty interacting with other people and feels irritable.	0.241 (0.159-0.341)	52% (39%-65%)
**Idiopathic developmental intellectual disability**			

Asymptomatic	Implying a disability weight of 0 after comorbidity adjustments.	0	c
Borderline IDII	Is slow in learning at school. As an adult, the person has some difficulty doing complex or unfamiliar tasks but otherwise functions independently.	0.011 (0.005-0.02)	c
Mild IDII	Has low intelligence and is slow in learning at school. As an adult, the person can live independently, but often needs help to raise children and can only work at simple supervised jobs	0.043 (0.026-0.064)	c
Moderate IDII	Has low intelligence and is slow in learning to speak and to do even simple tasks. As an adult, the person requires a lot of support to live independently and raise children. The person can only work at the simplest supervised jobs.	0.100 (0.066-0.142)	c
Severe IDII	Has very low intelligence and cannot speak more than a few words, needs constant supervision and help with most daily activities, and can do only the simplest tasks.	0.160 (0.107-0.226)	c
Profound IDII	Has very low intelligence, has almost no language, and does not understand even the most basic requests or instructions. The person requires constant supervision and help for all activities.	0.200 (0.133-0.283)	c

Note: IDII = idiopathic developmental intellectual disability. Severity proportions for other mental disorders used the severity distributions from NESARC and the 1997 Australian National Survey of Mental Health and Wellbeing. [a]Equivalent to the disability weight estimated for mild major depressive disorder. [b]Equivalent to the disability weight estimated for moderate major depressive disorder. ADHD=attention-deficit/hyperactivity disorder. [c]A meta-analysis of severity levels of the ID envelope is conducted with data on proportion of those with IQ<70 who have an IQ from 50-69, and then for those with IQ <50 whose IQ is <20, 20-34 and 35-49. For IIDI, this is the subtraction of all secondary causes of ID, each with their own source of severity levels.

eTable 6: Total data sources included in the GBD 2019 estimation of DALYs for each mental disorder

Disorder	Prevalence	Incidence	Remission	Causes of death	Other*
Major depressive disorder	492	2	0	0	24
Dysthymia	104	1	2	0	1
Bipolar disorder	113	2	0	0	42
Anxiety disorders	199	1	3	0	16
Schizophrenia	142	16	8	0	48
Anorexia nervosa	65	6	21	1908	17
Bulimia nervosa	66	4	10	1431	6
Autism spectrum disorders	164	0	0	0	3
Attention-deficit/hyperactivity disorder	172	2	14	0	3
Conduct disorder	49	1	1	0	2
Other mental disorders	3	0	0	0	0
Idiopathic developmental intellectual disability	58	0	0	0	0

Note. Other data sources include data from other epidemiological parameters such as disease duration and severity.

eTable 7: Prevalent cases in thousands, with 95% uncertainty intervals, by mental disorder and location in 2019

Location	Schizophrenia	Major depressive disorder	Dysthymia	Bipolar disorder	Anxiety disorders	Anorexia nervosa	Bulimia nervosa	Autism spectrum disorders	Attention-deficit/hyperactivity disorder	Conduct disorder	Idiopathic developmental intellectual disability	Other mental disorders
**Global**	**23597·0 (20233·3–27150·7)**	**185152·6 (162588·0–210149·9)**	**100489·1 (83695·6–121208·1)**	**39546·5 (32959·8–46811·4)**	**301390·4 (252625·9–356004·3)**	**3901·7 (2798·6–5410·1)**	**9791·1 (6590·7–13386·5)**	**28324·9 (23500·6–33811·3)**	**84709·0 (62461·8–111261·9)**	**40109·2 (29038·2–51978·5)**	**107619·6 (65821·8–150394·0)**	**117222·3 (90781·8–148703·2)**
**Central Europe, eastern Europe, and central Asia**	**1386·0 (1169·2–1612·7)**	**10032·3 (8837·2–11397·7)**	**5517·3 (4547·2–6805·6)**	**2450·8 (2031·4–2926·8)**	**13623·9 (11471·3–16154·9)**	**161·2 (114·3–225·2)**	**415·5 (273·2–574·0)**	**1521·6 (1256·4–1827·1)**	**3600·4 (2591·3–4782·0)**	**1725·6 (1260·4–2228·7)**	**2298·6 (1075·9–3546·1)**	**7197·3 (5526·2–9089·3)**
**Central Asia**	**267·0 (213·4–325·3)**	**1884·9 (1620·4–2211·5)**	**1075·0 (827·0–1436·0)**	**490·0 (378·4–622·5)**	**2064·3 (1618·0–2586·9)**	**38·1 (26·7–54·7)**	**88·1 (58·2–121·7)**	**354·8 (291·4–426·9)**	**1003·8 (721·3–1346·4)**	**521·5 (374·1–679·5)**	**821·7 (453·6–1200·5)**	**1350·9 (1040·4–1739·8)**
Armenia	10·0 (8·0–12·1)	67·8 (57·7–79·5)	39·8 (30·8–52·7)	18·2 (14·3–22·8)	102·6 (82·3–130·5)	1·1 (0·7–1·5)	2·6 (1·7–3·7)	11·1 (9·0–13·4)	26·9 (19·3–35·4)	12·4 (8·9–16·1)	19·5 (8·9–30·0)	52·3 (40·4–66·9)
Azerbaijan	33·8 (26·9–41·2)	179·6 (151·9–214·6)	130·5 (100·5–176·5)	59·7 (46·4–75·7)	268·1 (211·1–334·2)	4·6 (3·3–6·7)	11·5 (7·5–15·8)	38·5 (31·6–46·4)	103·5 (74·4–137·2)	48·7 (35·1–63·4)	61·4 (27·2–94·9)	166·6 (128·1–214·2)
Georgia	11·6 (9·3–14·1)	104·2 (89·3–121·1)	48·3 (37·9–63·5)	21·1 (16·7–26·1)	98·3 (78·9–122·2)	1·2 (0·8–1·7)	2·9 (1·9–4·0)	13·1 (10·8–15·7)	30·9 (22·2–40·6)	14·5 (10·4–18·8)	23·1 (10·1–35·6)	65·0 (50·7–82·7)
Kazakhstan	55·8 (44·4–68·0)	436·7 (373·6–508·7)	218·8 (168·6–294·7)	96·3 (75·0–120·6)	390·3 (304·4–494·9)	8·8 (6·2–12·9)	21·4 (14·1–29·8)	68·1 (56·0–82·2)	184·7 (132·9–246·4)	93·5 (67·2–122·1)	87·1 (35·8–138·6)	278·4 (214·7–356·9)
Kyrgyzstan	16·7 (13·3–20·3)	136·1 (114·4–160·1)	70·7 (54·1–94·6)	32·8 (24·9–41·3)	130·2 (102·7–163·5)	2·1 (1·5–3·0)	4·4 (2·9–6·2)	25·1 (20·5–30·1)	73·2 (52·4–98·5)	39·2 (28·2–51·1)	96·5 (60·0–134·6)	87·8 (67·7–112·8)
Mongolia	9·8 (7·8–12·1)	93·6 (78·3–109·7)	39·1 (29·9–52·9)	17·2 (13·3–21·8)	70·9 (55·7–88·2)	1·4 (1·0–2·0)	3·4 (2·2–4·7)	12·8 (10·4–15·4)	35·3 (25·4–47·1)	17·3 (12·5–22·6)	25·5 (13·0–37·9)	49·0 (37·7–63·3)
Tajikistan	23·2 (18·3–28·8)	137·4 (115·6–163·9)	97·5 (73·8–132·0)	46·4 (35·1–59·1)	216·2 (168·3–275·1)	3·0 (2·1–4·4)	6·4 (4·1–8·9)	37·2 (30·7–45·0)	112·1 (80·0–151·3)	62·3 (44·7–81·3)	146·4 (89·2–203·7)	119·2 (91·6–153·9)
Turkmenistan	14·5 (11·5–17·8)	93·7 (79·1–111·1)	57·1 (43·9–76·2)	25·8 (20·2–32·9)	117·1 (92·2–147·1)	2·4 (1·7–3·4)	5·7 (3·8–7·9)	19·6 (16·1–23·6)	55·7 (40·0–74·7)	29·2 (21·0–38·0)	29·0 (13·5–44·8)	72·5 (55·8–93·2)
Uzbekistan	91·6 (72·9–112·4)	635·8 (537·3–751·2)	373·1 (284·6–502·0)	172·6 (132·9–220·8)	670·6 (519·2–852·1)	13·4 (9·1–19·3)	29·8 (19·6–41·5)	129·4 (105·5–156·6)	381·6 (273·6–513·2)	204·4 (146·8–267·0)	333·4 (188·6–481·7)	460·1 (354·1–592·7)
**Central Europe**	**408·1 (338·8–480·7)**	**2248·6 (1969·0–2557·9)**	**1586·5 (1296·1–2009·8)**	**726·3 (597·8–870·0)**	**4231·8 (3516·5–5124·8)**	**44·9 (32·0–62·1)**	**125·0 (82·4–172·5)**	**393·5 (325·0–472·4)**	**889·2 (640·1–1164·9)**	**400·9 (291·2–519·1)**	**474·8 (200·2–754·5)**	**2153·1 (1667·9–2716·9)**
Albania	9·0 (7·3–10·8)	44·6 (38·3–51·9)	36·0 (28·1–47·3)	16·7 (13·2–20·8)	115·9 (93·0–145·2)	1·0 (0·7–1·4)	2·5 (1·7–3·5)	9·6 (7·8–11·6)	23·8 (17·2–31·4)	11·1 (8·1–14·6)	18·2 (9·5–27·0)	48·4 (37·5–61·5)
Bosnia and Herzegovina	11·6 (9·4–14·0)	72·2 (60·7–86·3)	46·4 (36·4–60·9)	21·1 (16·5–26·1)	133·4 (107·9–166·9)	1·1 (0·7–1·5)	2·8 (1·8–3·9)	11·6 (9·5–13·9)	25·8 (18·6–33·6)	11·4 (8·2–15·0)	19·4 (9·0–30·2)	63·2 (49·2–80·5)
Bulgaria	24·6 (19·7–29·8)	155·1 (131·1–180·8)	98·4 (76·8–128·3)	37·2 (29·6–45·8)	270·3 (216·2–338·6)	2·2 (1·6–3·1)	6·4 (4·2–8·9)	23·3 (19·1–28·1)	50·2 (36·1–65·3)	21·9 (15·8–28·7)	31·6 (14·1–49·6)	137·1 (106·9–173·9)
Croatia	15·2 (12·2–18·1)	112·5 (96·5–133·1)	59·6 (46·8–77·8)	26·5 (20·9–32·8)	174·9 (141·0–216·9)	1·6 (1·1–2·2)	4·4 (2·9–6·0)	14·7 (12·0–17·8)	32·0 (23·0–41·6)	14·1 (10·1–18·5)	15·7 (5·8–25·9)	82·6 (64·5–104·7)
Czechia	38·8 (31·2–47·0)	249·5 (215·3–289·1)	148·4 (115·4–195·4)	66·0 (52·1–81·6)	376·7 (303·8–468·5)	4·3 (3·0–6·0)	12·4 (8·2–16·9)	37·3 (30·4–45·5)	79·8 (57·3–103·9)	35·5 (25·5–46·6)	33·0 (11·5–54·5)	206·1 (160·7–261·8)

Location	Schizophrenia	Major depressive disorder	Dysthymia	Bipolar disorder	Anxiety disorders	Anorexia nervosa	Bulimia nervosa	Autism spectrum disorders	Attention-deficit/hyperactivity disorder	Conduct disorder	Idiopathic developmental intellectual disability	Other mental disorders
Hungary	34·7 (28·1–41·7)	230·4 (197·8–269·7)	136·9 (107·0–179·7)	59·6 (47·2–73·7)	377·1 (302·1–468·0)	3·8 (2·7–5·3)	11·0 (7·3–15·4)	32·9 (26·7–39·9)	72·4 (52·0–94·1)	31·9 (23·0–41·9)	33·9 (12·8–55·2)	188·1 (146·7–238·7)
Montenegro	2·1 (1·7–2·5)	13·2 (11·2–15·5)	8·3 (6·5–11·0)	3·8 (3·0–4·7)	25·3 (20·4–31·8)	0·2 (0·2–0·3)	0·6 (0·4–0·9)	2·2 (1·8–2·7)	5·3 (3·8–7·0)	2·6 (1·8–3·4)	2·9 (1·2–4·7)	11·2 (8·7–14·3)
North Macedonia	7·7 (6·1–9·3)	39·3 (33·2–45·8)	29·7 (23·2–39·5)	13·6 (10·7–16·9)	86·6 (69·6–108·8)	0·8 (0·5–1·1)	2·2 (1·4–3·0)	7·8 (6·4–9·3)	18·1 (13·0–23·6)	7·8 (5·6–10·2)	11·2 (4·6–17·6)	40·1 (31·0–51·2)
Poland	140·1 (121·0–159·9)	573·6 (495·5–661·0)	531·5 (451·0–627·9)	244·5 (208·6–286·4)	1341·1 (1153·0–1545·0)	17·0 (12·1–23·7)	47·4 (31·3–66·3)	132·6 (108·5–158·8)	302·7 (218·1–403·5)	136·0 (100·3–175·4)	152·0 (61·3–243·5)	701·4 (532·2–886·9)
Romania	67·3 (54·6–80·7)	391·7 (333·9–455·4)	267·4 (208·8–351·4)	137·3 (112·6–165·0)	713·8 (572·6–897·6)	6·9 (5·0–9·6)	18·5 (12·1–25·8)	64·6 (52·8–77·2)	148·9 (107·1–194·0)	69·9 (50·3–91·7)	81·4 (34·6–129·9)	368·6 (287·4–468·0)
Serbia	29·3 (23·7–35·4)	196·4 (167·0–231·0)	118·5 (92·8–155·4)	53·0 (42·3–65·6)	327·3 (265·6–408·0)	3·0 (2·1–4·2)	8·0 (5·2–11·0)	30·6 (25·1–36·8)	72·1 (52·0–94·5)	34·2 (24·7–44·8)	49·1 (24·0–75·5)	162·1 (126·1–206·0)
Slovakia	20·1 (16·2–24·2)	112·5 (95·7–130·4)	76·2 (59·3–100·5)	34·1 (26·9–42·3)	209·8 (168·2–262·3)	2·3 (1·6–3·2)	6·7 (4·4–9·2)	19·0 (15·6–23·0)	42·8 (30·8–55·6)	18·1 (13·1–23·8)	19·7 (7·6–31·6)	103·5 (80·4–131·9)
Slovenia	7·6 (6·2–9·2)	57·7 (49·2–67·2)	29·1 (22·7–38·0)	12·9 (10·2–16·1)	79·6 (64·1–99·8)	0·8 (0·5–1·1)	2·3 (1·5–3·2)	7·3 (5·9–8·8)	15·2 (10·9–19·8)	6·5 (4·7–8·5)	6·6 (2·3–11·0)	40·8 (31·9–51·8)
**Eastern Europe**	**710·8 (613·3–815·9)**	**5898·7 (5138·1–6741·1)**	**2855·9 (2409·2–3404·2)**	**1234·5 (1043·8–1442·7)**	**7327·9 (6292·2–8503·6)**	**78·3 (55·7–108·9)**	**202·4 (131·9–282·1)**	**773·3 (638·9–927·7)**	**1707·4 (1227·3–2275·1)**	**803·2 (593·3–1035·9)**	**1002·1 (420·4–1594·7)**	**3693·3 (2810·6–4671·9)**
Belarus	32·0 (25·8–38·5)	336·2 (284·8–390·0)	131·5 (102·7–173·1)	56·3 (44·0–70·2)	361·8 (291·2–457·3)	3·1 (2·1–4·4)	7·9 (5·2–10·9)	35·1 (28·9–42·3)	74·7 (53·7–97·3)	32·6 (23·4–42·5)	52·4 (22·4–82·2)	176·4 (137·2–224·3)
Estonia	4·5 (3·6–5·5)	47·2 (40·2–55·2)	18·1 (14·2–23·6)	7·7 (6·1–9·6)	48·9 (39·7–61·5)	0·5 (0·4–0·7)	1·4 (0·9–1·9)	4·8 (4·0–5·9)	10·1 (7·3–13·2)	4·5 (3·3–5·9)	4·5 (1·6–7·5)	24·8 (19·4–31·5)
Latvia	6·5 (5·3–7·8)	70·1 (61·2–79·7)	26·6 (20·9–34·8)	11·2 (8·9–14·0)	79·8 (64·8–100·1)	0·7 (0·5–1·0)	1·8 (1·2–2·5)	6·9 (5·7–8·4)	14·1 (10·2–18·4)	6·3 (4·6–8·3)	7·2 (2·6–11·7)	36·6 (28·6–46·4)
Lithuania	9·6 (7·7–11·5)	110·0 (94·3–127·7)	39·0 (30·6–50·9)	16·6 (13·1–20·7)	133·4 (107·0–168·8)	1·1 (0·8–1·6)	2·8 (1·9–3·9)	10·3 (8·5–12·4)	20·8 (15·0–27·0)	8·9 (6·4–11·6)	9·5 (3·3–15·9)	53·3 (41·6–67·6)
Moldova	12·3 (9·8–14·9)	101·9 (86·2–120·9)	51·7 (40·3–68·6)	22·3 (17·6–27·8)	167·8 (135·6–211·7)	1·2 (0·8–1·6)	2·6 (1·7–3·7)	13·7 (11·3–16·5)	30·3 (21·8–39·6)	13·2 (9·5–17·4)	34·7 (18·6–50·3)	69·0 (53·6–88·1)
Russia	498·1 (430·1–570·5)	3580·0 (3099·7–4127·4)	1975·7 (1674·1–2325·7)	853·0 (724·6–994·0)	4999·4 (4312·0–5759·7)	58·4 (41·7–81·3)	152·0 (99·0–212·2)	541·9 (445·9–649·5)	1208·4 (871·3–1617·7)	578·3 (429·1–745·3)	594·4 (225·3–969·5)	2540·4 (1923·9–3221·2)
Ukraine	147·8 (127·2–170·0)	1653·3 (1422·2–1905·6)	613·3 (519·5–723·5)	267·3 (226·8–312·1)	1536·6 (1324·9–1780·1)	13·3 (9·3–18·9)	33·8 (21·9–47·9)	160·4 (132·4–194·0)	348·8 (251·9–465·0)	159·2 (117·5–205·6)	299·4 (150·9–455·8)	792·8 (600·4–1005·1)
**High income**	**4224·1 (3679·3–4825·6)**	**32710·7 (29240·1–36362·6)**	**12882·5 (10703·5–15641·3)**	**9277·9 (7977·1–10643·2)**	**58541·7 (49559·0–69285·3)**	**1200·3 (879·1–1629·6)**	**2907·0 (2041·8–3872·2)**	**6052·1 (5061·0–7200·3)**	**13532·2 (9950·5–17968·6)**	**4109·6 (2997·0–5352·2)**	**4023·0 (1412·6–6824·6)**	**22280·1 (17557·4–28113·6)**
**Australasia**	**127·9 (118·1–139·3)**	**1018·9 (872·9–1184·7)**	**341·7 (272·8–436·4)**	**364·3 (310·5–418·0)**	**1802·3 (1469·1–2233·5)**	**57·0 (41·9–76·8)**	**197·9 (155·9–243·0)**	**120·0 (100·2–143·7)**	**760·1 (586·5–964·5)**	**124·6 (97·6–159·0)**	**87·7 (28·3–150·0)**	**645·5 (536·9–766·5)**
Australia	108·6 (99·9–118·4)	898·4 (764·2–1052·6)	288·1 (224·8–373·4)	294·7 (250·9–337·4)	1470·7 (1175·7–1848·7)	51·0 (37·4–68·6)	180·8 (144·3–220·0)	101·4 (84·3–121·7)	667·3 (515·7–838·8)	103·1 (81·1–130·9)	72·9 (23·4–124·9)	560·9 (466·0–661·0)
New Zealand	19·3 (16·7–22·2)	120·5 (105·0–138·5)	53·6 (45·9–63·1)	69·6 (59·2–81·0)	331·6 (286·0–387·2)	5·9 (4·2–8·4)	17·1 (11·8–23·1)	18·6 (15·4–22·3)	92·8 (67·0–124·8)	21·5 (15·6–27·9)	14·8 (4·9–25·2)	84·5 (65·0–107·3)

Location	Schizophrenia	Major depressive disorder	Dysthymia	Bipolar disorder	Anxiety disorders	Anorexia nervosa	Bulimia nervosa	Autism spectrum disorders	Attention-deficit/hyperactivity disorder	Conduct disorder	Idiopathic developmental intellectual disability	Other mental disorders
**High income Asia Pacific**	**688·2 (584·4–793·6)**	**3375·1 (3033·1–3739·5)**	**1424·4 (1191·4–1705·3)**	**1300·5 (1095·9–1512·2)**	**5155·2 (4360·6–6059·0)**	**183·5 (132·9–251·4)**	**373·3 (250·8–509·4)**	**1078·2 (895·8–1288·5)**	**1745·5 (1287·1–2298·1)**	**527·3 (383·4–698·7)**	**305·1 (49·4–596·5)**	**3897·9 (3054·6–4876·1)**
Brunei	1·6 (1·2–1·9)	4·4 (3·7–5·3)	3·1 (2·3–4·1)	3·0 (2·2–3·7)	13·3 (10·4–16·8)	0·7 (0·5–1·0)	1·5 (1·0–2·1)	2·5 (2·1–3·0)	6·3 (4·6–8·8)	2·0 (1·4–2·7)	0·4 (0·0–1·0)	7·7 (5·9–10·0)
Japan	452·9 (394·7–514·0)	2369·0 (2134·1–2621·2)	926·3 (794·7–1082·4)	929·9 (801·7–1071·3)	3116·1 (2723·2–3558·0)	126·3 (92·0–171·7)	235·1 (159·7–320·6)	772·8 (641·1–924·2)	1158·3 (833·7–1540·8)	372·2 (274·2–488·7)	252·8 (42·8–482·1)	2634·6 (2047·4–3292·3)
South Korea	209·2 (169·5–251·8)	908·9 (807·7–1015·3)	455·0 (355·8–585·0)	320·4 (253·6–397·2)	1862·2 (1479·5–2342·3)	49·2 (35·2–68·9)	118·0 (77·8–161·0)	271·1 (224·3–324·2)	522·2 (386·0–690·8)	138·9 (96·7–185·3)	47·5 (6·0–105·7)	1135·3 (888·8–1447·5)
Singapore	24·5 (19·6–29·8)	92·8 (80·9–105·9)	39·9 (31·4–51·0)	47·3 (37·6–57·7)	163·6 (129·0–203·9)	7·3 (5·1–10·2)	18·6 (12·5–25·7)	31·9 (26·2–38·4)	58·7 (42·8–79·2)	14·3 (10·3–18·8)	4·3 (0·3–10·5)	120·2 (93·4–154·2)
**High income North America**	**1731·2 (1514·7–1966·7)**	**12111·6 (10795·8–13569·2)**	**4846·3 (4105·5–5712·4)**	**2433·4 (2272·5–2587·8)**	**21954·7 (18884·0–25635·7)**	**405·4 (295·1–562·4)**	**972·6 (656·0–1334·9)**	**2185·9 (1833·6–2587·4)**	**6056·0 (4390·5–8168·8)**	**1443·0 (1018·2–1909·1)**	**1485·0 (482·3–2532·8)**	**7816·2 (6074·1–9766·8)**
Canada	123·0 (121·2–124·6)	804·7 (702·1–916·3)	500·3 (388·9–653·3)	310·8 (261·1–359·8)	1710·2 (1366·2–2165·1)	34·8 (24·9–49·0)	97·9 (67·3–134·3)	206·6 (178·8–238·4)	559·2 (409·0–753·4)	116·9 (82·4–155·2)	168·2 (58·0–282·1)	807·4 (623·3–1017·0)
Greenland	0·2 (0·2–0·3)	2·8 (2·4–3·3)	0·7 (0·6–1·0)	0·4 (0·3–0·5)	2·9 (2·4–3·7)	0·1 (0·0–0·1)	0·2 (0·1–0·3)	0·3 (0·2–0·3)	1·0 (0·7–1·4)	0·2 (0·2–0·3)	0·2 (0·1–0·4)	1·2 (0·9–1·5)
USA	1607·9 (1392·5–1841·5)	11303·9 (10062·6–12641·5)	4345·1 (3708·5–5067·0)	2122·1 (1999·1–2241·9)	20241·2 (17464·7–23453·7)	370·5 (268·9–513·3)	874·6 (587·3–1198·9)	1979·0 (1648·4–2352·6)	5495·7 (3954·1–7425·9)	1325·8 (933·7–1750·5)	1316·6 (424·3–2244·0)	7007·5 (5445·4–8744·4)
**Southern Latin America**	**229·5 (185·0–277·6)**	**1546·4 (1364·5–1747·7)**	**480·6 (391·5–591·1)**	**729·4 (567·1–907·7)**	**3571·7 (3124·5–4102·7)**	**59·3 (42·1–82·5)**	**170·2 (114·9–232·9)**	**313·3 (260·3–376·4)**	**784·2 (571·3–1052·5)**	**323·8 (235·4–418·0)**	**341·7 (132·9–549·8)**	**1180·1 (913·5–1517·2)**
Argentina	151·1 (121·9–183·5)	862·7 (770·1–963·1)	304·8 (252·4–370·8)	494·1 (378·8–616·0)	2329·0 (2094·1–2589·2)	39·8 (28·2–55·4)	113·3 (75·9–154·4)	212·2 (174·3–255·7)	542·6 (393·4–725·2)	229·2 (163·7–298·2)	248·8 (102·0–394·2)	779·9 (603·3–1002·4)
Chile	66·6 (53·5–80·4)	601·2 (512·4–700·3)	148·3 (115·8–192·8)	199·0 (155·1–246·5)	1057·7 (845·5–1323·7)	16·6 (11·7–23·4)	48·4 (32·2–66·7)	85·4 (71·0–102·8)	203·8 (147·2–274·5)	78·9 (59·9–100·8)	77·2 (26·4–129·2)	337·3 (261·3–433·3)
Uruguay	11·8 (9·6–14·3)	82·3 (70·8–95·4)	27·6 (21·6–35·4)	36·2 (28·1–44·8)	184·8 (147·6–231·9)	2·9 (2·1–4·1)	8·5 (5·8–11·5)	15·7 (13·0–19·0)	37·8 (27·4–51·1)	15·7 (11·2–20·4)	15·7 (5·8–26·2)	62·9 (49·1–80·5)
**Western Europe**	**1447·3 (1226·4–1666·7)**	**14658·8 (13059·2–16521·5)**	**5789·5 (4728·4–7255·1)**	**4450·3 (3696·8–5259·9)**	**26057·8 (21710·9–31454·5)**	**495·2 (361·2–680·3)**	**1193·1 (838·0–1578·5)**	**2354·7 (1967·6–2791·1)**	**4186·4 (3081·2–5503·1)**	**1691·0 (1237·7–2174·0)**	**1803·4 (696·8–2968·1)**	**8740·4 (6888·2–11013·1)**
Andorra	0·3 (0·3–0·4)	2·6 (2·2–3·1)	1·2 (0·9–1·6)	0·9 (0·7–1·1)	4·9 (3·9–6·1)	0·1 (0·1–0·1)	0·3 (0·2–0·4)	0·5 (0·4–0·5)	0·8 (0·6–1·0)	0·3 (0·2–0·4)	0·2 (0·1–0·4)	1·8 (1·4–2·3)
Austria	30·8 (24·9–37·1)	218·7 (189·1–252·4)	121·7 (95·9–158·1)	92·9 (74·0–114·4)	559·7 (454·5–687·7)	10·0 (7·2–13·6)	33·2 (23·4–45·0)	46·9 (38·6–55·9)	102·2 (75·2–137·7)	29·8 (21·7–38·6)	26·7 (7·6–46·9)	184·7 (144·8–235·1)
Belgium	37·5 (30·4–45·0)	339·7 (294·2–391·7)	138·9 (109·7–180·2)	115·5 (91·3–142·7)	599·6 (486·5–748·4)	12·9 (9·3–17·6)	28·5 (19·9–38·3)	60·1 (49·4–72·5)	108·8 (79·5–146·5)	45·5 (33·1–59·0)	36·6 (10·7–63·2)	228·8 (179·5–291·0)
Cyprus	4·5 (3·6–5·5)	30·5 (25·9–36·1)	17·7 (13·7–23·1)	13·8 (10·8–17·3)	89·9 (72·0–112·8)	1·5 (1·1–2·1)	3·9 (2·6–5·2)	7·0 (5·8–8·5)	13·0 (9·5–17·4)	4·8 (3·5–6·2)	5·2 (1·7–8·8)	26·3 (20·4–33·7)
Denmark	16·6 (13·3–20·1)	168·4 (145·2–194·7)	76·9 (60·7–99·1)	61·5 (48·6–75·4)	296·1 (239·5–366·6)	6·7 (4·8–9·4)	16·5 (11·6–22·1)	23·4 (22·8–24·3)	39·6 (28·4–52·1)	23·7 (17·1–30·8)	4·1 (0·5–9·4)	115·9 (90·9–147·4)

Location	Schizophrenia	Major depressive disorder	Dysthymia	Bipolar disorder	Anxiety disorders	Anorexia nervosa	Bulimia nervosa	Autism spectrum disorders	Attention-deficit/hyperactivity disorder	Conduct disorder	Idiopathic developmental intellectual disability	Other mental disorders
England	164·2 (143·1–186·9)	1970·7 (1711·7–2260·7)	767·9 (653·3–898·9)	701·6 (596·2–808·8)	2637·5 (2284·3–3028·0)	62·2 (45·1–86·1)	150·8 (103·4–204·7)	415·1 (345·1–492·1)	546·5 (394·1–755·4)	253·6 (188·2–324·5)	170·0 (46·5–312·4)	1040·5 (805·1–1303·8)
Finland	17·5 (14·2–20·9)	183·9 (161·7–208·2)	83·8 (66·0–108·0)	56·0 (44·5–68·1)	229·3 (186·7–284·1)	6·8 (4·9–9·5)	14·7 (10·1–20·0)	28·5 (23·5–34·1)	64·9 (48·0–86·3)	21·4 (15·6–27·8)	22·1 (7·4–37·5)	112·9 (88·8–143·2)
France	206·3 (167·3–246·9)	2150·0 (1848·6–2481·1)	864·1 (682·9–1114·6)	663·1 (525·8–812·1)	4353·9 (3505·7–5406·1)	74·7 (53·7–101·8)	178·6 (125·1–242·0)	281·6 (240·1–328·9)	729·8 (517·7–973·5)	289·9 (210·2–376·7)	280·5 (100·7–446·7)	1299·3 (1020·2–1650·2)
Germany	276·5 (223·9–332·7)	2469·1 (2147·7–2845·1)	1088·6 (859·4–1423·7)	732·9 (616·0–860·9)	5722·5 (4633·0–6995·8)	102·8 (74·6–144·7)	187·2 (133·8–246·8)	446·1 (364·6–535·8)	349·4 (247·1–465·6)	277·8 (201·7–360·9)	215·4 (48·6–387·7)	1792·3 (1410·9–2274·8)
Greece	35·0 (28·5–41·9)	519·4 (444·3–608·8)	141·9 (112·1–185·8)	107·6 (85·0–132·1)	630·4 (515·6–785·2)	10·3 (7·4–14·2)	24·5 (16·8–33·7)	53·3 (44·1–64·6)	89·4 (65·5–118·8)	36·3 (26·3–47·3)	41·7 (14·0–71·0)	217·8 (171·4–276·1)
Iceland	1·1 (0·9–1·3)	7·1 (6·2–8·3)	4·4 (3·5–5·7)	3·5 (2·7–4·3)	18·3 (14·7–22·8)	0·4 (0·3–0·6)	1·0 (0·7–1·4)	1·9 (1·6–2·2)	4·3 (3·1–5·7)	1·6 (1·1–2·0)	1·1 (0·3–1·9)	6·6 (5·1–8·4)
Ireland	20·1 (16·2–24·4)	169·1 (150·2–189·3)	64·1 (50·0–83·5)	37·6 (31·0–44·4)	348·2 (282·2–426·2)	5·5 (4·0–7·6)	14·5 (10·1–19·5)	28·7 (23·6–34·4)	67·7 (48·1–92·0)	23·9 (17·4–30·9)	14·2 (3·5–25·8)	92·3 (72·2–118·1)
Israel	28·1 (22·4–33·8)	293·0 (254·0–341·0)	107·5 (83·6–138·3)	97·8 (76·6–120·7)	345·6 (277·2–426·2)	8·9 (6·2–12·3)	24·8 (16·8–33·8)	47·0 (39·0–56·2)	140·1 (101·2–192·1)	54·8 (40·0–70·6)	36·8 (11·7–62·0)	153·0 (118·7–196·4)
Italy	222·4 (192·8–252·0)	1946·8 (1694·8–2208·9)	796·8 (680·2–944·1)	632·7 (544·3–729·9)	3580·0 (3100·8–4100·3)	57·1 (41·9–77·6)	194·5 (138·4–259·7)	311·3 (257·2–371·6)	435·2 (312·6–589·4)	212·8 (156·3–274·4)	222·1 (77·9–368·4)	1217·7 (942·8–1527·1)
Luxembourg	2·2 (1·8–2·7)	15·5 (13·6–17·7)	8·3 (6·5–10·9)	6·4 (5·0–7·9)	34·4 (27·7–43·3)	1·0 (0·7–1·3)	2·3 (1·6–3·1)	3·4 (2·8–4·0)	6·1 (4·4–8·1)	2·3 (1·7–3·0)	1·3 (0·3–2·5)	12·5 (9·8–16·0)
Malta	1·5 (1·2–1·8)	10·7 (9·2–12·6)	6·0 (4·7–7·8)	4·6 (3·7–5·6)	29·0 (23·5–36·3)	0·5 (0·3–0·6)	1·1 (0·7–1·5)	2·4 (1·9–2·8)	3·9 (2·8–5·2)	1·4 (1·1–1·9)	1·5 (0·4–2·6)	9·2 (7·2–11·7)
Monaco	0·1 (0·1–0·2)	1·4 (1·1–1·7)	0·5 (0·4–0·7)	0·4 (0·3–0·5)	2·2 (1·7–2·7)	0·1 (0·0–0·1)	0·2 (0·1–0·2)	0·2 (0·2–0·2)	0·3 (0·2–0·4)	0·1 (0·1–0·2)	0·0 (0·0–0·1)	0·8 (0·6–1·0)
Netherlands	76·9 (62·9–90·4)	508·8 (440·6–590·3)	218·6 (171·2–281·4)	152·5 (135·6–171·4)	1236·2 (988·9–1566·0)	20·5 (14·8–27·6)	34·4 (24·0–45·6)	101·5 (82·8–121·7)	184·1 (132·9–241·0)	64·9 (46·9–84·1)	34·2 (7·7–65·0)	346·1 (271·6–440·1)
Northern Ireland	6·2 (5·4–7·2)	70·2 (60·2–81·6)	25·0 (19·7–32·5)	23·8 (20·1–27·7)	131·7 (106·0–165·4)	2·2 (1·6–3·0)	5·0 (3·5–6·9)	10·7 (8·8–13·0)	19·9 (14·5–27·1)	8·7 (6·3–11·3)	6·2 (1·6–10·8)	37·2 (29·1–47·5)
Norway	18·0 (15·6–20·4)	130·4 (112·2–150·5)	68·6 (58·5–79·8)	50·4 (43·1–58·0)	381·1 (330·6–436·0)	6·3 (4·5–8·7)	16·4 (11·3–21·9)	24·5 (20·2–29·3)	66·1 (48·4–88·0)	23·4 (17·1–29·9)	11·5 (3·0–21·1)	98·5 (76·3–123·6)
Portugal	36·1 (29·5–43·1)	466·3 (398·8–541·3)	147·9 (116·8–193·8)	107·3 (90·2–126·3)	923·6 (745·1–1140·9)	10·1 (7·2–14·0)	25·8 (17·7–35·3)	52·9 (43·4–63·4)	91·8 (67·5–122·4)	37·3 (27·0–48·7)	41·4 (14·1–68·7)	224·4 (176·6–284·7)
San Marino	0·1 (0·1–0·1)	1·2 (1·0–1·4)	0·4 (0·3–0·6)	0·3 (0·3–0·4)	1·9 (1·6–2·4)	0·0 (0·0–0·1)	0·1 (0·1–0·1)	0·2 (0·1–0·2)	0·3 (0·2–0·4)	0·1 (0·1–0·2)	0·1 (0·0–0·2)	0·7 (0·5–0·8)
Scotland	16·3 (14·6–18·0)	183·5 (157·2–215·8)	74·6 (58·9–96·2)	57·5 (45·3–70·1)	242·0 (195·1–295·2)	5·3 (3·8–7·4)	13·7 (9·5–18·4)	28·6 (23·7–34·2)	35·2 (24·9–46·8)	21·1 (15·5–27·3)	19·1 (6·0–33·0)	112·0 (87·8–142·6)
Spain	153·5 (146·0–161·1)	2053·9 (1851·2–2239·8)	645·4 (509·0–840·9)	488·0 (386·3–599·6)	2360·3 (1910·4–2918·6)	61·6 (44·5–85·1)	155·1 (106·7–211·6)	246·3 (203·2–298·5)	873·0 (644·9–1138·5)	166·9 (121·2–216·9)	523·7 (292·6–757·5)	965·1 (756·9–1225·8)
Sweden	33·4 (28·9–37·8)	352·1 (314·1–394·2)	141·5 (121·2–165·3)	110·5 (94·2–127·3)	509·0 (439·2–589·6)	10·8 (7·7–15·0)	33·5 (22·8–45·8)	67·7 (56·3–80·6)	90·7 (66·3–119·3)	44·1 (32·4–56·6)	52·1 (22·1–83·6)	190·3 (147·5–238·3)

Location	Schizophrenia	Major depressive disorder	Dysthymia	Bipolar disorder	Anxiety disorders	Anorexia nervosa	Bulimia nervosa	Autism spectrum disorders	Attention-deficit/hyperactivity disorder	Conduct disorder	Idiopathic developmental intellectual disability	Other mental disorders
Switzerland	30·7 (25·0–36·8)	269·3 (238·6–303·5)	129·9 (102·3–167·1)	95·0 (74·9–117·0)	626·9 (517·0–750·0)	13·0 (9·6–17·2)	23·8 (16·9–31·8)	47·0 (38·6–56·7)	96·8 (71·0–128·8)	30·4 (22·1–39·3)	22·4 (5·9–40·3)	181·9 (142·6–231·5)
Wales	10·2 (8·3–12·3)	113·7 (97·9–132·1)	42·3 (33·4–54·6)	32·4 (25·8–39·8)	140·8 (114·1–175·1)	3·2 (2·3–4·5)	7·6 (5·2–10·2)	16·2 (13·5–19·3)	22·7 (16·6–30·1)	12·6 (9·3–16·4)	11·8 (3·6–20·4)	64·1 (50·3–81·5)
**Latin America and Caribbean**	**1733·5 (1458·4–2034·6)**	**15784·7 (13933·3–17824·7)**	**5556·2 (4568·7–6759·6)**	**5943·3 (4906·5–7032·8)**	**33513·5 (28117·6–40260·2)**	**352·0 (249·6–499·1)**	**1076·3 (717·8–1476·9)**	**2028·9 (1671·0–2432·5)**	**10433·1 (7673·2–14065·1)**	**3138·8 (2272·7–4088·7)**	**2207·2 (840·5–3625·2)**	**8620·6 (6600·3–10960·2)**
**Andean Latin America**	**175·8 (140·5–214·5)**	**1150·8 (985·5–1340·4)**	**589·3 (448·5–774·7)**	**586·2 (448·1–740·2)**	**3510·0 (2837·6–4411·5)**	**32·7 (23·1–46·9)**	**157·9 (106·9–220·3)**	**219·2 (180·8–263·1)**	**1384·9 (1004·0–1852·4)**	**368·8 (265·0–479·6)**	**270·3 (107·1–430·8)**	**911·3 (705·2–1167·7)**
Bolivia	30·0 (24·0–36·7)	276·1 (233·7–326·2)	103·8 (78·7–136·4)	102·7 (78·6–129·6)	661·0 (528·9–845·3)	5·3 (3·7–7·5)	24·9 (16·6–34·9)	42·0 (34·4–50·8)	267·8 (194·0–360·0)	72·3 (51·9–93·9)	74·4 (36·0–113·8)	158·4 (122·3–203·5)
Ecuador	47·8 (38·3–58·2)	425·4 (362·7–499·3)	161·4 (123·0–212·1)	161·5 (124·3–205·3)	924·0 (737·8–1159·9)	9·3 (6·5–13·5)	44·5 (29·7–62·5)	60·5 (49·7–72·9)	389·9 (282·6–522·4)	106·3 (76·2–138·5)	72·8 (27·3–117·3)	248·0 (191·8–317·7)
Peru	98·0 (78·4–119·5)	449·3 (383·6–526·2)	324·1 (247·6–427·1)	321·9 (245·5–406·1)	1925·0 (1554·0–2420·9)	18·1 (12·7–26·1)	88·6 (60·6–124·3)	116·7 (95·7–140·6)	727·2 (527·5–973·1)	190·3 (136·8–247·5)	123·1 (44·4–203·7)	504·9 (391·1–647·5)
**Caribbean**	**135·5 (109·2–164·0)**	**1403·4 (1210·5–1626·4)**	**465·7 (359·9–608·3)**	**447·9 (344·5–562·4)**	**2145·7 (1724·7–2690·9)**	**22·3 (15·6–31·7)**	**70·7 (47·0–97·1)**	**160·0 (132·1–192·3)**	**1395·1 (1025·6–1871·2)**	**242·1 (175·2–313·2)**	**276·5 (129·9–427·2)**	**732·8 (567·2–936·0)**
Antigua and Barbuda	0·3 (0·2–0·4)	2·2 (1·8–2·6)	1·0 (0·7–1·3)	0·9 (0·7–1·2)	4·2 (3·4–5·3)	0·1 (0·0–0·1)	0·2 (0·1–0·3)	0·3 (0·2–0·4)	2·5 (1·8–3·3)	0·4 (0·3–0·5)	0·2 (0·1–0·4)	1·5 (1·2–1·9)
Bahamas	1·2 (1·0–1·5)	9·1 (7·6–10·8)	4·0 (3·0–5·3)	3·8 (2·9–4·7)	17·8 (13·9–22·5)	0·3 (0·2–0·4)	0·9 (0·6–1·3)	1·2 (1·0–1·5)	11·2 (8·2–14·9)	1·9 (1·4–2·5)	0·8 (0·2–1·5)	6·2 (4·8–7·9)
Barbados	1·0 (0·8–1·2)	8·3 (7·0–9·8)	3·4 (2·6–4·3)	3·1 (2·4–3·9)	14·2 (11·5–17·9)	0·1 (0·1–0·2)	0·5 (0·3–0·7)	1·0 (0·8–1·2)	7·4 (5·5–9·8)	1·2 (0·9–1·5)	0·8 (0·3–1·4)	5·4 (4·2–6·9)
Belize	1·1 (0·8–1·3)	9·6 (8·1–11·4)	3·6 (2·7–4·8)	3·8 (2·9–4·8)	17·3 (13·7–21·9)	0·2 (0·2–0·3)	0·7 (0·5–0·9)	1·4 (1·2–1·7)	14·2 (10·3–19·2)	2·8 (2·0–3·6)	2·0 (0·8–3·1)	5·5 (4·2–7·0)
Bermuda	0·2 (0·2–0·3)	1·9 (1·6–2·2)	0·8 (0·6–1·0)	0·7 (0·5–0·9)	3·1 (2·5–3·8)	0·0 (0·0–0·1)	0·1 (0·1–0·2)	0·2 (0·2–0·2)	1·4 (1·0–1·8)	0·2 (0·1–0·3)	0·1 (0·0–0·2)	1·3 (1·0–1·6)
Cuba	38·5 (31·3–46·2)	426·9 (363·0–497·3)	129·4 (101·5–168·3)	120·8 (94·1–150·9)	533·9 (431·7–673·5)	4·4 (3·1–6·3)	14·5 (9·5–19·9)	37·6 (30·6–45·6)	275·5 (203·8–365·5)	39·9 (29·0–51·6)	48·8 (21·0–78·5)	213·3 (165·9–271·3)
Dominica	0·2 (0·2–0·3)	1·7 (1·5–2·0)	0·7 (0·6–0·9)	0·7 (0·5–0·8)	3·1 (2·5–3·9)	0·0 (0·0–0·0)	0·1 (0·1–0·1)	0·2 (0·2–0·3)	2·0 (1·5–2·7)	0·3 (0·2–0·4)	0·3 (0·1–0·4)	1·1 (0·9–1·5)
Dominican Republic	30·5 (24·3–37·6)	318·0 (270·3–375·3)	101·1 (76·7–133·1)	101·3 (77·1–127·8)	492·9 (393·2–622·8)	6·4 (4·4–9·4)	20·7 (13·8–28·6)	37·5 (30·5–45·3)	344·6 (252·8–464·3)	60·7 (44·0–78·5)	34·4 (11·0–59·0)	156·5 (121·1–200·7)
Grenada	0·3 (0·3–0·4)	2·7 (2·3–3·1)	1·1 (0·8–1·4)	1·0 (0·8–1·3)	4·7 (3·7–6·0)	0·1 (0·0–0·1)	0·2 (0·1–0·3)	0·4 (0·3–0·4)	3·1 (2·3–4·2)	0·5 (0·4–0·7)	0·3 (0·1–0·6)	1·7 (1·3–2·1)
Guyana	2·0 (1·6–2·5)	32·2 (26·9–37·8)	7·2 (5·5–9·5)	7·0 (5·3–8·9)	34·4 (27·2–43·1)	0·4 (0·3–0·6)	1·2 (0·8–1·7)	2·6 (2·2–3·2)	24·9 (18·2–33·7)	4·4 (3·2–5·7)	3·7 (1·3–6·0)	11·0 (8·5–14·1)
Haiti	27·3 (21·8–33·6)	306·8 (253·0–370·7)	103·7 (77·3–137·7)	100·4 (76·1–128·8)	548·7 (431·3–692·3)	4·5 (3·1–6·5)	12·5 (8·2–17·3)	43·1 (35·3–51·7)	424·4 (307·3–576·1)	82·5 (59·8–106·8)	151·2 (82·0–220·6)	152·8 (117·7–197·1)
Jamaica	8·4 (6·7–10·2)	66·4 (56·2–78·8)	28·4 (21·8–37·3)	28·9 (22·3–36·7)	121·2 (96·9–153·2)	1·4 (1·0–2·1)	4·6 (3·0–6·4)	9·6 (7·9–11·6)	85·8 (63·2–115·7)	14·5 (10·5–18·8)	12·0 (4·3–19·7)	44·4 (34·5–56·9)
Puerto Rico	12·0 (9·7–14·6)	87·1 (74·5–102·1)	40·1 (31·6–51·2)	36·6 (28·9–45·0)	173·4 (139·1–216·2)	2·1 (1·5–3·0)	7·3 (4·9–10·0)	11·2 (9·2–13·6)	84·0 (62·3–111·6)	13·5 (9·7–17·5)	6·3 (1·6–11·2)	66·2 (51·7–84·0)

Location	Schizophrenia	Major depressive disorder	Dysthymia	Bipolar disorder	Anxiety disorders	Anorexia nervosa	Bulimia nervosa	Autism spectrum disorders	Attention-deficit/hyperactivity disorder	Conduct disorder	Idiopathic developmental intellectual disability	Other mental disorders
Saint Kitts and Nevis	0·2 (0·2–0·2)	2·1 (1·7–2·6)	0·6 (0·5–0·8)	0·6 (0·5–0·8)	2·8 (2·2–3·5)	0·0 (0·0–0·1)	0·1 (0·1–0·2)	0·2 (0·2–0·2)	1·7 (1·2–2·2)	0·3 (0·2–0·3)	0·1 (0·0–0·3)	1·0 (0·8–1·3)
Saint Lucia	0·6 (0·5–0·7)	4·9 (4·2–5·7)	1·9 (1·5–2·5)	1·8 (1·4–2·3)	8·3 (6·6–10·5)	0·1 (0·1–0·1)	0·3 (0·2–0·4)	0·6 (0·5–0·7)	4·9 (3·6–6·5)	0·8 (0·6–1·0)	0·6 (0·2–1·0)	3·0 (2·3–3·9)
Saint Vincent and the Grenadines	0·3 (0·3–0·4)	3·1 (2·7–3·7)	1·2 (0·9–1·5)	1·1 (0·9–1·4)	5·2 (4·1–6·6)	0·1 (0·0–0·1)	0·2 (0·1–0·3)	0·4 (0·3–0·5)	3·3 (2·4–4·4)	0·6 (0·4–0·7)	0·4 (0·1–0·7)	1·9 (1·4–2·4)
Suriname	1·7 (1·3–2·1)	23·3 (19·9–27·0)	5·7 (4·4–7·5)	5·4 (4·1–6·8)	24·7 (19·6–30·9)	0·3 (0·2–0·4)	1·0 (0·7–1·4)	1·9 (1·6–2·3)	17·4 (12·8–23·3)	3·1 (2·3–4·0)	1·9 (0·6–3·2)	8·9 (6·9–11·4)
Trinidad and Tobago	4·7 (3·8–5·7)	46·7 (39·8–54·7)	15·0 (11·7–19·6)	13·9 (10·8–17·3)	58·1 (46·3–73·4)	0·8 (0·6–1·2)	3·0 (2·0–4·1)	4·6 (3·8–5·6)	37·3 (27·5–49·5)	5·9 (4·3–7·6)	3·0 (0·8–5·3)	24·4 (18·8–31·2)
Virgin Islands	0·4 (0·3–0·4)	3·1 (2·6–3·6)	1·1 (0·9–1·5)	1·0 (0·8–1·2)	4·9 (3·9–6·1)	0·1 (0·0–0·1)	0·2 (0·2–0·3)	0·3 (0·3–0·4)	2·5 (1·8–3·3)	0·4 (0·3–0·6)	0·2 (0·0–0·3)	1·9 (1·4–2·4)
**Central Latin America**	**725·1 (605·2–854·8)**	**6084·0 (5299·6–6946·2)**	**2270·7 (1856·0–2787·1)**	**2224·1 (1827·4–2644·5)**	**10098·2 (8342·0–12307·6)**	**138·4 (97·0–199·0)**	**460·8 (308·0–634·7)**	**875·3 (720·1–1047·0)**	**3555·7 (2629·1–4796·2)**	**1428·0 (1045·9–1856·5)**	**880·9 (316·7–1464·4)**	**3583·1 (2755·0–4581·0)**
Colombia	144·2 (115·8–176·1)	725·9 (639·1–820·0)	430·5 (336·9–557·0)	377·5 (314·8–439·7)	2348·1 (1871·7–2963·8)	26·0 (17·9–37·1)	85·1 (57·4–117·8)	157·7 (128·0–191·0)	583·2 (431·8–768·6)	229·6 (164·1–304·1)	153·7 (51·0–260·6)	753·3 (584·0–962·3)
Costa Rica	15·0 (12·0–18·1)	122·8 (104·4–144·6)	45·3 (35·2–58·5)	44·6 (34·4–56·5)	208·8 (166·9–264·9)	2·7 (1·9–3·9)	9·3 (6·2–12·8)	15·9 (12·9–19·2)	69·3 (50·0–93·9)	22·5 (16·2–29·3)	15·9 (5·9–26·2)	75·7 (58·6–96·7)
El Salvador	16·6 (13·2–20·1)	163·5 (139·3–193·1)	54·9 (42·8–71·6)	52·7 (40·6–67·0)	279·5 (222·0–349·8)	3·3 (2·3–4·7)	10·2 (6·8–14·3)	20·6 (16·7–25·0)	99·8 (71·4–135·9)	35·6 (25·6–46·5)	29·3 (11·4–48·3)	88·3 (68·4–112·8)
Guatemala	41·8 (33·2–51·9)	446·5 (369·1–526·6)	139·8 (106·5–183·6)	138·7 (103·1–177·4)	739·8 (574·7–951·6)	9·6 (6·6–13·9)	28·9 (19·0–40·7)	60·9 (49·9–72·9)	314·8 (224·5–430·3)	119·1 (85·8–155·4)	90·3 (37·0–147·8)	219·4 (168·4–282·1)
Honduras	22·7 (18·1–28·0)	195·6 (165·8–231·6)	76·4 (58·3–100·5)	76·9 (57·8–97·5)	390·8 (307·3–495·1)	4·7 (3·2–6·9)	13·8 (9·0–19·2)	33·8 (27·5–40·7)	173·5 (123·5–236·9)	67·2 (48·4–87·6)	66·8 (30·6–102·5)	120·0 (92·3–154·5)
Mexico	372·1 (318·5–428·9)	3483·0 (3031·4–3954·2)	1171·8 (990·1–1380·2)	1197·5 (1004·3–1396·7)	4533·7 (3850·1–5302·9)	72·4 (50·3–102·9)	249·0 (166·2–341·3)	462·7 (381·5–554·1)	1408·9 (1018·4–1912·1)	743·2 (545·5–966·1)	372·3 (115·2–634·7)	1740·2 (1327·0–2208·7)
Nicaragua	17·0 (13·5–20·8)	149·8 (126·7–177·1)	53·9 (41·3–71·3)	55·6 (41·8–70·9)	283·0 (223·2–354·6)	3·0 (2·1–4·4)	9·2 (6·0–12·8)	22·5 (18·4–27·0)	111·5 (79·7–151·8)	40·9 (29·5–53·4)	39·0 (17·3–61·4)	86·7 (66·9–111·4)
Panama	12·1 (9·7–14·8)	89·1 (75·5–104·3)	36·8 (28·6–47·6)	35·9 (27·5–45·5)	153·8 (122·6–192·4)	2·7 (1·9–3·9)	9·2 (6·2–12·8)	14·2 (11·5–17·1)	65·0 (46·5–88·3)	22·9 (16·5–29·9)	10·9 (3·3–18·7)	61·7 (47·8–78·8)
Venezuela	83·6 (66·9–101·7)	707·7 (602·1–839·0)	261·4 (203·0–337·9)	244·8 (188·4–308·0)	1160·7 (919·4–1462·9)	14·0 (9·9–20·2)	46·2 (30·8–64·6)	87·1 (71·5–105·4)	729·6 (539·4–985·3)	147·0 (105·9–192·0)	102·7 (37·1–170·5)	437·9 (337·6–560·9)
**Tropical Latin America**	**697·1 (597·3–804·1)**	**7146·5 (6396·3–7937·2)**	**2230·5 (1885·1–2638·0)**	**2685·0 (2267·2–3110·9)**	**17759·6 (15154·1–20800·9)**	**158·6 (113·0–222·5)**	**387·0 (255·7–527·2)**	**774·4 (638·7–930·8)**	**4097·4 (2989·2–5623·5)**	**1099·8 (794·3–1442·2)**	**779·5 (274·7–1300·6)**	**3393·3 (2585·5–4292·5)**
Brazil	678·0 (581·1–780·9)	6950·8 (6236·6–7704·6)	2166·2 (1834·1–2559·9)	2607·4 (2209·2–3013·8)	17319·8 (14787·1–20262·8)	153·1 (109·3–214·8)	375·6 (248·1–511·6)	749·4 (618·4–901·0)	3953·7 (2880·4–5439·6)	1059·2 (765·7–1388·6)	749·6 (261·5–1252·5)	3295·5 (2510·3–4168·8)
Paraguay	19·2 (15·3–23·6)	195·7 (165·5–230·6)	64·3 (49·3–84·8)	77·6 (58·1–97·4)	439·8 (352·0–553·1)	5·5 (3·9–7·8)	11·4 (7·7–15·7)	25·0 (20·5–30·2)	143·7 (103·2–190·8)	40·6 (29·1–53·6)	29·9 (11·1–49·4)	97·8 (75·6–125·7)
**North Africa and Middle East**	**1556·7 (1270·9–1863·9)**	**20160·9 (16994·7–23842·4)**	**6858·9 (5334·3–8912·2)**	**4722·9 (3671·8–5896·4)**	**31877·0 (25646·4–39249·4)**	**327·9 (229·9–467·2)**	**1143·4 (778·2–1564·1)**	**1879·5 (1550·8–2261·6)**	**8012·9 (5852·5–10729·7)**	**3726·9 (2729·0–4800·9)**	**11576·8 (7246·0–16073·6)**	**8622·7 (6625·1–11086·2)**
Afghanistan	59·8 (47·4–74·5)	1157·6 (955·0–1399·7)	312·8 (234·8–415·1)	221·6 (164·4–290·2)	1717·1 (1305·6–2192·6)	13·1 (9·0–18·7)	34·2 (22·9–47·9)	119·2 (96·7–143·9)	464·4 (331·9–639·9)	323·0 (235·5–415·5)	1637·6 (1118·0–2164·7)	363·4 (276·0–467·6)

Location	Schizophrenia	Major depressive disorder	Dysthymia	Bipolar disorder	Anxiety disorders	Anorexia nervosa	Bulimia nervosa	Autism spectrum disorders	Attention-deficit/hyperactivity disorder	Conduct disorder	Idiopathic developmental intellectual disability	Other mental disorders
Algeria	110·8 (87·7–135·8)	1302·4 (1088·8–1553·2)	485·0 (370·0–638·6)	324·8 (249·9–412·4)	2016·0 (1593·3–2537·3)	21·3 (14·9–30·8)	73·7 (48·7–103·3)	122·4 (100·3–147·3)	413·8 (296·3–560·6)	227·5 (165·5–293·1)	665·8 (413·9–925·9)	620·9 (479·0–798·9)
Bahrain	5·4 (4·3–6·7)	60·0 (49·2–71·6)	19·3 (14·5–26·1)	13·5 (10·5–17·2)	75·7 (59·1–95·9)	0·9 (0·6–1·3)	3·9 (2·6–5·3)	4·6 (3·7–5·6)	13·6 (9·8–18·0)	5·5 (4·1–7·1)	10·9 (4·9–16·8)	28·2 (21·4–36·2)
Egypt	232·4 (185·2–287·0)	2679·3 (2245·3–3212·8)	1038·6 (788·3–1366·4)	697·8 (531·1–883·0)	4565·8 (3609·3–5799·1)	50·8 (35·2–73·7)	162·6 (110·1–225·4)	296·9 (242·1–360·0)	1093·8 (786·3–1494·9)	680·7 (496·4–876·9)	1976·1 (1184·5–2773·7)	1300·9 (1002·6–1675·0)
Iran	247·3 (209·2–287·4)	3517·5 (2911·5–4227·9)	1043·3 (883·1–1239·5)	744·0 (624·3–869·0)	6353·6 (5396·7–7499·4)	58·4 (41·0–82·3)	178·7 (119·4–245·7)	310·4 (257·0–369·8)	2167·8 (1584·2–2886·5)	414·3 (297·4–541·9)	1169·1 (699·2–1662·3)	1258·8 (956·6–1603·1)
Iraq	96·0 (75·8–119·6)	1146·8 (949·5–1358·5)	434·0 (327·3–577·3)	279·3 (210·3–359·6)	2276·8 (1786·0–2908·1)	24·5 (17·0–35·9)	76·8 (51·9–105·7)	125·4 (101·6–151·3)	501·8 (365·4–664·8)	309·0 (227·0–400·2)	672·8 (401·0–961·5)	530·5 (406·0–684·2)
Jordan	28·4 (22·6–35·2)	346·3 (288·5–420·8)	121·5 (91·9–161·0)	90·3 (67·8–114·6)	585·8 (457·1–735·3)	5·8 (4·1–8·2)	19·6 (13·2–26·8)	35·9 (29·4–43·7)	133·0 (95·6–181·5)	82·2 (60·1–106·1)	193·9 (116·0–275·7)	153·7 (118·3–198·5)
Kuwait	15·9 (12·4–19·6)	152·1 (123·1–183·9)	58·5 (43·7–79·1)	41·9 (31·8–53·4)	219·3 (170·2–280·8)	3·9 (2·7–5·4)	15·4 (10·3–21·2)	13·4 (10·9–16·3)	41·8 (30·3–56·2)	17·4 (12·7–22·5)	26·4 (11·3–41·7)	77·7 (59·7–100·6)
Lebanon	14·0 (11·2–17·1)	192·2 (160·3–227·2)	61·6 (47·3–80·2)	49·9 (40·7–59·2)	314·4 (249·5–396·6)	2·7 (1·9–3·8)	9·4 (6·3–12·9)	15·6 (12·6–18·9)	49·1 (35·2–66·6)	25·9 (19·3–32·9)	70·3 (41·5–99·8)	79·0 (61·3–101·2)
Libya	19·6 (15·7–24·1)	249·3 (208·4–300·9)	84·0 (64·0–111·4)	58·2 (44·4–74·3)	386·1 (305·1–485·7)	3·6 (2·5–5·1)	12·5 (8·4–17·4)	19·2 (15·8–23·4)	68·8 (49·3–92·4)	36·6 (26·9–47·4)	110·8 (67·7–156·1)	108·1 (83·3–139·5)
Morocco	92·6 (74·4–112·7)	1518·3 (1276·0–1808·4)	423·9 (325·7–552·3)	281·1 (215·9–354·8)	1781·6 (1416·6–2238·1)	16·9 (11·8–23·7)	54·9 (36·9–76·2)	103·4 (84·7–124·8)	362·4 (260·4–490·9)	209·7 (153·4–270·4)	670·3 (416·6–933·6)	539·1 (415·7–692·8)
Oman	14·9 (11·6–18·6)	141·6 (113·7–174·5)	53·4 (39·4–73·0)	35·6 (27·0–45·2)	219·9 (169·8–284·8)	3·2 (2·2–4·7)	13·8 (9·0–18·9)	14·6 (11·9–17·7)	51·1 (36·6–69·3)	18·9 (13·8–24·4)	40·7 (20·0–61·8)	76·7 (58·0–99·6)
Palestine	10·5 (8·3–12·9)	226·7 (191·0–270·7)	47·4 (35·8–63·0)	36·1 (27·3–45·9)	270·2 (209·7–343·9)	2·2 (1·5–3·2)	6·5 (4·4–9·0)	15·2 (12·4–18·4)	58·2 (41·8–79·7)	38·3 (27·8–49·4)	130·5 (86·5–176·1)	56·9 (43·4–73·3)
Qatar	11·4 (8·8–14·5)	101·4 (81·3–124·4)	36·1 (26·6–49·8)	26·6 (19·7–34·3)	132·8 (101·3–172·4)	2·6 (1·8–3·7)	12·5 (8·2–17·7)	10·3 (8·5–12·5)	32·7 (23·2–44·4)	8·2 (6·0–10·5)	12·7 (4·7–20·9)	56·0 (42·3–72·8)
Saudi Arabia	117·6 (92·5–146·5)	1251·8 (1025·3–1510·0)	473·5 (355·3–652·6)	317·6 (239·2–398·8)	1775·8 (1389·7–2247·4)	26·8 (18·8–37·4)	106·6 (70·5–147·3)	112·4 (91·4–136·1)	341·0 (244·8–459·5)	161·9 (119·4–209·5)	214·0 (91·2–337·0)	606·6 (465·4–786·5)
Sudan	78·4 (61·9–97·6)	1202·2 (997·9–1454·9)	381·3 (287·2–508·9)	269·8 (200·4–347·8)	1899·9 (1454·5–2429·1)	17·0 (11·7–24·7)	48·8 (33·1–67·3)	123·3 (100·1–148·3)	481·2 (345·1–658·6)	324·5 (236·3–418·8)	1329·4 (899·7–1762·8)	453·0 (344·9–582·7)
Syria	34·1 (27·4–41·7)	453·0 (378·4–541·8)	164·3 (126·5–215·8)	114·9 (87·2–145·8)	847·5 (669·3–1056·1)	6·2 (4·3–9·0)	17·1 (11·8–23·5)	41·5 (33·8–50·5)	155·2 (111·8–210·3)	108·4 (79·0–140·7)	376·6 (248·2–508·3)	201·9 (155·3–258·9)
Tunisia	33·1 (26·5–40·3)	506·3 (428·3–602·0)	145·8 (112·8–190·9)	98·2 (76·7–122·8)	616·4 (493·0–770·3)	5·4 (3·8–7·6)	18·7 (12·4–25·9)	32·9 (26·8–39·9)	106·3 (76·3–143·2)	56·9 (41·6–73·4)	186·1 (116·3–259·7)	189·9 (146·0–243·3)
Turkey	236·1 (200·9–275·0)	2617·4 (2214·9–3090·5)	1055·9 (820·2–1389·9)	729·0 (563·8–917·3)	3921·8 (3144·8–4873·7)	45·4 (32·0–64·5)	214·9 (146·6–292·7)	233·2 (190·2–283·3)	1153·1 (845·9–1511·9)	389·0 (285·1–502·9)	825·1 (419·1–1237·2)	1372·5 (1059·8–1758·0)
United Arab Emirates	39·8 (31·2–49·8)	297·7 (235·3–372·4)	127·1 (92·3–177·8)	88·0 (66·8–112·6)	417·2 (315·2–532·4)	5·6 (4·0–7·9)	30·3 (19·9–43·2)	31·9 (26·2–38·5)	35·8 (24·5–49·6)	25·7 (18·5–33·3)	57·1 (25·0–89·7)	202·6 (152·4–261·3)

Location	Schizophrenia	Major depressive disorder	Dysthymia	Bipolar disorder	Anxiety disorders	Anorexia nervosa	Bulimia nervosa	Autism spectrum disorders	Attention-deficit/hyperactivity disorder	Conduct disorder	Idiopathic developmental intellectual disability	Other mental disorders
Yemen	57·2 (45·1–70·6)	1020·8 (836·6–1229·6)	284·6 (214·3–379·2)	199·8 (150·8–257·6)	1450·9 (1115·5–1850·3)	11·1 (7·7–15·8)	31·3 (21·2–42·8)	95·8 (78·7–115·7)	279·7 (198·1–392·3)	259·4 (188·3–334·3)	1188·9 (786·6–1599·8)	337·7 (256·7–435·2)
**South Asia**	**5056·4 (4308·4–5879·5)**	**45944·9 (40077·5–52212·4)**	**21149·0 (17704·6–25103·9)**	**6506·5 (5440·6–7672·3)**	**54667·6 (46460·7–64397·2)**	**731·8 (509·7–1050·3)**	**1811·5 (1188·0–2500·7)**	**5310·5 (4355·8–6406·8)**	**11975·6 (8459·9–16399·7)**	**10802·2 (7709·1–14333·6)**	**65953·5 (45211·3–87462·5)**	**23477·2 (17917·8–29886·1)**
Bangladesh	441·7 (352·1–541·9)	5466·5 (4598·0–6455·9)	1950·2 (1503·2–2494·1)	626·9 (487·4–798·8)	5110·5 (4048·2–6433·4)	60·9 (41·8–87·9)	143·7 (94·2–199·4)	468·9 (382·5–561·1)	1349·0 (967·3–1862·0)	828·8 (587·7–1110·0)	869·5 (343·2–1394·8)	2264·2 (1749·3–2906·2)
Bhutan	2·2 (1·8–2·8)	21·1 (17·7–25·1)	9·3 (7·1–12·0)	3·1 (2·3–3·9)	25·2 (20·0–32·3)	0·4 (0·3–0·5)	0·9 (0·6–1·3)	2·3 (1·9–2·8)	5·2 (3·6–7·0)	3·7 (2·6–4·9)	10·7 (6·5–15·0)	11·0 (8·5–14·2)
India	4030·9 (3441·2–4669·5)	34539·4 (30318·1–39153·4)	16661·0 (14055·0–19746·5)	5022·9 (4233·1–5886·1)	41805·5 (35707·1–48735·4)	570·8 (401·0–815·4)	1441·2 (941·3–1997·9)	4046·2 (3320·5–4881·9)	8616·9 (6022·8–11863·4)	8152·1 (5801·9–10800·2)	58532·7 (40555·7–77111·8)	18526·9 (14154·1–23557·7)
Nepal	75·9 (60·0–93·4)	1043·3 (899·2–1211·9)	355·6 (274·1–456·4)	113·3 (87·0–144·8)	966·2 (766·6–1227·3)	11·0 (7·5–16·0)	24·1 (16·1–33·4)	89·8 (73·2–108·1)	215·8 (150·6–296·8)	174·6 (123·7–234·1)	792·5 (518·6–1069·5)	396·7 (306·7–508·8)
Pakistan	505·6 (429·4–588·7)	4874·5 (4108·9–5753·5)	2173·1 (1826·1–2558·3)	740·3 (615·8–870·8)	6760·2 (5708·5–7987·8)	88·7 (61·7–129·1)	201·5 (133·2–277·9)	703·3 (572·8–844·8)	1788·8 (1228·5–2489·3)	1643·0 (1178·8–2173·3)	5748·1 (3747·1–7849·7)	2278·3 (1740·2–2919·4)
**Southeast Asia, east Asia, and Oceania**	**7883·0 (6889·3–8938·5)**	**35064·4 (31056·2–39679·2)**	**36751·2 (30704·9–44325·8)**	**5569·0 (4678·2–6574·5)**	**75677·4 (64484·0–88209·2)**	**746·2 (518·5–1073·3)**	**1612·2 (1038·3–2260·9)**	**7236·1 (5964·3–8701·4)**	**29334·4 (21832·9–38117·5)**	**7685·3 (5484·9–10030·1)**	**11520·6 (5687·8–17526·1)**	**36358·6 (27641·4–46062·8)**
**East Asia**	**5674·0 (5042·0–6325·2)**	**26086·7 (23054·9–29571·9)**	**26618·8 (22319·8–31605·1)**	**3174·6 (2716·6–3688·8)**	**49788·3 (42817·2–57481·1)**	**485·6 (336·9–703·6)**	**1070·1 (688·1–1507·2)**	**5098·8 (4206·2–6150·0)**	**22446·1 (16905·6–28825·5)**	**3925·5 (2757·6–5142·8)**	**5376·8 (2170·0–8608·1)**	**26263·9 (19953·2–33315·8)**
China	5498·7 (4897·6–6117·0)	25335·8 (22408·5–28702·5)	25696·3 (21613·2–30509·6)	3063·5 (2623·7–3554·0)	47842·2 (41181·5–55054·7)	468·0 (324·9–677·3)	1032·9 (664·8–1456·1)	4985·8 (4111·8–6016·9)	21991·7 (16558·2–28248·1)	3776·8 (2654·3–4947·5)	4930·9 (1897·7–7980·1)	25344·1 (19228·4–32168·3)
North Korea	81·0 (65·2–98·4)	393·4 (333·0–468·8)	453·1 (350·8–592·6)	54·7 (42·8–68·9)	998·0 (799·2–1216·1)	6·1 (4·3–8·7)	11·4 (7·3–15·8)	66·0 (53·6–79·7)	298·2 (211·2–396·1)	87·5 (61·8–119·1)	383·6 (251·1–520·2)	456·3 (350·8–584·4)
Taiwan (province of China)	94·3 (74·7–114·9)	357·5 (305·7–421·2)	469·5 (362·3–621·1)	56·5 (44·8–70·5)	948·2 (760·5–1185·8)	11·5 (8·2–16·2)	25·8 (16·9–35·5)	47·0 (39·7–55·8)	156·2 (114·1–207·7)	61·1 (43·4–80·6)	62·3 (24·5–100·6)	463·5 (359·7–590·4)
**Oceania**	**32·4 (26·0–39·9)**	**204·9 (168·1–248·3)**	**152·7 (116·8–202·1)**	**31·4 (23·9–39·9)**	**496·0 (389·6–627·4)**	**4·2 (2·9–6·1)**	**7·9 (5·1–11·0)**	**40·1 (32·7–48·5)**	**169·4 (118·8–236·1)**	**83·8 (58·8–109·7)**	**175·0 (107·1–244·8)**	**160·6 (123·5–207·4)**
American Samoa	0·2 (0·1–0·2)	0·7 (0·6–0·8)	0·7 (0·6–1·0)	0·2 (0·1–0·2)	2·3 (1·8–2·8)	0·0 (0·0–0·0)	0·0 (0·0–0·1)	0·2 (0·1–0·2)	0·7 (0·5–0·9)	0·4 (0·2–0·5)	0·3 (0·1–0·4)	0·8 (0·6–1·0)
Cook Islands	0·1 (0·0–0·1)	0·3 (0·3–0·4)	0·3 (0·2–0·4)	0·1 (0·0–0·1)	0·8 (0·6–1·0)	0·0 (0·0–0·0)	0·0 (0·0–0·0)	0·1 (0·0–0·1)	0·2 (0·1–0·2)	0·1 (0·1–0·1)	0·1 (0·0–0·1)	0·3 (0·2–0·4)
Federated States of Micronesia	0·3 (0·2–0·3)	1·5 (1·3–1·9)	1·3 (1·0–1·7)	0·3 (0·2–0·3)	4·1 (3·2–5·2)	0·0 (0·0–0·0)	0·1 (0·0–0·1)	0·3 (0·2–0·4)	1·3 (0·9–1·8)	0·7 (0·5–0·9)	1·3 (0·8–1·9)	1·4 (1·0–1·7)
Fiji	2·7 (2·2–3·3)	14·1 (11·8–16·8)	12·7 (9·8–16·7)	2·5 (1·9–3·2)	36·7 (29·2–45·6)	0·3 (0·2–0·5)	0·7 (0·4–0·9)	2·7 (2·2–3·2)	10·6 (7·5–14·7)	5·0 (3·5–6·5)	6·7 (3·4–10·0)	13·1 (10·1–16·9)
Guam	0·6 (0·5–0·7)	3·2 (2·8–3·8)	2·5 (1·9–3·3)	0·5 (0·4–0·6)	7·0 (5·5–8·5)	0·1 (0·1–0·1)	0·2 (0·1–0·3)	0·5 (0·4–0·6)	1·9 (1·3–2·6)	0·9 (0·6–1·1)	0·4 (0·1–0·7)	2·6 (2·0–3·3)
Kiribati	0·3 (0·2–0·3)	1·7 (1·4–2·1)	1·4 (1·1–1·9)	0·3 (0·2–0·4)	4·6 (3·6–5·8)	0·0 (0·0–0·0)	0·1 (0·0–0·1)	0·3 (0·3–0·4)	1·5 (1·1–2·1)	0·8 (0·5–1·0)	2·0 (1·3–2·8)	1·5 (1·1–1·9)

Location	Schizophrenia	Major depressive disorder	Dysthymia	Bipolar disorder	Anxiety disorders	Anorexia nervosa	Bulimia nervosa	Autism spectrum disorders	Attention-deficit/hyperactivity disorder	Conduct disorder	Idiopathic developmental intellectual disability	Other mental disorders
Marshall Islands	0·1 (0·1–0·2)	0·8 (0·7–1·0)	0·7 (0·6–0·9)	0·1 (0·1–0·2)	2·2 (1·8–2·8)	0·0 (0·0–0·0)	0·0 (0·0–0·0)	0·2 (0·1–0·2)	0·7 (0·5–1·0)	0·4 (0·2–0·5)	0·7 (0·4–1·0)	0·8 (0·6–1·0)
Nauru	0·0 (0·0–0·0)	0·2 (0·1–0·2)	0·1 (0·1–0·2)	0·0 (0·0–0·0)	0·4 (0·3–0·5)	0·0 (0·0–0·0)	0·0 (0·0–0·0)	0·0 (0·0–0·0)	0·1 (0·1–0·2)	0·1 (0·1–0·1)	0·1 (0·0–0·1)	0·1 (0·1–0·2)
Niue	0·0 (0·0–0·0)	0·0 (0·0–0·0)	0·0 (0·0–0·0)	0·0 (0·0–0·0)	0·1 (0·1–0·1)	0·0 (0·0–0·0)	0·0 (0·0–0·0)	0·0 (0·0–0·0)	0·0 (0·0–0·0)	0·0 (0·0–0·0)	0·0 (0·0–0·0)	0·0 (0·0–0·0)
Northern Mariana Islands	0·2 (0·1–0·2)	0·7 (0·6–0·8)	0·7 (0·6–1·0)	0·1 (0·1–0·2)	1·9 (1·5–2·3)	0·0 (0·0–0·0)	0·0 (0·0–0·1)	0·1 (0·1–0·1)	0·4 (0·3–0·6)	0·2 (0·1–0·3)	0·1 (0·0–0·2)	0·7 (0·6–0·9)
Palau	0·1 (0·1–0·1)	0·4 (0·3–0·4)	0·3 (0·2–0·4)	0·1 (0·0–0·1)	0·8 (0·6–1·0)	0·0 (0·0–0·0)	0·0 (0·0–0·0)	0·1 (0·0–0·1)	0·2 (0·1–0·2)	0·1 (0·1–0·1)	0·1 (0·0–0·1)	0·3 (0·3–0·4)
Papua New Guinea	23·5 (18·8–29·1)	153·6 (125·6–186·9)	110·1 (83·9–146·4)	22·8 (17·4–29·1)	363·7 (282·4–461·8)	3·1 (2·1–4·5)	5·7 (3·7–8·0)	29·9 (24·3–36·4)	127·0 (88·9–177·1)	62·7 (43·9–81·9)	136·2 (83·6–189·3)	116·5 (89·3–150·5)
Samoa	0·5 (0·4–0·6)	2·8 (2·4–3·4)	2·5 (1·9–3·3)	0·5 (0·4–0·7)	8·3 (6·5–10·5)	0·1 (0·1–0·1)	0·1 (0·1–0·2)	0·6 (0·5–0·8)	2·8 (2·0–4·0)	1·5 (1·1–2·0)	1·9 (1·1–2·8)	2·6 (2·0–3·4)
Solomon Islands	1·4 (1·1–1·8)	9·3 (7·6–11·4)	7·1 (5·4–9·4)	1·4 (1·1–1·8)	24·2 (18·7–30·3)	0·2 (0·1–0·3)	0·3 (0·2–0·4)	2·0 (1·6–2·4)	8·7 (6·0–12·2)	4·5 (3·2–5·9)	11·6 (7·5–15·9)	7·4 (5·7–9·5)
Tokelau	0·0 (0·0–0·0)	0·0 (0·0–0·0)	0·0 (0·0–0·0)	0·0 (0·0–0·0)	0·1 (0·0–0·1)	0·0 (0·0–0·0)	0·0 (0·0–0·0)	0·0 (0·0–0·0)	0·0 (0·0–0·0)	0·0 (0·0–0·0)	0·0 (0·0–0·0)	0·0 (0·0–0·0)
Tonga	0·3 (0·2–0·3)	1·2 (1·0–1·5)	1·2 (1·0–1·6)	0·3 (0·2–0·3)	4·0 (3·2–5·0)	0·0 (0·0–0·0)	0·1 (0·0–0·1)	0·3 (0·2–0·4)	1·3 (0·9–1·8)	0·7 (0·5–0·9)	0·9 (0·5–1·3)	1·3 (1·0–1·6)
Tuvalu	0·0 (0·0–0·0)	0·2 (0·2–0·3)	0·2 (0·1–0·2)	0·0 (0·0–0·0)	0·5 (0·4–0·6)	0·0 (0·0–0·0)	0·0 (0·0–0·0)	0·0 (0·0–0·0)	0·1 (0·1–0·2)	0·1 (0·0–0·1)	0·1 (0·1–0·2)	0·2 (0·1–0·2)
Vanuatu	0·7 (0·5–0·8)	4·3 (3·5–5·2)	3·3 (2·6–4·4)	0·7 (0·5–0·9)	11·1 (8·8–14·0)	0·1 (0·1–0·1)	0·2 (0·1–0·2)	0·9 (0·7–1·1)	3·9 (2·7–5·4)	2·0 (1·4–2·6)	4·2 (2·6–5·9)	3·5 (2·7–4·5)
**Southeast Asia**	**2176·6 (1816·3–2579·1)**	**8772·8 (7591·8–10124·7)**	**9979·7 (8105·2–12448·8)**	**2363·0 (1935·1–2852·9)**	**25393·1 (21028·9–30370·5)**	**256·4 (181·2–361·8)**	**534·2 (346·9–746·7)**	**2097·1 (1729·6–2515·1)**	**6718·9 (4878·8–9093·3)**	**3676·0 (2686·0–4817·2)**	**5968·7 (3317·6–8679·0)**	**9934·1 (7611·0–12678·7)**
Cambodia	45·0 (35·9–55·3)	253·5 (213·4–305·8)	219·4 (168·7–289·4)	51·8 (39·6–66·4)	741·9 (586·0–931·5)	5·3 (3·7–7·5)	10·3 (6·7–14·5)	52·3 (42·6–63·7)	163·5 (116·9–223·1)	95·7 (68·9–124·2)	235·9 (145·3–333·3)	225·1 (173·6–289·4)
Indonesia	829·7 (706·5–959·0)	2896·4 (2452·9–3379·5)	3780·9 (3158·4–4508·2)	909·4 (765·5–1066·5)	9586·9 (8133·8–11197·8)	101·8 (71·5–144·6)	211·7 (137·7–296·7)	804·6 (662·8–963·4)	2506·3 (1808·8–3465·2)	1532·1 (1121·0–2005·1)	2347·7 (1335·7–3372·8)	3681·0 (2799·9–4676·8)
Laos	19·4 (15·4–24·1)	96·2 (79·8–115·5)	89·4 (68·7–118·2)	22·3 (17·1–28·5)	316·4 (258·0–389·1)	2·7 (1·9–3·9)	5·4 (3·5–7·4)	22·9 (18·8–27·7)	72·8 (51·8–99·4)	43·4 (31·1–56·6)	80·8 (48·1–115·3)	93·8 (72·2–120·9)
Malaysia	106·9 (85·3–132·3)	756·4 (649·3–884·4)	450·8 (346·5–593·2)	112·5 (86·3–143·7)	1489·1 (1180·0–1902·8)	16·8 (11·6–23·8)	36·9 (24·0–50·7)	101·6 (83·1–122·1)	135·2 (95·8–185·4)	159·4 (114·1–208·3)	133·0 (56·5–217·3)	477·1 (368·2–612·2)
Maldives	1·9 (1·5–2·4)	8·2 (6·9–9·8)	7·1 (5·4–9·5)	1·9 (1·4–2·4)	18·5 (14·4–23·6)	0·2 (0·1–0·3)	0·6 (0·4–0·8)	1·8 (1·4–2·1)	4·7 (3·3–6·4)	2·0 (1·4–2·5)	2·8 (1·2–4·4)	8·4 (6·4–10·9)
Mauritius	4·7 (3·8–5·7)	36·3 (30·8–42·6)	22·6 (17·4–29·7)	5·0 (4·0–6·3)	54·4 (43·3–68·2)	0·5 (0·4–0·8)	1·2 (0·8–1·6)	3·9 (3·2–4·7)	9·8 (7·1–13·3)	5·0 (3·6–6·6)	5·6 (2·2–9·0)	22·9 (17·7–29·2)
Myanmar	157·2 (126·5–192·1)	438·5 (363·8–523·0)	785·2 (604·4–1028·3)	176·9 (136·3–221·9)	2201·4 (1738·1–2750·8)	19·0 (13·4–26·8)	37·0 (23·9–51·6)	167·6 (136·8–201·4)	511·9 (367·1–696·5)	302·3 (216·0–394·8)	622·4 (362·4–884·4)	790·4 (609·4–1016·8)
Philippines	313·8 (265·9–364·0)	1525·3 (1286·5–1785·0)	1405·4 (1178·2–1664·6)	362·0 (304·6–424·5)	4932·2 (4204·2–5768·0)	41·9 (29·2–60·0)	84·7 (55·0–118·1)	363·3 (299·5–437·0)	1163·6 (837·4–1611·7)	741·0 (541·1–967·1)	1068·5 (590·8–1569·7)	1370·5 (1047·8–1748·0)
Seychelles	0·4 (0·3–0·5)	1·4 (1·2–1·7)	1·7 (1·3–2·2)	0·4 (0·3–0·5)	4·1 (3·3–5·1)	0·0 (0·0–0·1)	0·1 (0·1–0·2)	0·3 (0·3–0·4)	0·9 (0·6–1·2)	0·4 (0·3–0·6)	0·4 (0·2–0·7)	1·7 (1·3–2·2)

Location	Schizophrenia	Major depressive disorder	Dysthymia	Bipolar disorder	Anxiety disorders	Anorexia nervosa	Bulimia nervosa	Autism spectrum disorders	Attention-deficit/hyperactivity disorder	Conduct disorder	Idiopathic developmental intellectual disability	Other mental disorders
Sri Lanka	71·5 (58·1–87·0)	343·9 (296·1–399·6)	350·1 (268·5–459·1)	79·4 (62·6–99·4)	934·5 (752·2–1159·1)	8·4 (5·9–12·0)	17·3 (11·3–24·1)	68·4 (56·0–82·7)	186·8 (134·8–252·9)	107·7 (77·0–140·6)	138·1 (65·3–211·0)	351·2 (270·8–449·7)
Thailand	260·0 (209·7–316·2)	1256·4 (1078·9–1480·3)	1302·3 (997·5–1713·5)	277·0 (219·4–345·6)	2694·8 (2178·5–3398·2)	27·3 (19·2–38·9)	60·0 (38·8–83·7)	204·5 (166·2–246·5)	1109·3 (807·4–1455·6)	243·3 (173·9–318·9)	547·0 (290·7–827·3)	1309·8 (1013·5–1671·9)
Timor-Leste	2·8 (2·3–3·5)	17·0 (14·1–20·4)	14·4 (11·1–18·8)	3·6 (2·8–4·6)	46·3 (36·5–58·5)	0·5 (0·3–0·7)	0·9 (0·6–1·2)	4·4 (3·6–5·3)	14·8 (10·4–20·3)	10·0 (7·1–13·0)	15·1 (8·4–21·8)	15·0 (11·5–19·3)
Vietnam	360·4 (290·0–446·8)	1131·8 (954·3–1336·2)	1537·4 (1175·8–2024·0)	357·6 (278·5–453·0)	2339·3 (1857·1–2891·2)	31·5 (22·0–44·3)	67·4 (43·6–94·6)	298·8 (244·3–362·0)	830·6 (597·1–1125·0)	428·9 (308·6–557·4)	763·6 (429·8–1105·4)	1574·2 (1213·9–2020·3)
**Sub-Saharan Africa**	**1757·4 (1445·5–2121·4)**	**25454·7 (21786·4–29574·2)**	**11773·9 (9370·8–14911·5)**	**5076·1 (4013·6–6267·6)**	**33489·2 (27000·4–41169·2)**	**382·3 (266·3–546·6)**	**825·2 (543·9–1136·8)**	**4296·1 (3545·4–5122·6)**	**7820·2 (5451·3–10785·3)**	**8920·8 (6474·5–11488·4)**	**10039·9 (5170·3–15289·4)**	**10665·8 (8168·7–13673·9)**
**Central sub-Saharan Africa**	**205·8 (162·4–255·6)**	**4313·6 (3543·4–5197·2)**	**1372·2 (1038·0–1840·8)**	**597·2 (448·6–767·5)**	**4483·3 (3486·9–5672·6)**	**41·4 (28·8–58·5)**	**87·2 (57·5–118·7)**	**522·6 (426·2–628·3)**	**932·6 (650·2–1282·6)**	**1089·3 (800·7–1396·4)**	**1584·5 (890·5–2337·1)**	**1329·8 (1013·0–1709·8)**
Angola	47·1 (37·2–58·5)	985·1 (802·0–1184·1)	301·0 (227·5–404·5)	129·9 (97·6–166·3)	1011·0 (775·0–1288·5)	12·6 (8·6–18·1)	27·8 (18·5–37·7)	119·4 (97·0–144·2)	213·3 (147·6–294·6)	253·2 (185·7–325·0)	134·3 (42·7–231·1)	286·5 (218·1–368·9)
Central African Republic	7·8 (6·2–9·7)	194·8 (159·9–233·8)	56·6 (42·9–76·1)	23·6 (17·7–30·3)	207·1 (160·5–264·6)	1·4 (0·9–2·0)	2·7 (1·8–3·8)	20·8 (17·0–25·2)	36·8 (25·7–50·7)	42·9 (31·5–55·0)	89·9 (52·1–129·0)	54·5 (41·5–70·1)
Congo	9·9 (8·0–12·2)	192·0 (159·6–231·8)	62·3 (47·7–83·7)	25·8 (19·6–33·1)	184·4 (143·9–232·2)	2·2 (1·5–3·1)	5·1 (3·3–7·0)	20·5 (16·8–24·7)	34·9 (24·5–47·8)	39·1 (28·8–50·2)	26·1 (9·3–43·3)	62·4 (48·0–80·6)
Democratic Republic of the Congo	134·8 (105·6–168·3)	2822·7 (2308·4–3424·9)	915·2 (692·4–1226·6)	401·6 (300·0–518·4)	2963·0 (2294·0–3794·0)	23·3 (16·2–33·4)	46·6 (30·7–65·1)	349·2 (284·4–420·4)	625·2 (436·0–860·3)	729·1 (536·6–934·4)	1326·8 (762·0–1925·4)	890·0 (678·5–1144·5)
Equatorial Guinea	2·5 (2·0–3·2)	50·6 (41·1–61·8)	15·1 (11·2–20·3)	6·8 (5·0–8·9)	49·9 (38·4–64·2)	1·0 (0·7–1·5)	2·5 (1·6–3·4)	5·9 (4·9–7·1)	11·2 (7·8–15·5)	12·9 (9·6–16·6)	2·9 (0·5–5·3)	14·5 (11·0–18·7)
Gabon	3·7 (3·0–4·6)	68·3 (56·7–81·5)	22·0 (17·0–29·4)	9·4 (7·1–12·0)	67·9 (53·3–86·0)	1·0 (0·7–1·5)	2·5 (1·6–3·5)	6·7 (5·4–8·1)	11·2 (7·9–15·4)	12·2 (8·9–15·7)	4·5 (1·1–8·2)	22·0 (16·9–28·3)
**Eastern sub-Saharan Africa**	**645·6 (529·9–779·4)**	**10098·1 (8618·7–11728·8)**	**4663·6 (3667·0–5937·5)**	**2024·5 (1585·0–2499·2)**	**13386·0 (10789·4–16676·2)**	**132·7 (92·8–191·8)**	**274·4 (180·4–380·3)**	**1667·2 (1372·1–1991·5)**	**2971·9 (2058·6–4080·3)**	**3518·1 (2572·1–4518·6)**	**4791·0 (2627·8–7169·2)**	**3962·4 (3034·1–5095·8)**
Burundi	17·3 (13·6–21·5)	271·9 (227·6–323·4)	131·6 (98·0–175·1)	55·1 (41·3–70·9)	370·1 (288·2–473·9)	3·0 (2·1–4·3)	5·8 (3·8–8·1)	48·3 (39·7–58·1)	83·3 (57·9–114·6)	96·2 (69·2–123·4)	209·2 (124·6–297·7)	116·7 (88·9–150·2)
Comoros	1·4 (1·2–1·8)	18·1 (15·2–21·7)	9·8 (7·4–13·0)	4·2 (3·2–5·3)	24·8 (19·5–31·3)	0·2 (0·2–0·3)	0·5 (0·3–0·7)	2·8 (2·3–3·4)	4·6 (3·2–6·3)	4·9 (3·6–6·4)	6·1 (2·5–9·8)	9·2 (7·1–11·9)
Djibouti	2·5 (2·0–3·1)	31·0 (25·6–37·2)	16·2 (12·1–21·9)	6·9 (5·2–8·9)	37·6 (29·3–47·5)	0·4 (0·3–0·6)	1·0 (0·7–1·5)	4·9 (4·0–5·9)	7·9 (5·5–10·7)	8·0 (5·8–10·2)	7·2 (2·4–12·0)	15·4 (11·8–19·9)
Eritrea	11·3 (8·9–14·0)	172·7 (142·6–205·3)	81·7 (60·6–109·7)	34·5 (25·8–44·6)	228·2 (176·5–294·5)	2·1 (1·4–3·1)	4·5 (3·0–6·3)	27·0 (22·1–32·4)	47·7 (33·4–65·6)	53·4 (38·6–68·6)	70·1 (33·8–108·1)	72·5 (55·3–93·4)
Ethiopia	168·8 (142·4–198·6)	2468·8 (2088·8–2899·3)	1181·8 (983·1–1421·1)	543·4 (450·7–642·7)	3266·1 (2741·1–3928·5)	33·0 (23·0–47·8)	67·6 (43·9–93·8)	440·0 (361·8–527·9)	798·2 (545·7–1118·9)	974·1 (723·5–1246·9)	1261·8 (666·4–1885·2)	961·9 (735·9–1236·6)
Kenya	89·3 (75·1–104·8)	1283·1 (1117·7–1470·5)	608·6 (503·2–728·1)	269·0 (223·2–316·3)	1592·8 (1342·1–1904·2)	18·9 (13·2–27·6)	41·5 (27·2–57·4)	202·2 (166·3–242·8)	363·8 (250·8–509·5)	436·4 (324·3–559·9)	397·7 (186·7–618·1)	501·6 (382·1–644·5)

Location	Schizophrenia	Major depressive disorder	Dysthymia	Bipolar disorder	Anxiety disorders	Anorexia nervosa	Bulimia nervosa	Autism spectrum disorders	Attention-deficit/hyperactivity disorder	Conduct disorder	Idiopathic developmental intellectual disability	Other mental disorders
Madagascar	44·7 (35·6–55·1)	657·7 (547·8–778·9)	318·1 (236·2–426·9)	136·1 (101·9–173·4)	946·9 (739·1–1207·2)	8·1 (5·6–11·9)	16·9 (11·0–23·6)	107·4 (87·6–129·0)	188·0 (131·5–258·7)	212·9 (153·8–273·5)	286·0 (143·2–437·3)	282·3 (214·9–363·2)
Malawi	27·2 (21·3–33·9)	350·1 (290·6–418·6)	210·8 (155·9–280·5)	88·5 (66·1–113·9)	626·6 (485·5–804·9)	5·5 (3·8–7·9)	10·7 (7·2–14·7)	73·7 (59·9–88·7)	136·1 (94·3–187·8)	164·0 (118·4–211·4)	249·0 (129·6–364·9)	181·4 (138·4–233·0)
Mozambique	41·3 (32·7–51·8)	703·2 (584·5–833·8)	318·4 (235·9–423·4)	132·5 (99·1–171·4)	991·5 (768·3–1263·3)	8·5 (5·8–12·4)	16·6 (11·0–23·1)	118·5 (96·7–143·2)	211·1 (145·8–291·8)	249·1 (179·3–319·5)	402·8 (228·5–580·2)	271·3 (207·1–348·5)
Rwanda	22·1 (17·5–27·3)	366·7 (306·8–434·3)	159·5 (118·9–212·9)	66·6 (50·1–86·1)	428·4 (331·8–546·2)	4·2 (2·9–6·2)	9·1 (5·9–12·7)	50·1 (41·3–60·3)	87·9 (61·5–120·7)	99·3 (71·8–128·0)	128·1 (68·8–191·9)	141·5 (107·9–182·2)
Somalia	26·6 (20·7–33·3)	455·8 (377·2–547·4)	215·0 (158·5–287·4)	91·6 (67·8–117·5)	553·8 (429·1–708·2)	3·6 (2·4–5·2)	6·2 (4·1–8·8)	84·1 (68·6–101·9)	148·5 (102·7–204·7)	172·3 (124·5–220·8)	746·5 (487·1–1005·9)	187·4 (142·5–241·0)
South Sudan	15·1 (12·1–18·5)	218·5 (183·0–259·7)	102·7 (76·6–137·6)	45·1 (33·5–57·9)	391·8 (305·7–496·1)	4·4 (3·0–6·4)	9·3 (6·3–12·9)	37·7 (30·9–45·0)	67·6 (46·6–93·8)	83·0 (60·1–106·6)	26·6 (4·6–52·0)	90·1 (69·0–115·9)
Tanzania	90·3 (71·4–111·8)	1319·3 (1093·9–1570·5)	652·3 (486·0–869·1)	274·7 (205·7–348·7)	1873·2 (1453·0–2399·3)	19·8 (13·4–29·0)	41·6 (27·8–57·5)	226·3 (185·2–272·5)	392·2 (273·2–539·4)	452·8 (326·3–581·3)	465·0 (218·7–716·1)	
Uganda	57·8 (45·8–72·5)	1411·8 (1176·8–1684·2)	442·5 (328·7–585·0)	186·9 (136·4–239·5)	1405·5 (1090·5–1818·3)	13·6 (9·3–19·8)	27·2 (18·0–37·8)	169·7 (138·6–203·7)	302·8 (209·2–419·1)	360·7 (260·5–463·2)	412·2 (203·9–619·4)	371·5 (282·7–476·4)
Zambia	29·3 (23·0–36·6)	361·4 (297·1–436·0)	210·7 (155·7–280·8)	87·8 (65·2–114·6)	638·0 (493·9–814·8)	7·2 (5·0–10·5)	15·6 (10·3–21·9)	73·3 (60·3–88·3)	129·8 (90·6–178·9)	148·2 (106·9–190·5)	118·8 (46·3–194·5)	184·0 (140·3–236·7)
**Southern sub-Saharan Africa**	**172·2 (145·2–202·6)**	**2132·4 (1853·7–2419·4)**	**1036·1 (858·0–1264·3)**	**432·8 (357·3–515·0)**	**2858·3 (2403·4–3402·2)**	**36·9 (26·0–53·2)**	**95·9 (62·8–131·8)**	**298·6 (245·0–358·6)**	**486·0 (341·4–668·7)**	**517·1 (382·1–670·8)**	**365·0 (146·2–592·6)**	**1013·0 (776·4–1291·5)**
Botswana	5·3 (4·2–6·6)	63·5 (52·6–76·1)	31·3 (24·1–42·3)	12·7 (9·6–16·2)	83·3 (65·5–106·1)	1·3 (0·9–1·9)	3·5 (2·3–5·0)	9·0 (7·3–10·9)	14·7 (10·4–20·0)	15·0 (11·0–19·3)	6·8 (1·9–11·9)	31·7 (24·4–40·9)
Eswatini	2·1 (1·7–2·7)	29·6 (24·7–35·1)	13·7 (10·4–18·3)	5·6 (4·3–7·3)	37·9 (29·2–48·4)	0·6 (0·4–0·8)	1·4 (0·9–1·9)	4·4 (3·7–5·3)	7·8 (5·5–10·7)	8·6 (6·4–11·1)	4·8 (1·7–8·1)	13·3 (10·1–17·1)
Lesotho	4·0 (3·2–4·9)	79·6 (67·1–94·2)	26·7 (20·5–35·7)	10·9 (8·3–14·0)	73·5 (56·7–93·0)	0·8 (0·6–1·2)	1·9 (1·2–2·5)	8·0 (6·5–9·7)	13·8 (9·7–18·8)	14·7 (10·8–19·0)	15·5 (6·8–24·5)	26·8 (20·6–34·5)
Namibia	4·8 (3·7–5·9)	47·3 (39·7–56·0)	29·4 (22·6–39·3)	12·4 (9·3–15·8)	80·1 (62·7–101·3)	1·2 (0·9–1·8)	3·0 (2·0–4·1)	9·2 (7·5–11·2)	15·7 (11·0–21·5)	17·0 (12·5–21·9)	8·4 (2·6–14·4)	29·2 (22·5–37·6)
South Africa	131·5 (111·9–153·0)	1684·3 (1474·5–1911·0)	766·7 (645·5–907·3)	320·8 (271·5–377·5)	2150·5 (1828·4–2530·1)	27·8 (19·4–39·9)	75·2 (49·4–103·3)	209·7 (172·7–251·4)	330·6 (230·0–458·3)	341·9 (251·8–442·6)	180·7 (57·1–309·2)	750·0 (572·0–952·4)
Zimbabwe	24·5 (19·4–30·3)	228·1 (190·5–272·2)	168·3 (128·0–225·9)	70·3 (53·8–90·6)	433·0 (337·7–548·1)	5·1 (3·6–7·5)	10·9 (7·1–15·1)	58·3 (47·3–70·1)	103·5 (72·3–142·5)	119·8 (88·1–153·9)	148·7 (77·2–224·8)	162·0 (123·5–208·8)
**Western sub-Saharan Africa**	**733·8 (606·1–880·9)**	**8910·7 (7630·4–10393·6)**	**4702·1 (3772·4–5950·6)**	**2021·6 (1611·6–2476·5)**	**12761·6 (10259·0–15807·3)**	**171·2 (118·9–245·4)**	**367·7 (244·1–506·5)**	**1807·7 (1490·1–2160·1)**	**3429·7 (2375·0–4801·3)**	**3796·3 (2731·6–4932·5)**	**3299·4 (1524·6–5209·5)**	**4360·6 (3338·8–5577·4)**
Benin	19·2 (15·1–23·9)	270·7 (227·1–321·1)	126·7 (95·5–170·2)	54·1 (40·7–69·1)	364·6 (277·5–466·5)	4·2 (2·9–6·0)	8·7 (5·7–12·0)	50·5 (41·3–61·1)	92·7 (62·8–131·3)	100·6 (72·3–131·5)	113·9 (52·9–174·5)	121·1 (92·4–155·5)
Burkina Faso	33·6 (26·6–41·6)	471·8 (397·0–554·5)	228·1 (172·7–305·9)	94·8 (71·8–122·2)	627·7 (480·2–811·3)	6·9 (4·8–9·9)	14·1 (9·4–19·7)	90·0 (74·1–108·0)	164·8 (111·6–233·0)	180·9 (130·1–236·5)	230·4 (110·2–349·0)	217·2 (165·3–279·5)

Location	Schizophrenia	Major depressive disorder	Dysthymia	Bipolar disorder	Anxiety disorders	Anorexia nervosa	Bulimia nervosa	Autism spectrum disorders	Attention-deficit/hyperactivity disorder	Conduct disorder	Idiopathic developmental intellectual disability	Other mental disorders
Cabo Verde	1·3 (1·0–1·6)	19·4 (16·4–22·9)	7·7 (6·0–10·2)	3·3 (2·5–4·2)	17·7 (13·9–22·3)	0·2 (0·2–0·4)	0·6 (0·4–0·9)	2·2 (1·8–2·6)	3·6 (2·5–5·1)	3·3 (2·4–4·4)	2·1 (0·6–3·6)	8·0 (6·2–10·3)
Cameroon	48·1 (37·8–60·0)	703·8 (590·6–831·1)	313·8 (236·6–421·5)	131·4 (99·1–169·8)	874·0 (671·1–1120·4)	11·0 (7·6–16·0)	24·3 (16·3–33·2)	115·6 (94·9–139·8)	215·8 (146·6–305·4)	233·2 (167·8–305·1)	199·0 (81·2–324·5)	303·8 (231·5–390·8)
Chad	21·5 (17·1–26·7)	391·2 (325·8–464·9)	146·7 (110·9–196·1)	62·7 (47·1–81·5)	469·3 (355·6–606·0)	5·1 (3·5–7·5)	10·1 (6·8–14·0)	66·4 (53·8–79·9)	122·6 (82·8–174·4)	138·6 (99·8–181·1)	164·6 (82·1–248·9)	138·8 (105·9–178·0)
Côte d’Ivoire	45·2 (35·8–56·0)	508·4 (424·1–603·5)	286·9 (217·3–386·9)	118·9 (89·5–155·4)	788·6 (605·5–1002·3)	9·8 (6·8–14·3)	22·5 (14·9–31·0)	105·1 (86·3–126·4)	188·9 (129·3–267·0)	195·6 (141·1–255·1)	156·4 (54·1–260·5)	286·0 (217·5–369·1)
The Gambia	3·6 (2·9–4·5)	69·9 (58·4–83·8)	24·1 (18·1–32·3)	10·4 (7·7–13·5)	70·0 (53·2–90·3)	0·7 (0·5–1·1)	1·6 (1·0–2·2)	8·9 (7·2–10·7)	16·6 (11·3–23·4)	18·0 (12·9–23·6)	20·7 (9·2–32·1)	23·2 (17·7–29·7)
Ghana	59·8 (46·9–73·7)	785·5 (656·0–930·4)	379·3 (289·5–509·7)	157·5 (117·6–200·8)	920·6 (704·7–1168·8)	12·7 (8·8–18·6)	29·1 (19·2–40·8)	122·1 (100·7–147·5)	219·6 (151·0–310·2)	225·6 (161·8–296·0)	189·9 (76·9–313·0)	370·5 (283·4–477·4)
Guinea	18·9 (14·9–23·2)	271·3 (229·3–321·4)	128·2 (97·3–171·5)	53·4 (40·2–68·0)	378·4 (289·0–488·1)	3·9 (2·7–5·7)	8·0 (5·3–11·0)	49·9 (40·6–59·8)	91·9 (62·2–130·0)	101·3 (72·8–132·5)	120·7 (55·3–185·7)	122·4 (93·2–157·2)
Guinea-Bissau	3·0 (2·4–3·7)	43·6 (36·2–52·2)	20·4 (15·3–27·5)	8·4 (6·3–10·8)	54·7 (41·9–69·9)	0·6 (0·4–0·9)	1·3 (0·8–1·8)	7·5 (6·1–9·0)	14·0 (9·5–19·8)	14·9 (10·7–19·5)	18·6 (8·3–29·3)	19·4 (14·8–25·0)
Liberia	8·2 (6·5–10·1)	121·0 (100·2–143·9)	54·1 (41·2–72·8)	22·8 (17·2–29·0)	161·1 (124·8–206·1)	1·3 (0·9–2·0)	2·7 (1·8–3·8)	18·9 (15·4–22·8)	35·0 (24·0–49·5)	37·6 (27·0–49·3)	65·0 (35·0–95·0)	53·3 (40·6–68·7)
Mali	31·0 (24·6–38·2)	326·3 (271·5–392·4)	210·0 (158·9–281·2)	90·0 (67·6–116·4)	527·9 (400·4–685·7)	6·8 (4·6–10·0)	13·4 (8·9–18·6)	88·1 (71·9–105·8)	162·5 (110·1–230·2)	180·9 (130·3–236·3)	212·5 (99·4–327·4)	200·6 (153·2–257·2)
Mauritania	6·9 (5·5–8·6)	72·1 (60·5–86·4)	44·3 (34·0–59·0)	19·0 (14·5–24·0)	105·1 (80·7–133·8)	1·5 (1·1–2·2)	3·3 (2·2–4·5)	15·8 (12·9–19·1)	29·4 (19·9–41·6)	32·4 (23·2–42·4)	24·9 (9·6–40·2)	43·3 (33·1–55·6)
Niger	29·3 (23·2–36·6)	412·0 (343·9–489·2)	203·4 (154·3–272·0)	88·2 (65·3–114·1)	581·7 (439·9–749·4)	6·0 (4·1–8·7)	11·3 (7·5–15·7)	94·5 (78·0–113·8)	175·7 (118·3–249·9)	198·9 (143·3–259·8)	300·9 (154·9–450·6)	191·2 (145·6–244·9)
Nigeria	351·0 (296·7–411·5)	3744·7 (3185·3–4360·7)	2179·9 (1834·4–2608·0)	959·3 (800·3–1129·4)	5849·0 (4908·8–6975·7)	90·0 (62·3–129·6)	194·6 (129·2–267·7)	847·5 (701·4–1015·8)	1667·4 (1153·4–2322·1)	1889·1 (1353·0–2446·1)	1200·1 (489·1–1927·5)	1921·7 (1465·8–2470·1)
São Tomé and Príncipe	0·4 (0·3–0·5)	4·1 (3·4–5·0)	2·4 (1·9–3·3)	1·1 (0·8–1·4)	6·2 (4·8–8·0)	0·1 (0·1–0·1)	0·2 (0·1–0·2)	0·8 (0·7–1·0)	1·5 (1·0–2·1)	1·6 (1·1–2·1)	1·2 (0·4–1·9)	2·4 (1·9–3·1)
Senegal	25·6 (20·4–31·6)	309·8 (260·0–366·1)	165·9 (126·9–221·3)	71·2 (53·2–91·4)	413·1 (311·9–528·1)	5·3 (3·6–7·6)	11·4 (7·6–15·8)	60·2 (48·8–72·6)	111·2 (75·5–157·4)	120·0 (86·4–157·0)	100·1 (35·6–166·8)	162·5 (124·0–208·7)
Sierra Leone	13·5 (10·6–16·8)	191·9 (159·9–227·8)	90·3 (68·0–121·4)	38·0 (28·3–49·2)	284·8 (218·6–364·3)	2·6 (1·8–3·8)	5·4 (3·5–7·6)	32·7 (26·7–39·2)	59·9 (40·9–84·7)	63·7 (45·7–83·6)	96·6 (51·9–143·2)	87·7 (66·9–112·7)
Togo	13·7 (10·8–17·0)	192·9 (161·4–229·6)	89·9 (68·5–120·7)	37·1 (28·2–48·0)	266·9 (205·7–338·9)	2·4 (1·7–3·4)	5·2 (3·4–7·1)	31·0 (25·2–37·4)	56·7 (38·8–80·1)	60·0 (43·3–78·4)	82·1 (41·2–125·3)	87·4 (66·9–112·7)

Note: North Africa & Middle East and South Asia are both super-regions and regions.

eTable 8: Age-standardised prevalence per 100,000, with 95% uncertainty intervals, by mental disorder and location in 2019

Location	Schizophrenia	Major depressive disorder	Dysthymia	Bipolar disorder	Anxiety disorders	Anorexia nervosa	Bulimia nervosa	Autism spectrum disorders	Attention-deficit/hyperactivity disorder	Conduct disorder	Idiopathic developmental intellectual disability	Other mental disorders
**Global**	**287·4 (246·2–330·9)**	**2285·6 (2006·4–2591·6)**	**1228·3 (1025·5–1482·6)**	**489·8 (407·5–580·6)**	**3779·5 (3181·1–4473·3)**	**50·4 (36·1–70·1)**	**124·4 (84·0–169·9)**	**369·4 (305·9–441·2)**	**1131·9 (831·7–1494·5)**	**559·0 (405·0–722·3)**	**1426·6 (873·6–1991·7)**	**1428·7 (1108·4–1816·1)**
**Central Europe, eastern Europe, and central Asia**	**282·1 (236·0–331·1)**	**2011·2 (1758·0–2298·8)**	**1128·5 (929·0–1391·1)**	**526·7 (430·6–630·9)**	**2993·3 (2501·3–3562·5)**	**45·0 (31·9–63·4)**	**105·9 (70·8–144·9)**	**385·5 (317·8–462·4)**	**1072·8 (764·2–1453·7)**	**604·7 (440·8–780·4)**	**606·4 (286·3–930·5)**	**1401·5 (1076·5–1783·5)**
**Central Asia**	**274·7 (220·3–333·4)**	**2110·9 (1830·5–2446·6)**	**1138·0 (881·4–1514·1)**	**513·6 (401·5–647·5)**	**2221·6 (1751·5–2773·5)**	**39·3 (27·6–56·3)**	**87·9 (58·3–121·4)**	**374·8 (308·0–450·9)**	**1059·1 (758·1–1421·8)**	**584·8 (420·8–764·5)**	**861·5 (475·4–1258·4)**	**1454·7 (1127·4–1861·2)**
Armenia	282·6 (225·8–344·9)	1900·2 (1622·4–2232·7)	1139·7 (882·5–1517·2)	541·5 (419·4–683·2)	3150·6 (2522·7–3925·0)	39·4 (27·4–55·8)	87·1 (58·0–120·0)	383·1 (312·5–462·8)	1065·5 (761·2–1431·0)	590·2 (425·4–770·0)	681·7 (314·1–1045·2)	1450·8 (1124·4–1856·1)
Azerbaijan	282·7 (226·3–345·0)	1663·6 (1419·9–1964·1)	1136·9 (880·7–1513·0)	522·1 (406·7–661·8)	2477·5 (1964·7–3064·8)	44·0 (30·8–62·5)	100·6 (66·5–137·9)	379·8 (312·2–456·6)	1069·2 (764·6–1435·7)	590·2 (425·5–770·1)	602·2 (266·9–930·9)	1457·6 (1129·7–1864·3)
Georgia	277·3 (220·1–336·9)	2253·3 (1924·1–2627·4)	1137·9 (881·4–1513·9)	524·7 (406·7–657·2)	2495·0 (1996·6–3103·5)	39·7 (27·5–57·7)	88·4 (59·0–121·4)	380·8 (314·6–455·9)	1067·4 (763·9–1433·5)	589·1 (424·5–768·9)	686·3 (300·5–1058·1)	1454·3 (1127·1–1860·5)
Kazakhstan	282·4 (225·2–344·1)	2370·4 (2049·0–2759·7)	1140·3 (883·0–1516·7)	507·4 (396·7–638·2)	2115·6 (1640·3–2663·4)	49·6 (34·9–73·0)	113·9 (76·5–159·9)	371·0 (305·1–447·5)	1053·7 (753·9–1414·4)	583·0 (419·3–763·0)	476·4 (196·7–757·3)	1449·0 (1122·9–1853·7)
Kyrgyzstan	262·1 (209·7–317·1)	2313·0 (1970·3–2711·5)	1138·5 (881·7–1514·8)	515·8 (401·0–643·9)	2059·9 (1636·9–2557·4)	30·5 (21·2–43·0)	63·4 (41·6–89·0)	373·4 (306·5–449·0)	1055·5 (755·6–1416·7)	583·0 (419·3–763·0)	1421·4 (883·9–1985·4)	1454·0 (1126·8–1860·4)
Mongolia	272·5 (218·8–330·3)	2916·7 (2471·1–3394·7)	1138·6 (881·9–1515·1)	500·0 (390·1–629·6)	2142·9 (1704·7–2656·9)	41·2 (28·8–59·0)	92·6 (61·7–127·9)	370·4 (301·9–446·4)	1054·6 (755·0–1415·5)	582·9 (419·2–762·8)	733·9 (373·1–1091·1)	1453·6 (1126·5–1859·9)
Tajikistan	263·7 (209·2–321·9)	1732·6 (1488·7–2033·2)	1135·0 (879·1–1510·1)	516·0 (400·8–647·3)	2403·5 (1921·1–2994·3)	28·8 (19·9–41·2)	60·9 (39·9–84·5)	377·3 (309·9–455·1)	1059·9 (759·2–1422·3)	584·5 (420·5–764·3)	1452·7 (880·6–2021·3)	1462·9 (1133·8–1870·3)
Turkmenistan	281·0 (224·5–343·6)	1974·6 (1675·3–2304·6)	1133·2 (878·3–1506·7)	505·9 (398·9–642·8)	2341·5 (1874·0–2925·9)	46·3 (32·5–65·5)	106·7 (70·7–146·4)	378·8 (310·7–457·4)	1070·5 (768·1–1437·4)	586·3 (422·0–765·9)	556·5 (259·0–860·5)	1465·4 (1135·7–1873·0)
Uzbekistan	270·1 (217·3–327·4)	2088·0 (1793·0–2450·3)	1137·7 (881·2–1513·9)	511·3 (401·4–647·0)	2025·7 (1593·9–2576·7)	36·4 (25·0–52·3)	79·8 (53·1–110·7)	374·2 (304·7–453·0)	1058·0 (757·4–1420·0)	584·1 (420·1–764·0)	949·2 (536·1–1373·8)	1455·9 (1128·3–1862·4)
**Central Europe**	**292·0 (241·1–345·2)**	**1514·9 (1323·4–1736·9)**	**1128·5 (910·5–1435·5)**	**556·7 (449·1–675·6)**	**3276·1 (2685·6–3986·5)**	**48·8 (34·6–69·0)**	**124·9 (83·6–169·8)**	**373·6 (308·4–446·9)**	**1072·0 (764·8–1442·1)**	**598·1 (435·7–774·8)**	**460·0 (188·1–732·9)**	**1427·2 (1097·6–1821·7)**
Albania	287·5 (230·7–350·1)	1363·7 (1167·8–1593·6)	1134·5 (878·6–1510·4)	542·0 (420·3–678·5)	3837·5 (3055·2–4789·5)	38·6 (26·9–54·9)	98·8 (65·4–136·6)	370·7 (303·6–447·7)	1067·3 (764·5–1433·5)	587·4 (423·0–767·1)	716·0 (375·2–1063·7)	1463·5 (1134·3–1870·4)
Bosnia and Herzegovina	286·7 (230·6–347·0)	1674·6 (1424·9–1990·7)	1135·8 (879·6–1511·0)	548·9 (425·6–691·8)	3537·2 (2833·3–4423·2)	39·0 (27·2–56·0)	98·5 (65·3–135·6)	378·6 (308·6–451·2)	1059·4 (758·9–1421·4)	583·9 (420·0–763·8)	648·8 (303·3–1006·3)	1460·6 (1132·0–1867·7)

Location	Schizophrenia	Major depressive disorder	Dysthymia	Bipolar disorder	Anxiety disorders	Anorexia nervosa	Bulimia nervosa	Autism spectrum disorders	Attention-deficit/hyperactivity disorder	Conduct disorder	Idiopathic developmental intellectual disability	Other mental disorders
Bulgaria	288·1 (230·0–350·1)	1604·0 (1365·5–1877·7)	1134·2 (878·7–1508·2)	466·4 (363·8–583·8)	3397·4 (2709·9–4257·3)	43·7 (30·9–61·7)	113·9 (75·8–159·1)	370·3 (303·4–447·2)	1063·4 (762·4–1426·8)	584·5 (420·5–764·2)	517·7 (228·6–810·9)	1463·7 (1134·4–1871·4)
Croatia	294·1 (233·6–355·8)	1929·5 (1648·4–2235·0)	1135·0 (879·1–1509·7)	545·0 (423·9–684·2)	3595·0 (2901·6–4461·8)	47·3 (32·8–67·0)	122·9 (81·8–168·5)	378·6 (309·1–456·5)	1061·1 (760·4–1423·7)	584·2 (420·3–764·0)	409·7 (149·5–676·3)	1462·0 (1133·1–1869·4)
Czechia	296·6 (237·7–362·6)	1798·1 (1550·7–2080·5)	1133·6 (877·9–1507·4)	547·1 (419·7–690·8)	3153·9 (2534·5–3908·8)	52·8 (37·2–75·6)	140·2 (92·5–195·0)	379·7 (308·4–460·6)	1061·8 (761·4–1424·4)	584·0 (420·0–763·7)	340·7 (116·5–565·2)	1465·5 (1135·8–1873·4)
Hungary	291·5 (233·3–352·4)	1737·4 (1509·0–2019·3)	1136·5 (880·5–1511·7)	537·5 (415·4–673·7)	3406·7 (2710·3–4297·8)	49·8 (34·5–71·5)	134·5 (90·4–186·2)	373·7 (305·8–451·4)	1060·4 (759·7–1423·1)	584·2 (420·2–764·0)	388·0 (143·3–638·0)	1458·0 (1130·0–1865·0)
Montenegro	288·9 (231·5–351·4)	1734·2 (1479·2–2012·0)	1135·1 (879·0–1510·4)	540·9 (421·5–683·6)	3658·6 (2932·0–4558·8)	43·3 (29·9–61·4)	111·9 (73·8–154·7)	377·4 (308·3–458·4)	1064·7 (762·6–1429·1)	586·3 (422·0–765·9)	510·8 (214·1–815·4)	1461·7 (1132·9–1868·8)
North Macedonia	287·7 (228·2–349·4)	1481·5 (1259·7–1730·0)	1131·8 (876·4–1504·9)	541·9 (423·3–688·6)	3591·5 (2876·5–4505·7)	40·0 (27·8–56·7)	102·9 (68·6–141·4)	378·9 (310·4–456·0)	1066·2 (764·8–1430·4)	585·0 (420·9–764·7)	553·7 (231·6–872·8)	1470·1 (1139·3–1878·3)
Poland	294·5 (252·1–338·5)	1192·1 (1022·8–1385·4)	1116·6 (942·5–1302·9)	550·7 (464·7–643·8)	3082·5 (2638·3–3578·3)	53·6 (38·3–76·2)	133·2 (88·8–181·2)	373·9 (306·6–447·3)	1089·9 (777·1–1502·4)	625·4 (461·6–806·6)	432·8 (170·3–696·9)	1360·8 (1039·8–1724·5)
Romania	289·0 (229·9–351·9)	1558·6 (1319·2–1806·6)	1134·7 (879·1–1509·0)	637·2 (514·8–775·7)	3285·6 (2634·5–4093·1)	46·3 (32·2–65·7)	116·4 (76·3–160·0)	366·4 (299·7–437·8)	1062·0 (761·3–1424·9)	584·3 (420·3–764·0)	474·3 (200·5–758·5)	1462·7 (1133·6–1870·2)
Serbia	285·1 (228·3–347·5)	1731·6 (1474·2–2014·0)	1134·3 (878·4–1508·9)	540·3 (416·4–681·8)	3335·4 (2679·6–4185·3)	40·6 (28·3–57·8)	103·7 (67·7–142·8)	375·4 (307·5–451·8)	1069·4 (765·7–1435·4)	588·8 (424·2–768·4)	619·7 (305·8–953·2)	1464·0 (1134·7–1871·4)
Slovakia	295·7 (236·5–359·2)	1606·9 (1378·5–1857·8)	1135·1 (879·4–1509·6)	542·3 (422·3–687·8)	3425·7 (2743·4–4318·0)	50·0 (35·0–72·5)	133·4 (88·8–182·2)	374·6 (307·4–451·2)	1061·2 (760·6–1423·8)	584·1 (420·1–763·8)	396·3 (151·4–637·0)	1461·5 (1132·7–1869·0)
Slovenia	299·5 (239·8–365·0)	2044·9 (1758·6–2379·9)	1132·1 (876·6–1505·2)	546·8 (424·4–692·1)	3389·2 (2711·6–4201·1)	50·8 (35·9–72·7)	134·9 (89·9–187·4)	382·7 (311·5–461·9)	1064·8 (763·9–1428·4)	584·5 (420·5–764·3)	350·8 (120·8–592·4)	1468·8 (1138·3–1877·2)
**Eastern Europe**	**279·3 (238·2–323·2)**	**2259·4 (1961·0–2596·1)**	**1123·2 (945·0–1329·8)**	**516·2 (434·7–603·7)**	**3188·5 (2727·1–3719·6)**	**46·3 (32·5–65·6)**	**105·5 (69·9–145·8)**	**397·3 (328·3–476·0)**	**1084·2 (774·0–1496·1)**	**621·8 (458·7–802·8)**	**533·9 (225·4–844·9)**	**1358·5 (1038·2–1723·1)**
Belarus	277·3 (221·2–336·1)	2774·6 (2357·6–3218·2)	1139·7 (882·6–1515·6)	520·9 (404·8–655·8)	3483·2 (2799·5–4353·7)	41·2 (28·5–59·4)	93·5 (62·8–128·4)	399·6 (329·1–479·7)	1059·0 (758·2–1422·2)	584·5 (420·4–764·2)	618·0 (268·5–967·6)	1449·6 (1123·3–1854·8)
Estonia	290·1 (231·4–354·4)	2641·9 (2280·9–3076·1)	1136·4 (880·6–1510·9)	522·8 (407·3–656·7)	3378·8 (2719·2–4259·5)	53·1 (37·8–75·5)	122·8 (82·0–170·8)	404·1 (331·8–485·6)	1061·8 (761·1–1425·3)	584·1 (420·1–763·8)	386·5 (134·5–641·8)	1457·7 (1129·6–1864·7)
Latvia	286·0 (227·9–348·5)	2694·1 (2368·0–3066·8)	1138·7 (881·9–1514·1)	520·1 (403·7–662·0)	3748·0 (2999·6–4699·8)	49·9 (34·8–71·9)	116·9 (77·2–162·9)	401·0 (331·4–480·9)	1059·7 (759·0–1422·9)	583·9 (420·0–763·8)	431·4 (155·2–698·3)	1452·3 (1125·5–1858·6)
Lithuania	288·4 (229·7–349·6)	2946·7 (2537·1–3416·1)	1138·9 (882·1–1514·6)	521·2 (407·9–658·7)	4254·2 (3385·3–5371·8)	51·4 (36·0–74·0)	122·6 (81·5–168·5)	407·8 (336·7–489·8)	1058·6 (758·1–1421·3)	583·8 (419·9–763·6)	390·3 (133·8–649·2)	1451·8 (1125·0–1857·8)
Moldova	268·9 (215·0–326·7)	2146·7 (1821·4–2523·9)	1138·1 (881·3–1514·0)	518·2 (401·5–655·4)	4078·8 (3270·7–5102·9)	36·5 (25·9–52·0)	72·7 (48·5–102·1)	397·8 (327·8–480·1)	1058·8 (758·3–1421·5)	584·0 (420·0–763·8)	1043·7 (567·0–1507·8)	1454·3 (1127·0–1860·8)

Location	Schizophrenia	Major depressive disorder	Dysthymia	Bipolar disorder	Anxiety disorders	Anorexia nervosa	Bulimia nervosa	Autism spectrum disorders	Attention-deficit/hyperactivity disorder	Conduct disorder	Idiopathic developmental intellectual disability	Other mental disorders
Russia	281·8 (242·0–324·0)	2014·1 (1728·8–2333·8)	1121·4 (946·7–1307·3)	514·1 (436·0–600·7)	3133·7 (2685·8–3642·1)	49·2 (34·8–70·0)	113·1 (74·7–155·4)	396·8 (327·0–474·3)	1086·4 (774·7–1498·2)	625·3 (461·5–806·4)	451·5 (170·2–734·9)	1349·4 (1030·4–1709·2)
Ukraine	271·4 (230·4–313·9)	2866·8 (2477·5–3301·8)	1122·0 (947·1–1307·6)	521·3 (441·0–608·2)	3133·1 (2665·9–3641·2)	37·9 (26·4–54·1)	84·0 (55·6–118·4)	397·6 (327·9–481·2)	1087·5 (775·3–1499·9)	625·9 (462·0–807·3)	776·1 (396·4–1175·0)	1348·3 (1029·5–1707·7)
**High income**	**333·0 (286·4–382·8)**	**2714·6 (2402·5–3063·7)**	**1023·2 (840·7–1244·4)**	**773·5 (660·3–887·4)**	**5058·3 (4242·7–6047·4)**	**134·0 (97·9–184·5)**	**312·3 (220·3–415·8)**	**599·7 (502·2–709·4)**	**1693·1 (1235·0–2269·9)**	**588·9 (429·5–763·0)**	**404·1 (136·6–690·0)**	**1642·0 (1277·1–2080·5)**
**Australasia**	**388·5 (357·3–422·1)**	**3332·1 (2836·2–3897·9)**	**1050·0 (814·3–1350·4)**	**1182·1 (993·7–1373·2)**	**6031·9 (4885·4–7447·5)**	**226·0 (165·5–307·0)**	**747·9 (591·8–911·1)**	**436·1 (363·8–521·2)**	**3248·8 (2476·1–4108·9)**	**617·0 (484·1–785·3)**	**318·4 (100·3–548·7)**	**1858·8 (1535·2–2216·7)**
Australia	388·6 (359·8–420·4)	3439·6 (2912·0–4065·2)	1044·0 (802·6–1363·6)	1125·1 (946·8–1305·6)	5815·2 (4639·8–7271·5)	238·3 (173·7–322·6)	798·5 (637·1–969·8)	435·9 (362·9–523·7)	3377·4 (2585·8–4247·8)	612·5 (483·2–775·0)	312·9 (98·3–539·6)	1911·3 (1584·4–2256·7)
New Zealand	388·6 (332·8–448·8)	2716·8 (2336·8–3178·3)	1085·1 (917·6–1288·7)	1506·4 (1259·7–1769·8)	7263·5 (6166·7–8616·4)	156·9 (111·9–224·1)	448·8 (307·0–607·6)	437·3 (360·3–524·2)	2557·2 (1850·7–3503·5)	639·1 (466·6–830·1)	348·3 (110·6–597·9)	1556·9 (1199·0–1979·9)
**High income Asia Pacific**	**301·5 (253·8–352·5)**	**1491·3 (1326·9–1670·7)**	**618·4 (508·8–750·4)**	**601·0 (496·6–706·0)**	**2616·4 (2184·4–3108·2)**	**130·7 (94·3–182·4)**	**249·7 (169·3–340·2)**	**634·3 (528·8–756·7)**	**1453·2 (1052·4–1958·7)**	**558·6 (405·5–730·5)**	**180·2 (27·2–357·2)**	**1516·2 (1172·5–1933·5)**
Brunei	304·1 (242·7–371·8)	974·2 (826·0–1147·4)	646·0 (498·7–821·7)	606·5 (468·4–752·0)	2856·9 (2262·1–3558·2)	137·2 (98·0–193·1)	288·8 (195·3–388·6)	569·6 (469·2–686·2)	1470·9 (1052·3–2051·6)	531·9 (384·7–704·5)	93·8 (4·7–225·7)	1611·8 (1243·9–2075·1)
Japan	300·8 (258·6–345·7)	1547·2 (1375·8–1738·7)	582·0 (493·8–683·3)	644·3 (541·2–753·3)	2324·1 (1986·9–2701·7)	141·2 (102·6–195·4)	248·8 (169·4–337·8)	676·5 (562·8–805·0)	1491·1 (1057·7–2009·1)	584·0 (430·2–764·9)	228·3 (36·3–436·2)	1460·7 (1119·4–1851·2)
Singapore	319·2 (255·6–385·6)	1452·2 (1265·5–1683·4)	579·9 (451·0–740·6)	719·7 (563·4–882·8)	2708·1 (2135·1–3370·1)	140·1 (97·3–198·2)	314·9 (215·3–432·5)	588·1 (485·4–707·6)	1437·5 (1028·0–2006·6)	528·0 (381·2–700·7)	75·2 (3·3–188·2)	1608·1 (1241·2–2071·1)
South Korea	300·6 (241·0–366·4)	1420·9 (1258·7–1594·5)	695·8 (536·9–902·2)	500·3 (391·5–620·2)	3221·3 (2563·3–4047·1)	109·2 (77·4–155·5)	243·5 (163·0–331·7)	548·9 (452·7–653·7)	1372·3 (1012·7–1832·5)	502·4 (349·1–665·5)	91·5 (10·1–209·5)	1609·1 (1241·8–2072·6)
**High income North America**	**418·9 (363·8–479·2)**	**3182·1 (2807·0–3618·4)**	**1190·6 (1004·8–1419·8)**	**621·2 (579·5–663·6)**	**5559·9 (4693·5–6582·6)**	**127·9 (92·3–178·8)**	**298·3 (202·0–409·3)**	**640·0 (537·7–756·4)**	**2096·8 (1505·1–2838·7)**	**549·4 (386·7–720·7)**	**435·0 (136·7–745·1)**	**1792·5 (1372·9–2247·9)**
Canada	288·6 (285·0–292·4)	2143·7 (1841·0–2482·1)	1209·8 (922·1–1583·2)	805·3 (668·0–936·1)	4320·6 (3442·7–5477·1)	117·0 (82·8–166·7)	313·2 (216·6–427·2)	608·1 (526·8–696·6)	2037·7 (1465·3–2770·0)	500·5 (353·0–662·2)	497·6 (165·2–839·5)	1797·6 (1363·2–2267·8)
Greenland	375·7 (296·1–461·8)	4836·4 (4080·9–5675·9)	1193·4 (909·3–1560·8)	650·3 (503·6–819·1)	4856·1 (3918·6–6126·6)	136·5 (96·6–192·8)	365·1 (253·1–492·2)	499·1 (409·9–596·5)	2082·3 (1503·1–2831·6)	500·2 (357·2–664·2)	401·5 (131·6–685·0)	1833·0 (1390·5–2312·3)
USA	433·6 (372·4–500·8)	3294·1 (2911·3–3737·8)	1188·6 (1010·3–1398·4)	601·3 (565·3–638·3)	5697·7 (4843·3–6728·4)	129·1 (93·0–179·7)	296·7 (199·3–407·7)	643·4 (535·1–763·6)	2102·0 (1509·4–2853·5)	554·1 (389·2–726·4)	428·3 (133·7–736·2)	1792·2 (1372·3–2246·3)
**Southern Latin America**	**313·4 (251·9–380·9)**	**2159·8 (1903·5–2446·6)**	**662·3 (535·8–820·6)**	**1024·5 (794·6–1273·0)**	**5125·8 (4459·8–5885·1)**	**89·7 (63·6–124·7)**	**251·7 (169·3–342·8)**	**482·5 (400·8–579·0)**	**1289·0 (934·1–1738·4)**	**573·0 (416·6–741·2)**	**524·2 (198·9–847·0)**	**1590·1 (1226·8–2047·4)**
Argentina	311·5 (251·5–378·5)	1808·5 (1614·3–2022·8)	631·4 (521·2–771·4)	1039·1 (797·4–1290·2)	4983·0 (4471·6–5551·9)	87·7 (62·0–122·3)	245·4 (164·7–333·3)	480·5 (395·8–578·4)	1285·9 (928·3–1730·4)	574·5 (408·7–746·1)	561·1 (228·5–892·8)	1589·3 (1226·2–2046·3)

Location	Schizophrenia	Major depressive disorder	Dysthymia	Bipolar disorder	Anxiety disorders	Anorexia nervosa	Bulimia nervosa	Autism spectrum disorders	Attention-deficit/hyperactivity disorder	Conduct disorder	Idiopathic developmental intellectual disability	Other mental disorders
Chile	317·8 (254·4–386·1)	2993·3 (2550·9–3494·3)	723·9 (560·0–943·8)	995·2 (769·1–1240·0)	5461·9 (4357·7–6805·5)	94·4 (66·3–133·7)	265·6 (177·6–366·2)	487·6 (406·8–585·1)	1296·5 (930·1–1757·2)	568·3 (432·3–726·9)	438·3 (145·0–737·6)	1593·0 (1229·1–2051·4)
Uruguay	313·5 (251·6–380·7)	2160·9 (1849·4–2519·0)	725·5 (561·4–946·0)	984·3 (761·6–1228·3)	5153·9 (4117·7–6492·3)	91·8 (65·7–129·9)	263·0 (178·8–356·1)	481·4 (396·8–579·9)	1293·5 (927·1–1753·1)	575·8 (409·6–748·0)	477·9 (170·6–803·7)	1586·1 (1223·6–2041·9)
**Western Europe**	**272·6 (229·9–318·0)**	**2820·9 (2485·8–3196·1)**	**1111·7 (890·2–1397·5)**	**901·8 (735·7–1069·3)**	**5626·6 (4632·7–6814·1)**	**142·7 (103·7–196·4)**	**329·6 (232·9–439·1)**	**581·3 (488·2–686·4)**	**1363·5 (992·1–1824·1)**	**639·6 (468·4–822·6)**	**448·8 (168·7–746·0)**	**1556·7 (1202·2–1993·6)**
Andorra	286·5 (229·7–348·9)	2552·8 (2174·0–3010·5)	1125·6 (861·9–1471·0)	934·8 (722·0–1164·9)	5453·4 (4341·1–6872·0)	154·4 (110·5–214·4)	369·1 (254·3–502·4)	580·4 (476·1–699·5)	1296·8 (942·8–1770·3)	628·2 (457·8–812·4)	254·2 (59·5–467·2)	1611·4 (1243·7–2075·0)
Austria	276·7 (220·6–339·6)	2051·9 (1758·8–2370·1)	1127·0 (862·5–1471·8)	900·4 (697·0–1121·1)	6006·4 (4815·2–7419·0)	136·2 (97·1–186·6)	428·5 (299·6–575·5)	567·5 (468·3–675·3)	1632·4 (1187·7–2234·4)	610·0 (448·7–789·1)	324·9 (87·9–576·9)	1600·4 (1234·9–2061·5)
Belgium	274·2 (219·8–334·3)	2573·1 (2201·6–2998·0)	1036·6 (802·4–1349·3)	900·3 (690·2–1125·2)	5047·2 (4033·7–6351·2)	140·1 (100·6–192·4)	298·0 (205·5–401·2)	563·3 (464·7–677·1)	1290·1 (938·2–1762·3)	626·6 (456·4–809·6)	345·6 (95·1–602·9)	1599·1 (1233·9–2059·4)
Cyprus	274·0 (219·2–330·7)	2036·2 (1725·4–2416·2)	1128·7 (862·5–1474·9)	910·4 (702·0–1143·9)	6467·2 (5125·8–8099·4)	122·3 (86·2–169·6)	287·3 (196·4–381·8)	562·3 (465·8–672·0)	1294·8 (940·6–1770·3)	629·0 (458·6–812·5)	413·0 (130·0–703·1)	1594·7 (1230·4–2053·6)
Denmark	241·4 (189·7–294·8)	2421·9 (2079·3–2811·2)	1127·5 (862·7–1472·2)	940·3 (716·5–1181·1)	4873·9 (3836·1–6067·9)	138·9 (98·7–194·3)	334·0 (235·5–444·1)	429·0 (417·9–444·9)	918·1 (650·9–1227·8)	627·0 (456·8–810·0)	74·5 (7·9–175·3)	1602·4 (1236·5–2064·0)
England	244·2 (210·0–281·5)	3037·7 (2585·8–3511·7)	1157·4 (981·0–1354·2)	1121·4 (938·8–1309·5)	4425·2 (3796·2–5144·9)	129·7 (93·5–180·3)	299·8 (206·7–404·8)	772·9 (642·6–914·9)	1308·0 (931·1–1832·4)	685·8 (509·6–876·4)	321·5 (82·4–595·4)	1458·8 (1117·9–1849·2)
Finland	264·1 (212·6–318·8)	2961·7 (2564·4–3411·2)	1270·2 (987·3–1663·9)	890·3 (692·4–1114·4)	3993·1 (3191·7–5009·1)	157·5 (112·3–220·1)	325·8 (224·4–442·5)	557·7 (460·9–666·1)	1633·6 (1189·0–2190·1)	626·7 (456·5–809·7)	438·2 (140·6–749·7)	1603·3 (1237·2–2065·3)
France	268·3 (212·9–326·2)	2786·7 (2384·5–3246·7)	1129·6 (863·8–1474·0)	904·2 (704·5–1127·5)	6274·3 (4991·2–7816·0)	141·1 (101·3–192·1)	329·0 (230·5–444·0)	457·1 (390·0–533·8)	1500·1 (1052·0–2014·5)	626·8 (456·5–809·7)	456·9 (157·3–734·7)	1593·6 (1229·5–2052·0)
Germany	262·7 (208·3–322·0)	2392·3 (2055·8–2792·9)	1044·8 (811·3–1354·0)	746·9 (620·7–877·6)	6208·1 (4983·5–7682·8)	155·1 (112·3–217·9)	272·5 (194·8–359·5)	572·3 (467·4–687·2)	638·0 (444·7–870·7)	629·3 (458·6–812·7)	279·7 (57·1–507·8)	1606·1 (1239·4–2069·3)
Greece	272·2 (218·8–329·4)	4047·3 (3432·8–4801·6)	1129·3 (863·4–1474·0)	906·9 (703·7–1133·3)	5779·0 (4656·8–7179·5)	132·9 (94·8–186·1)	298·3 (204·3–404·3)	562·3 (466·9–679·7)	1288·5 (936·8–1760·6)	626·4 (456·3–809·4)	442·3 (140·8–758·3)	1595·7 (1231·2–2054·9)
Iceland	278·4 (225·5–341·9)	1858·0 (1590·3–2175·7)	1126·3 (862·1–1471·4)	908·4 (704·4–1132·6)	5103·8 (4080·3–6358·6)	135·6 (96·3–187·7)	320·5 (221·5–435·4)	566·1 (471·1–673·2)	1496·4 (1069·9–2016·8)	626·0 (456·0–809·0)	319·4 (80·5–568·4)	1607·6 (1240·7–2071·1)
Ireland	352·7 (281·6–431·2)	3149·4 (2775·0–3593·7)	1160·7 (892·2–1511·8)	701·3 (575·8–837·9)	6907·8 (5544·1–8441·3)	130·2 (93·0–181·7)	327·4 (225·8–438·7)	607·3 (500·5–727·2)	1642·9 (1157·3–2259·1)	626·5 (456·3–809·5)	300·1 (70·8–551·8)	1595·6 (1231·1–2054·5)
Israel	299·1 (236·5–361·1)	3079·6 (2652·7–3597·5)	1139·3 (876·5–1477·3)	1051·5 (812·3–1305·7)	3739·3 (2983·1–4614·6)	102·8 (72·3–142·3)	285·9 (194·0–391·7)	510·4 (423·9–610·0)	1593·4 (1151·5–2191·2)	627·0 (456·8–809·9)	402·0 (128·1–678·4)	1597·3 (1232·5–2057·2)
Italy	291·2 (250·9–332·3)	2562·7 (2210·4–2948·5)	1070·5 (906·4–1257·9)	893·5 (753·0–1035·7)	5562·5 (4754·8–6502·2)	129·5 (94·2–178·1)	417·3 (296·2–555·5)	567·9 (471·2–675·1)	1139·8 (806·5–1584·0)	662·7 (488·2–850·2)	408·7 (135·2–685·0)	1457·4 (1116·8–1848·2)

Location	Schizophrenia	Major depressive disorder	Dysthymia	Bipolar disorder	Anxiety disorders	Anorexia nervosa	Bulimia nervosa	Autism spectrum disorders	Attention-deficit/hyperactivity disorder	Conduct disorder	Idiopathic developmental intellectual disability	Other mental disorders
Luxembourg	281·9 (224·8–344·5)	2146·9 (1884·3–2459·0)	1127·0 (862·9–1471·5)	896·1 (690·8–1111·7)	5277·0 (4188·2–6572·1)	174·5 (125·9–238·3)	396·5 (277·3–534·9)	571·4 (470·4–687·5)	1296·0 (942·8–1770·4)	628·4 (458·1–811·9)	227·9 (52·5–425·9)	1604·6 (1238·3–2066·8)
Malta	277·1 (221·0–336·8)	2057·9 (1748·9–2426·0)	1126·4 (862·4–1471·0)	912·2 (708·3–1137·2)	6247·6 (4999·0–7869·0)	129·1 (90·7–182·0)	294·4 (202·6–402·3)	578·4 (473·9–692·3)	1297·8 (943·9–1772·6)	628·1 (457·9–811·5)	356·9 (88·9–634·1)	1605·5 (1239·0–2068·3)
Monaco	295·4 (234·0–361·0)	3015·6 (2421·1–3730·2)	1129·3 (863·1–1474·6)	900·8 (702·5–1126·3)	5501·6 (4353·5–6863·2)	247·9 (176·7–343·4)	626·8 (446·2–832·1)	548·3 (453·3–652·2)	1285·5 (933·5–1756·1)	625·6 (455·4–808·7)	98·2 (15·9–219·1)	1595·1 (1230·7–2054·0)
Netherlands	368·0 (299·9–437·4)	2524·5 (2173·9–2931·6)	1076·6 (834·2–1408·1)	787·6 (704·7–874·9)	6754·8 (5318·1–8573·7)	146·3 (104·8–198·1)	238·6 (163·8–312·9)	629·6 (515·2–754·7)	1471·5 (1054·8–1970·2)	594·3 (429·5–763·6)	213·9 (44·2–410·3)	1600·5 (1235·0–2061·4)
Northern Ireland	275·7 (236·7–317·3)	3226·1 (2747·4–3742·4)	1129·4 (863·5–1474·2)	1134·1 (941·3–1333·5)	6520·9 (5174·6–8156·3)	129·3 (92·4–182·2)	292·4 (202·1–399·1)	582·7 (479·5–706·7)	1289·0 (936·9–1761·7)	627·0 (456·8–810·0)	340·2 (83·1–596·1)	1595·5 (1231·1–2054·5)
Norway	282·7 (242·3–323·5)	2199·6 (1870·1–2564·0)	1096·7 (934·4–1283·3)	828·6 (697·1–961·0)	6735·8 (5787·0–7843·5)	135·8 (97·5–189·7)	340·7 (235·1–456·1)	484·4 (399·4–577·4)	1595·5 (1148·1–2158·2)	648·5 (475·2–826·7)	226·0 (55·1–419·5)	1465·6 (1123·4–1856·0)
Portugal	269·1 (215·7–326·3)	3371·1 (2879·3–3938·9)	1130·5 (863·8–1475·1)	873·0 (718·8–1028·6)	8027·6 (6390·2–10009·3)	123·6 (87·3–172·1)	300·4 (208·1–409·4)	547·8 (449·4–654·9)	1286·9 (935·1–1759·5)	626·6 (456·4–809·6)	430·8 (142·0–724·8)	1588·4 (1225·4–2045·1)
San Marino	280·1 (223·0–343·5)	3048·6 (2460·1–3781·6)	1133·4 (865·5–1478·4)	918·9 (708·0–1138·4)	5542·6 (4397·3–7022·9)	149·7 (105·7–206·4)	356·8 (245·4–476·6)	560·4 (457·6–669·9)	1278·9 (929·5–1749·9)	625·1 (455·2–808·2)	279·6 (67·7–510·6)	1585·1 (1222·8–2039·9)
Scotland	242·8 (215·5–269·5)	2891·4 (2459·8–3423·2)	1130·0 (863·8–1474·5)	913·9 (701·9–1137·8)	4217·4 (3350·0–5192·6)	116·8 (83·1–163·6)	289·7 (200·6–386·8)	556·0 (461·5–663·1)	890·6 (626·0–1199·9)	632·8 (466·6–815·6)	373·6 (110·5–651·3)	1593·7 (1229·6–2052·1)
Spain	265·6 (251·8–278·4)	3667·6 (3239·3–4075·5)	1155·3 (893·6–1503·4)	924·8 (712·4–1161·2)	4861·2 (3888·1–6101·9)	176·3 (125·2–241·8)	416·8 (289·2–569·8)	579·5 (479·2–701·5)	2706·0 (1957·0–3634·5)	627·5 (457·2–810·4)	1259·8 (702·7–1820·1)	1598·2 (1233·2–2058·3)
Sweden	270·3 (233·7–308·5)	3024·3 (2658·1–3419·1)	1189·3 (1010·5–1396·9)	973·9 (814·0–1137·6)	4795·1 (4113·7–5624·2)	129·0 (91·0–181·1)	387·0 (265·1–532·9)	706·8 (589·1–838·5)	1159·2 (839·8–1539·6)	662·8 (488·3–850·4)	550·3 (228·6–889·1)	1464·5 (1122·6–1855·0)
Switzerland	280·0 (224·3–339·3)	2557·4 (2265·5–2893·0)	1222·4 (941·5–1581·7)	941·7 (726·3–1175·8)	6790·6 (5513·7–8161·9)	179·6 (130·5–238·7)	312·7 (220·0–417·2)	573·4 (470·7–688·4)	1565·5 (1125·4–2121·9)	628·1 (457·7–811·3)	273·0 (67·0–498·6)	1603·5 (1237·4–2065·5)
Wales	271·0 (216·0–327·9)	3109·4 (2674·4–3626·4)	1127·4 (862·4–1472·5)	901·5 (699·6–1119·6)	4260·6 (3377·5–5365·0)	125·8 (89·4–177·7)	286·6 (199·5–387·5)	546·4 (452·6–652·5)	939·4 (672·7–1284·3)	631·8 (467·5–819·4)	396·9 (111·7–700·2)	1601·6 (1235·9–2063·0)
**Latin America and Caribbean**	**277·8 (234·0–325·5)**	**2583·8 (2293·0–2920·6)**	**900·9 (740·4–1093·2)**	**963·7 (794·2–1138·9)**	**5502·3 (4625·9–6588·7)**	**58·1 (41·0–82·2)**	**174·1 (116·4–238·9)**	**350·4 (288·8–419·7)**	**1813·3 (1327·6–2443·9)**	**573·8 (416·0–745·6)**	**381·2 (144·8–626·3)**	**1398·2 (1072·1–1777·0)**
**Andean Latin America**	**276·2 (221·3–334·9)**	**1838·1 (1584·2–2136·7)**	**933·7 (718·4–1225·8)**	**910·5 (700·6–1142·2)**	**5497·3 (4467·8–6893·1)**	**48·6 (34·5–69·5)**	**233·8 (158·5–326·9)**	**342·1 (282·4–410·5)**	**2116·8 (1537·0–2831·8)**	**571·8 (410·8–742·3)**	**419·5 (166·0–669·4)**	**1461·4 (1132·7–1868·4)**
Bolivia	266·6 (214·0–323·3)	2536·2 (2156·7–2980·8)	933·2 (718·1–1225·1)	887·1 (684·3–1112·2)	5740·5 (4619·1–7264·9)	41·8 (28·9–59·0)	196·0 (131·7–273·3)	340·8 (279·6–412·1)	2111·1 (1533·1–2825·5)	569·0 (408·3–739·0)	596·5 (289·9–913·1)	1462·7 (1133·7–1870·0)
Ecuador	275·6 (221·7–335·5)	2459·7 (2108·6–2871·8)	933·9 (718·5–1226·0)	907·6 (702·4–1150·8)	5231·1 (4193·4–6570·6)	48·8 (34·1–70·4)	236·1 (157·5–328·5)	340·5 (279·4–411·1)	2109·4 (1531·7–2822·9)	569·5 (408·7–739·5)	406·6 (152·9–655·8)	1461·0 (1132·4–1868·0)

Location	Schizophrenia	Major depressive disorder	Dysthymia	Bipolar disorder	Anxiety disorders	Anorexia nervosa	Bulimia nervosa	Autism spectrum disorders	Attention-deficit/hyperactivity disorder	Conduct disorder	Idiopathic developmental intellectual disability	Other mental disorders
Peru	279·5 (223·8–340·3)	1308·6 (1121·1–1530·3)	933·7 (718·5–1226·0)	919·4 (700·1–1157·0)	5551·8 (4493·4–7003·9)	51·0 (35·8–73·6)	245·8 (168·3–344·6)	343·4 (281·5–413·7)	2123·3 (1541·6–2839·4)	574·1 (412·9–745·4)	361·1 (129·8–598·1)	1461·3 (1132·6–1868·1)
**Caribbean**	**271·4 (218·7–329·3)**	**2816·2 (2425·5–3251·7)**	**934·3 (719·0–1226·3)**	**908·2 (695·0–1141·6)**	**4400·7 (3522·5–5499·8)**	**47·1 (33·1–67·0)**	**147·5 (98·4–203·3)**	**343·8 (283·7–413·6)**	**3064·4 (2247·0–4115·1)**	**559·3 (405·6–723·1)**	**602·9 (284·1–929·8)**	**1459·5 (1131·2–1866·5)**
Antigua and Barbuda	288·7 (230·1–352·4)	2146·1 (1805·3–2558·1)	935·4 (719·8–1227·9)	944·0 (717·6–1183·6)	4334·2 (3466·4–5420·6)	63·3 (44·8–89·2)	208·6 (140·3–290·5)	346·1 (283·1–418·2)	3062·0 (2245·6–4114·3)	560·1 (406·3–724·1)	249·5 (73·4–432·4)	1456·3 (1128·7–1862·6)
Bahamas	284·8 (227·1–345·4)	2180·2 (1852·7–2581·1)	936·2 (720·7–1228·3)	899·2 (689·9–1132·4)	4349·0 (3452·1–5467·1)	67·8 (47·1–98·5)	225·4 (152·6–310·6)	339·1 (279·5–411·5)	3040·9 (2230·0–4083·9)	556·3 (403·0–719·9)	227·8 (63·7–400·6)	1453·8 (1126·7–1859·8)
Barbados	282·6 (226·5–344·5)	2300·3 (1949·3–2718·8)	935·9 (720·5–1228·1)	932·9 (720·7–1172·2)	4328·7 (3456·6–5441·7)	56·8 (39·2–82·0)	184·4 (122·6–251·8)	346·8 (286·5–416·1)	3066·0 (2248·3–4119·0)	560·9 (407·1–724·9)	299·2 (93·9–512·3)	1454·7 (1127·4–1861·0)
Belize	270·7 (218·6–327·4)	2438·3 (2093·5–2863·4)	933·0 (717·6–1225·5)	907·5 (695·3–1141·8)	4184·1 (3330·1–5275·8)	47·8 (33·2–68·3)	149·0 (99·8–201·8)	340·6 (278·7–409·7)	3053·4 (2239·5–4099·0)	557·5 (404·1–721·2)	455·4 (178·1–733·1)	1463·9 (1134·6–1871·4)
Bermuda	305·3 (242·2–372·7)	2301·7 (1967·9–2691·0)	934·3 (719·2–1226·0)	977·4 (743·3–1240·3)	4329·2 (3459·8–5414·1)	82·9 (58·3–118·7)	285·1 (194·9–385·6)	350·4 (284·5–422·9)	3059·9 (2243·7–4107·1)	559·1 (405·5–722·8)	148·3 (30·2–276·5)	1458·7 (1130·5–1866·0)
Cuba	280·5 (225·2–340·5)	2953·2 (2526·6–3420·9)	932·4 (717·4–1224·0)	947·7 (718·2–1203·5)	4246·9 (3408·2–5368·0)	46·6 (32·8–66·9)	145·7 (96·5–202·8)	353·7 (287·2–429·1)	3098·1 (2272·0–4161·2)	562·1 (408·0–726·1)	478·9 (208·5–765·9)	1465·1 (1135·6–1872·5)
Dominica	274·8 (218·9–334·1)	2209·4 (1889·6–2613·4)	929·9 (714·8–1221·7)	901·2 (694·8–1132·7)	4279·7 (3427·6–5343·7)	49·7 (34·5–71·6)	157·1 (103·9–217·1)	347·3 (283·3–417·8)	3096·9 (2271·1–4157·2)	561·2 (407·1–725·3)	382·9 (129·5–644·6)	1471·4 (1140·1–1879·8)
Dominican Republic	277·9 (222·8–340·8)	2973·7 (2539·6–3493·9)	932·1 (717·1–1223·8)	910·1 (701·1–1144·9)	4500·4 (3591·1–5663·2)	55·0 (37·9–79·9)	175·8 (117·9–242·9)	342·0 (277·6–412·7)	3077·2 (2256·9–4130·3)	559·2 (405·5–723·0)	311·4 (99·1–534·1)	1465·8 (1136·1–1873·7)
Grenada	279·2 (222·5–338·6)	2363·2 (2030·5–2762·9)	930·3 (715·1–1221·8)	918·1 (705·6–1156·2)	4282·1 (3406·6–5394·6)	53·5 (37·6–75·4)	171·1 (115·5–235·6)	350·6 (285·5–424·0)	3114·0 (2284·2–4181·6)	563·6 (409·2–727·7)	336·3 (108·7–561·8)	1469·4 (1138·7–1877·6)
Guyana	263·0 (209·7–319·9)	4122·0 (3497·4–4790·9)	934·5 (719·2–1226·6)	869·8 (666·1–1093·1)	4381·5 (3463·8–5491·9)	46·1 (32·5–66·2)	142·1 (95·6–194·9)	338·6 (278·9–408·7)	3062·8 (2245·8–4113·3)	559·4 (405·6–723·2)	467·6 (167·1–760·8)	1458·4 (1130·3–1865·2)
Haiti	241·3 (194·0–293·9)	2770·9 (2343·7–3314·1)	937·3 (721·3–1230·1)	843·9 (652·2–1067·4)	4635·3 (3702·8–5809·1)	31·9 (22·2–45·7)	90·8 (59·7–125·6)	333·8 (273·1–400·3)	3030·3 (2222·1–4070·5)	557·5 (404·1–721·2)	1135·8 (616·0–1662·0)	1451·3 (1124·8–1856·0)
Jamaica	276·1 (221·7–336·7)	2185·4 (1852·2–2589·5)	933·5 (718·1–1225·9)	943·6 (731·6–1195·3)	4054·7 (3237·2–5107·2)	47·0 (33·0–67·9)	146·6 (98·3–202·1)	348·3 (285·8–419·2)	3069·1 (2250·6–4122·1)	560·0 (406·2–724·0)	431·7 (153·5–713·0)	1461·5 (1132·8–1868·5)
Puerto Rico	292·8 (232·5–358·7)	1992·9 (1692·2–2326·2)	936·7 (721·2–1228·9)	947·1 (727·7–1192·2)	4442·6 (3527·1–5566·5)	72·0 (50·0–103·4)	239·6 (160·6–328·6)	347·8 (284·4–418·7)	3059·2 (2243·2–4110·2)	560·6 (406·7–724·5)	199·8 (48·3–355·3)	1452·4 (1125·6–1857·9)
Saint Kitts and Nevis	284·1 (227·1–346·9)	3083·8 (2508·1–3805·5)	931·9 (717·0–1223·6)	909·9 (702·7–1146·2)	4308·9 (3475·3–5419·4)	64·2 (45·1–90·4)	224·5 (150·8–310·1)	342·1 (278·9–410·3)	3065·7 (2248·3–4113·3)	557·7 (404·3–721·4)	253·3 (71·1–441·7)	1465·4 (1135·7–1873·7)
Saint Lucia	276·9 (223·2–335·8)	2444·9 (2086·0–2848·9)	933·0 (717·7–1224·9)	924·3 (704·2–1152·7)	4307·1 (3433·9–5404·9)	52·7 (36·9–75·8)	167·1 (111·8–230·9)	345·4 (281·7–415·5)	3075·7 (2255·3–4130·0)	560·0 (406·2–724·0)	366·2 (123·7–611·7)	1462·9 (1133·8–1870·3)

Location	Schizophrenia	Major depressive disorder	Dysthymia	Bipolar disorder	Anxiety disorders	Anorexia nervosa	Bulimia nervosa	Autism spectrum disorders	Attention-deficit/hyperactivity disorder	Conduct disorder	Idiopathic developmental intellectual disability	Other mental disorders
Saint Vincent and the Grenadines	274·2 (218·3–335·5)	2499·6 (2139·9–2935·3)	930·1 (715·0–1222·2)	909·1 (696·1–1144·8)	4299·3 (3420·6–5433·8)	51·3 (35·2–73·8)	162·5 (106·8–226·4)	347·1 (281·4–417·7)	3089·2 (2265·5–4147·6)	560·7 (406·8–725·0)	370·9 (125·1–615·6)	1471·4 (1140·1–1879·6)
Suriname	275·6 (220·2–335·7)	3872·7 (3299·7–4487·4)	934·4 (719·2–1226·4)	897·3 (681·9–1132·3)	4142·0 (3283·3–5142·9)	54·3 (37·8–77·6)	174·1 (115·5–243·4)	340·7 (280·2–412·6)	3076·8 (2255·8–4132·0)	562·4 (408·3–726·4)	328·6 (109·6–559·5)	1458·3 (1130·3–1865·3)
Trinidad and Tobago	286·9 (228·7–350·3)	2973·0 (2534·3–3463·2)	931·8 (716·8–1223·5)	908·8 (691·8–1149·0)	3871·4 (3066·0–4842·3)	65·8 (45·6–94·8)	217·4 (142·8–303·0)	346·7 (283·2–420·2)	3083·7 (2261·4–4139·6)	560·2 (406·4–724·3)	230·5 (62·1–405·6)	1466·3 (1136·5–1874·2)
Virgin Islands	295·5 (236·1–359·2)	2482·9 (2119·6–2940·3)	937·8 (721·9–1230·4)	919·5 (717·3–1157·1)	4365·1 (3471·8–5491·3)	79·4 (54·6–115·0)	268·6 (181·9–367·7)	343·9 (282·9–414·6)	3046·8 (2233·5–4093·7)	560·7 (406·8–724·6)	170·1 (37·7–308·1)	1448·7 (1122·7–1852·5)
**Central Latin America**	**279·6 (234·1–328·8)**	**2376·3 (2081·4–2708·3)**	**883·7 (723·8–1077·8)**	**854·0 (703·0–1015·8)**	**3930·7 (3253·4–4782·6)**	**52·3 (36·8–75·0)**	**173·5 (116·0–239·4)**	**350·9 (288·8–419·5)**	**1403·7 (1033·9–1903·0)**	**575·9 (421·2–746·1)**	**351·4 (125·8–584·8)**	**1405·1 (1078·9–1791·6)**
Colombia	279·9 (224·8–342·2)	1412·1 (1244·9–1593·4)	836·9 (654·6–1080·8)	739·9 (615·7–863·1)	4701·7 (3765·1–5921·3)	52·6 (36·2–74·8)	169·2 (114·4–233·8)	336·2 (272·8–407·0)	1278·1 (944·6–1678·8)	539·9 (388·4–712·2)	328·1 (108·7–557·2)	1455·2 (1127·8–1861·4)
Costa Rica	286·0 (229·2–346·9)	2394·2 (2044·4–2820·2)	877·2 (680·6–1133·6)	872·7 (672·6–1110·6)	4195·2 (3337·5–5278·6)	55·4 (39·0–79·4)	182·5 (122·8–252·6)	342·5 (279·2–413·5)	1541·3 (1102·0–2090·5)	553·9 (399·3–722·1)	342·7 (126·5–566·3)	1453·4 (1126·4–1859·1)
El Salvador	268·6 (215·1–325·6)	2590·9 (2210·1–3069·5)	882·4 (685·1–1138·6)	825·9 (638·6–1047·7)	4409·8 (3536·8–5530·2)	47·5 (33·4–67·8)	151·2 (101·6–209·6)	327·9 (266·0–396·9)	1532·4 (1096·6–2076·6)	555·4 (400·4–724·1)	462·6 (178·7–764·1)	1436·3 (1113·0–1838·0)
Guatemala	262·8 (211·6–321·3)	2866·8 (2412·6–3369·3)	879·5 (682·7–1135·7)	800·4 (613·4–1006·7)	4263·2 (3405·2–5351·1)	45·7 (31·7–65·0)	141·6 (93·6–196·5)	329·0 (268·8–393·5)	1540·8 (1101·8–2090·0)	555·5 (400·4–724·2)	477·3 (194·7–781·3)	1446·2 (1120·7–1849·2)
Honduras	261·7 (211·2–317·8)	2337·7 (1994·8–2736·3)	877·9 (681·2–1134·6)	812·0 (624·8–1014·7)	4091·0 (3273·8–5150·3)	41·3 (28·6–59·3)	126·3 (82·6–176·6)	330·9 (270·6–398·8)	1536·6 (1098·7–2082·6)	554·2 (399·5–722·4)	638·0 (292·2–981·2)	1450·9 (1124·5–1855·3)
Mexico	283·9 (243·9–326·7)	2703·5 (2367·5–3061·8)	906·0 (767·4–1066·6)	914·9 (769·0–1065·4)	3513·3 (2993·8–4100·1)	54·9 (38·4–78·0)	187·9 (125·5–257·4)	371·6 (306·3–444·9)	1115·7 (803·0–1512·2)	602·0 (443·9–783·0)	296·6 (91·1–506·7)	1356·7 (1036·3–1718·4)
Nicaragua	270·7 (218·2–329·2)	2437·3 (2091·6–2854·6)	877·0 (680·9–1133·1)	851·5 (654·8–1072·0)	4365·8 (3488·8–5465·3)	41·0 (28·2–58·4)	125·4 (82·9–174·8)	336·9 (275·2–404·2)	1549·3 (1107·5–2102·9)	555·8 (400·7–724·7)	575·6 (254·8–906·9)	1454·2 (1127·0–1860·4)
Panama	288·1 (229·3–351·3)	2115·2 (1790·6–2476·8)	873·5 (677·5–1129·9)	849·5 (653·5–1075·9)	3656·7 (2920·6–4570·8)	64·5 (44·9–92·1)	219·0 (145·7–303·6)	342·2 (277·4–411·0)	1555·5 (1111·7–2112·1)	555·8 (400·7–724·7)	261·7 (78·8–449·5)	1465·8 (1136·1–1873·5)
Venezuela	275·9 (220·3–335·3)	2398·6 (2049·2–2832·8)	875·8 (679·4–1132·5)	830·5 (636·8–1041·0)	3999·5 (3167·5–5050·4)	50·5 (35·6–72·7)	163·6 (109·6–227·4)	314·3 (258·5–380·3)	2674·9 (1974·6–3620·6)	555·2 (400·2–723·9)	371·4 (133·6–615·8)	1456·8 (1129·1–1863·8)
**Tropical Latin America**	**277·7 (237·7–320·2)**	**2973·4 (2664·7–3302·6)**	**903·7 (765·0–1066·6)**	**1111·1 (933·7–1288·1)**	**7378·6 (6296·1–8605·9)**	**69·9 (49·7–98·6)**	**162·8 (108·6–224·3)**	**353·9 (292·0–425·3)**	**1945·0 (1418·3–2672·7)**	**574·9 (414·7–751·3)**	**357·2 (126·1–596·2)**	**1360·5 (1039·5–1723·8)**
Brazil	277·6 (237·7–320·2)	2976·7 (2671·5–3304·1)	902·5 (763·6–1064·9)	1111·9 (936·9–1289·7)	7410·1 (6327·5–8639·3)	69·9 (49·6–98·6)	163·3 (108·8–225·1)	353·8 (291·9–425·2)	1945·7 (1415·8–2681·0)	576·1 (416·0–753·8)	355·0 (124·1–593·7)	1357·0 (1036·6–1719·2)
Paraguay	277·8 (222·7–339·4)	2829·6 (2412·3–3327·6)	942·7 (730·8–1229·9)	1089·8 (823·2–1356·6)	6304·9 (5077·6–7925·6)	71·3 (50·6–100·2)	147·1 (99·8–203·3)	356·5 (291·7–430·6)	1937·5 (1394·0–2563·5)	546·5 (391·0–718·0)	421·3 (155·3–696·3)	1466·2 (1136·4–1874·1)

Location	Schizophrenia	Major depressive disorder	Dysthymia	Bipolar disorder	Anxiety disorders	Anorexia nervosa	Bulimia nervosa	Autism spectrum disorders	Attention-deficit/hyperactivity disorder	Conduct disorder	Idiopathic developmental intellectual disability	Other mental disorders
**North Africa and Middle East**	**248·2 (203·9–294·9)**	**3322·1 (2843·8–3902·1)**	**1132·0 (893·7–1444·1)**	**758·8 (595·7–939·1)**	**5135·7 (4164·9–6267·2)**	**49·3 (34·6–70·4)**	**168·3 (115·0–229·6)**	**304·4 (251·2–366·1)**	**1245·1 (909·8–1667·4)**	**591·9 (433·4–762·5)**	**1850·5 (1157·7–2571·2)**	**1462·8 (1128·4–1867·2)**
Afghanistan	217·8 (176·2–266·6)	3942·2 (3316·7–4665·8)	1133·2 (874·7–1468·2)	699·6 (545·1–885·6)	4851·0 (3811·3–6058·5)	29·9 (20·7–42·3)	88·3 (58·6–122·6)	287·1 (233·2–347·9)	994·2 (713·9–1348·6)	589·3 (430·3–760·2)	3764·3 (2566·9–4991·3)	1467·9 (1137·8–1875·8)
Algeria	249·1 (197·8–304·0)	3089·8 (2607·3–3672·5)	1134·0 (875·6–1468·7)	756·3 (588·8–953·8)	4772·0 (3792·5–5954·4)	49·5 (34·4–71·6)	162·1 (108·2–228·0)	290·9 (238·3–350·0)	989·4 (710·7–1342·5)	588·2 (429·3–758·8)	1569·3 (974·7–2184·4)	1468·7 (1138·4–1876·5)
Bahrain	270·9 (216·9–332·5)	3550·4 (3018·4–4168·3)	1086·3 (834·3–1407·2)	781·4 (609·4–989·4)	4937·8 (3897·2–6175·5)	64·5 (44·5–93·5)	226·9 (152·6–313·5)	316·6 (255·9–382·9)	1026·4 (736·5–1390·9)	588·4 (429·6–759·0)	761·8 (347·7–1176·8)	1530·9 (1180·2–1955·7)
Egypt	247·6 (198·5–302·4)	2899·6 (2467·3–3443·9)	1127·5 (870·1–1460·8)	723·9 (560·8–910·2)	4588·4 (3675·5–5738·1)	47·0 (32·8–67·9)	152·7 (102·7–213·7)	292·2 (238·0–353·9)	996·8 (716·2–1352·3)	591·8 (432·4–762·4)	1904·3 (1137·7–2678·9)	1478·8 (1145·3–1887·8)
Iran	254·2 (216·6–293·1)	3863·9 (3221·1–4592·0)	1132·1 (965·5–1336·4)	817·2 (686·6–948·6)	7268·1 (6214·5–8502·5)	68·5 (47·9–98·2)	186·4 (126·5–255·1)	370·3 (306·8–441·4)	2665·0 (1948·1–3606·6)	595·8 (427·1–779·4)	1393·6 (833·9–1980·9)	1369·2 (1046·8–1734·5)
Iraq	246·2 (196·3–300·2)	2885·6 (2434·8–3382·2)	1131·7 (873·3–1465·8)	676·5 (526·1–856·4)	5338·0 (4274·9–6681·1)	49·0 (33·9–70·9)	160·3 (109·2–220·2)	286·8 (231·9–345·9)	1040·1 (756·9–1370·6)	613·3 (450·9–791·0)	1503·2 (895·2–2152·8)	1469·8 (1139·1–1878·0)
Jordan	255·1 (204·1–312·3)	3131·1 (2647·1–3748·0)	1118·5 (863·2–1449·0)	781·9 (599·1–976·5)	4933·9 (3909·8–6193·4)	43·2 (30·6–60·7)	150·7 (101·9–207·4)	299·9 (246·0–364·2)	1009·1 (724·5–1366·9)	590·8 (431·8–761·6)	1588·2 (946·2–2264·9)	1488·4 (1151·9–1898·5)
Kuwait	273·8 (216·4–334·0)	3031·6 (2548·7–3608·1)	1117·1 (861·3–1449·5)	810·2 (623·7–1021·6)	4600·0 (3628·0–5713·8)	78·7 (55·1–112·0)	268·2 (180·4–361·2)	305·0 (248·0–370·1)	1000·2 (718·6–1354·4)	589·6 (430·6–760·6)	591·0 (252·7–937·9)	1492·9 (1154·7–1904·0)
Lebanon	253·5 (202·5–308·1)	3625·5 (3037·8–4288·6)	1143·7 (883·9–1482·9)	941·2 (768·6–1113·8)	6051·0 (4811·3–7653·7)	52·3 (36·6–74·8)	173·0 (115·4–237·3)	302·3 (245·6–367·0)	993·7 (714·0–1351·5)	578·4 (429·9–733·8)	1365·6 (805·0–1938·2)	1453·3 (1126·3–1858·6)
Libya	249·9 (202·0–305·3)	3381·7 (2866·9–4058·7)	1129·0 (871·2–1461·9)	770·4 (589·2–971·9)	5324·3 (4277·2–6645·6)	47·4 (32·7–66·9)	154·6 (104·8–214·3)	286·5 (235·2–347·9)	995·2 (714·7–1349·5)	588·8 (429·9–759·7)	1632·8 (995·5–2304·9)	1474·6 (1142·3–1883·5)
Morocco	242·8 (195·3–295·2)	4082·1 (3463·7–4843·6)	1136·0 (877·0–1471·3)	745·1 (571·6–935·2)	4799·7 (3812·3–6003·4)	44·5 (30·9–62·1)	142·6 (95·7–198·0)	287·5 (235·6–347·2)	987·8 (709·6–1340·6)	588·2 (429·3–758·8)	1852·0 (1148·9–2581·2)	1465·0 (1135·5–1872·5)
Oman	264·2 (211·5–322·7)	2973·6 (2517·7–3514·2)	1074·1 (830·4–1395·4)	686·1 (532·3–849·8)	4611·5 (3652·7–5732·7)	57·9 (40·0–84·5)	208·5 (142·4–283·5)	303·0 (248·2–366·0)	1057·6 (759·2–1433·1)	587·5 (428·7–757·9)	849·3 (414·3–1284·0)	1545·7 (1191·2–1979·1)
Palestine	248·2 (198·5–302·3)	5259·5 (4493·5–6188·4)	1133·4 (874·6–1467·8)	790·7 (613·4–991·0)	5545·8 (4428·4–6951·9)	38·4 (26·9–54·7)	120·2 (81·0–165·7)	292·1 (237·8–353·6)	991·7 (712·4–1345·3)	588·4 (429·5–759·0)	2469·9 (1636·7–3338·3)	1466·8 (1136·8–1874·7)
Qatar	285·0 (225·4–351·1)	3002·2 (2538·2–3580·0)	1032·0 (797·5–1340·7)	744·6 (573·9–936·5)	4263·9 (3353·9–5263·8)	71·7 (50·2–102·8)	275·8 (189·4–375·4)	344·7 (283·2–415·7)	1112·0 (796·1–1508·7)	600·3 (440·8–771·0)	428·4 (157·8–709·7)	1606·2 (1234·5–2063·6)
Saudi Arabia	262·9 (208·4–323·1)	3148·3 (2664·7–3700·6)	1163·1 (897·8–1507·8)	762·2 (589·5–947·7)	4554·4 (3598·7–5680·0)	61·1 (42·9–86·3)	218·5 (145·6–295·6)	310·9 (253·2–375·6)	929·5 (669·6–1260·3)	584·8 (428·3–758·3)	586·5 (248·9–924·3)	1517·7 (1171·7–1939·3)
Sudan	232·7 (186·1–284·0)	3393·1 (2853·3–4007·7)	1134·4 (875·1–1469·2)	731·8 (562·7–922·4)	4746·0 (3752·6–5894·8)	36·0 (25·2–50·6)	110·3 (74·0–151·2)	286·7 (233·9–344·7)	987·9 (710·2–1341·2)	590·3 (431·1–761·2)	3007·0 (2037·9–3991·0)	1468·8 (1138·4–1876·3)

Location	Schizophrenia	Major depressive disorder	Dysthymia	Bipolar disorder	Anxiety disorders	Anorexia nervosa	Bulimia nervosa	Autism spectrum disorders	Attention-deficit/hyperactivity disorder	Conduct disorder	Idiopathic developmental intellectual disability	Other mental disorders
Syria	242·4 (194·8–296·0)	3077·6 (2584·4–3664·9)	1147·4 (885·8–1484·6)	773·8 (593·0–972·5)	5607·6 (4451·1–6983·8)	39·1 (27·0–56·1)	119·7 (80·5–163·3)	283·9 (231·3–345·4)	965·9 (694·2–1312·1)	586·5 (427·7–756·5)	2563·5 (1689·6–3461·3)	1451·7 (1125·2–1855·1)
Tunisia	252·5 (201·4–308·9)	4044·9 (3427·8–4791·0)	1138·7 (879·1–1474·7)	787·8 (612·1–984·1)	5164·1 (4120·9–6417·8)	48·4 (34·0–68·3)	157·2 (105·9–216·5)	290·5 (237·2–352·2)	988·0 (710·1–1341·9)	591·0 (431·6–761·8)	1644·4 (1030·1–2294·7)	1460·9 (1132·3–1868·1)
Turkey	248·9 (211·9–289·5)	2888·1 (2444·5–3403·8)	1140·5 (887·9–1495·7)	795·7 (612·2–993·4)	4513·6 (3603·6–5648·7)	52·6 (36·7–74·4)	240·3 (163·4–327·4)	293·3 (239·4–355·8)	1498·0 (1084·5–1989·4)	589·0 (430·0–759·9)	1043·0 (530·6–1561·7)	1466·7 (1136·9–1874·5)
United Arab Emirates	275·3 (218·5–337·2)	2596·7 (2203·7–3099·0)	1049·5 (804·8–1363·4)	752·0 (586·6–956·0)	4243·3 (3386·0–5228·0)	66·0 (45·8–95·6)	235·5 (161·9–324·3)	331·2 (271·9–400·8)	442·1 (309·1–607·4)	587·7 (425·0–764·2)	609·5 (259·5–961·3)	1583·7 (1216·5–2030·6)
Yemen	225·7 (180·7–273·9)	3888·3 (3248·9–4619·9)	1136·1 (877·0–1471·3)	726·0 (565·6–908·2)	4783·7 (3799·9–5963·0)	31·2 (21·8–43·6)	92·8 (62·5–129·4)	286·0 (234·6–347·0)	731·5 (522·1–1009·8)	588·7 (429·7–759·5)	3441·7 (2281·9–4661·1)	1464·7 (1135·3–1872·3)
**South Asia**	**283·5 (242·5–328·7)**	**2683·4 (2363·1–3031·8)**	**1203·5 (1017·5–1426·5)**	**361·4 (303·7–423·5)**	**3045·5 (2594·5–3547·2)**	**36·2 (25·4–51·4)**	**91·3 (59·8–126·9)**	**290·0 (238·4–349·2)**	**609·4 (431·3–832·3)**	**538·2 (383·9–711·9)**	**3555·1 (2434·9–4716·8)**	**1378·6 (1054·5–1748·1)**
Bangladesh	276·2 (220·2–336·3)	3511·5 (2978·5–4144·2)	1237·0 (970·2–1572·7)	389·3 (306·2–490·4)	3180·9 (2528·7–3995·0)	34·3 (23·6–49·1)	81·7 (54·1–113·6)	292·3 (238·4–349·6)	791·4 (566·7–1092·7)	481·0 (340·7–640·4)	532·7 (207·4–859·7)	1462·3 (1133·4–1869·9)
Bhutan	286·7 (227·7–353·0)	2901·9 (2446·4–3431·5)	1223·6 (959·0–1556·1)	392·3 (306·9–491·8)	3267·8 (2608·6–4133·6)	40·5 (28·5–57·4)	103·8 (69·1–142·8)	299·5 (244·7–362·0)	646·8 (453·6–884·7)	483·2 (342·6–642·5)	1378·7 (841·0–1943·1)	1479·4 (1145·7–1888·5)
India	285·2 (244·5–328·4)	2547·8 (2254·1–2880·0)	1198·2 (1018·8–1410·8)	354·1 (299·8–412·5)	2967·4 (2552·8–3439·1)	36·7 (25·9–51·9)	93·3 (61·1–130·2)	288·5 (237·1–347·9)	579·4 (404·6–793·8)	545·3 (388·3–720·2)	4137·7 (2865·2–5450·8)	1368·9 (1046·6–1734·6)
Nepal	267·0 (212·2–327·0)	3795·9 (3265·7–4408·1)	1248·3 (979·4–1585·8)	383·9 (299·7–482·4)	3277·3 (2624·2–4134·7)	31·3 (21·5–45·0)	72·6 (48·1–101·4)	288·6 (235·0–347·6)	630·3 (441·4–863·5)	483·6 (342·7–642·9)	2503·7 (1635·9–3380·8)	1444·5 (1119·5–1845·9)
Pakistan	278·9 (238·7–322·8)	2830·1 (2433·7–3296·5)	1210·5 (1026·3–1429·1)	387·9 (327·6–451·9)	3474·2 (2977·6–4044·6)	35·1 (24·7–50·1)	87·0 (56·7–121·4)	298·1 (244·1–359·3)	657·5 (453·9–903·5)	542·3 (388·9–717·1)	2345·1 (1524·6–3203·5)	1371·1 (1048·4–1736·8)
**Southeast Asia, east Asia, and Oceania**	**305·9 (265·8–349·2)**	**1393·9 (1234·0–1581·2)**	**1378·5 (1160·8–1657·8)**	**226·9 (189·5–267·8)**	**3292·9 (2801·9–3821·7)**	**37·4 (26·1–53·7)**	**74·5 (48·2–104·4)**	**348·1 (287·9–417·2)**	**1622·4 (1212·9–2135·6)**	**511·4 (367·3–666·5)**	**577·5 (288·8–875·4)**	**1383·7 (1059·1–1752·9)**
**East Asia**	**309·2 (272·8–348·0)**	**1406·5 (1250·4–1586·4)**	**1363·6 (1152·3–1608·2)**	**182·0 (153·6–211·1)**	**3180·7 (2712·3–3663·7)**	**38·1 (26·5–55·7)**	**75·2 (48·5–106·0)**	**367·8 (304·4–441·9)**	**2038·0 (1531·9–2662·2)**	**465·0 (326·9–609·6)**	**399·1 (163·6–639·3)**	**1371·0 (1048·4–1737·9)**
China	310·0 (274·5–348·0)	1412·7 (1256·1–1593·0)	1361·5 (1148·7–1600·5)	181·8 (154·0–210·7)	3164·2 (2711·0–3634·3)	38·1 (26·4–55·7)	75·2 (48·5–106·0)	372·3 (307·5–447·6)	2070·8 (1555·0–2705·9)	464·5 (326·7–609·7)	378·0 (146·6–613·6)	1367·7 (1045·6–1733·2)
North Korea	261·3 (210·0–319·7)	1298·7 (1105·8–1537·8)	1426·1 (1110·8–1846·2)	181·9 (143·1–227·9)	3612·1 (2882·3–4437·5)	24·2 (16·8–34·5)	42·6 (27·6–58·6)	261·4 (212·2–315·9)	1319·8 (926·5–1780·7)	462·0 (327·5–621·7)	1529·6 (1003·6–2071·9)	1463·4 (1134·1–1871·5)
Taiwan (province of China)	313·4 (245·9–383·0)	1180·2 (1010·5–1366·2)	1426·0 (1109·9–1850·0)	193·8 (151·3–242·0)	3665·5 (2904·7–4565·8)	57·3 (40·7–81·5)	116·9 (77·0–159·1)	215·8 (183·2–256·4)	940·9 (679·1–1268·7)	501·5 (355·7–659·7)	288·9 (110·0–473·4)	1463·6 (1134·4–1870·6)
**Oceania**	**273·9 (220·9–333·9)**	**1697·5 (1425·6–2017·6)**	**1400·7 (1078·0–1828·9)**	**265·1 (206·8–333·3)**	**4006·8 (3182·9–4990·4)**	**29·0 (20·3–41·4)**	**56·3 (36·4–78·8)**	**289·0 (235·5–349·0)**	**1131·3 (802·6–1567·5)**	**535·1 (374·8–698·5)**	**1213·3 (745·5–1695·1)**	**1471·1 (1139·9–1879·3)**

Location	Schizophrenia	Major depressive disorder	Dysthymia	Bipolar disorder	Anxiety disorders	Anorexia nervosa	Bulimia nervosa	Autism spectrum disorders	Attention-deficit/hyperactivity disorder	Conduct disorder	Idiopathic developmental intellectual disability	Other mental disorders
American Samoa	303·4 (242·9–367·9)	1289·5 (1087·9–1525·9)	1411·2 (1086·2–1846·8)	279·7 (217·2–353·2)	4044·2 (3223·5–5066·2)	42·2 (29·1–60·0)	86·5 (56·9–120·7)	291·8 (238·6–354·9)	1123·3 (796·6–1557·1)	532·7 (372·8–696·2)	479·8 (210·6–758·5)	1462·3 (1133·4–1869·7)
Cook Islands	312·7 (250·1–384·6)	1801·1 (1419·0–2259·9)	1418·2 (1091·2–1854·6)	281·9 (219·7–354·6)	4085·8 (3251·7–5060·9)	51·5 (35·9–73·8)	106·0 (69·8–147·5)	291·6 (237·1–351·3)	1104·1 (781·9–1529·0)	527·4 (368·6–691·3)	325·6 (116·6–538·6)	1454·0 (1126·9–1859·3)
Federated States of Micronesia	269·3 (213·6–331·2)	1549·0 (1302·9–1845·2)	1408·4 (1084·3–1843·0)	264·5 (205·3–331·9)	4034·4 (3238·4–4964·2)	27·8 (19·5–39·8)	53·2 (34·6–75·1)	288·0 (232·8–347·4)	1126·9 (799·3–1561·4)	531·9 (372·1–695·0)	1226·6 (745·4–1736·1)	1465·5 (1135·9–1873·3)
Fiji	292·7 (235·9–358·2)	1560·3 (1322·8–1841·5)	1407·4 (1083·6–1842·2)	274·6 (213·6–346·9)	4039·8 (3233·0–5017·0)	35·3 (24·2–50·8)	71·1 (46·3–98·7)	289·2 (239·3–347·3)	1124·5 (797·5–1558·1)	531·0 (371·3–694·0)	716·3 (361·7–1071·9)	1465·7 (1135·9–1873·7)
Guam	327·2 (259·3–400·8)	1853·9 (1572·4–2183·3)	1400·1 (1077·6–1831·2)	285·0 (221·2–355·7)	4014·0 (3185·4–4967·1)	57·8 (40·5–82·5)	125·8 (83·5–173·8)	296·3 (240·7–360·6)	1133·1 (803·9–1569·4)	532·5 (372·6–695·5)	236·5 (71·9–403·4)	1473·3 (1141·5–1881·6)
Kiribati	255·9 (204·5–312·3)	1592·7 (1341·5–1890·5)	1427·7 (1099·7–1866·5)	257·1 (201·0–323·2)	4098·1 (3279·5–5113·8)	25·0 (17·5–36·2)	45·6 (29·7–63·5)	279·9 (228·7–337·0)	1112·3 (788·5–1542·7)	529·1 (369·9–692·6)	1588·1 (1027·2–2179·6)	1447·7 (1121·9–1851·5)
Marshall Islands	272·6 (219·6–331·9)	1533·0 (1281·5–1821·5)	1401·7 (1079·0–1832·0)	265·9 (207·1–333·8)	4016·4 (3207·8–5024·8)	28·6 (20·0–40·9)	55·0 (36·4–76·3)	289·4 (236·8–351·1)	1123·7 (796·9–1557·0)	529·8 (370·4–692·8)	1191·2 (740·0–1666·5)	1473·0 (1141·3–1881·5)
Nauru	283·0 (227·4–346·0)	1788·5 (1425·0–2254·6)	1419·3 (1093·9–1856·7)	261·2 (205·2–328·8)	4056·5 (3245·5–5044·1)	37·3 (25·8–53·6)	75·5 (49·3–106·1)	284·8 (231·3–345·5)	1124·0 (797·1–1558·0)	532·5 (372·7–695·9)	700·0 (387·7–1035·6)	1458·5 (1130·5–1865·1)
Niue	296·4 (236·1–359·5)	1788·6 (1415·6–2246·7)	1409·1 (1084·9–1843·7)	275·2 (216·0–345·2)	4022·5 (3204·7–5025·2)	38·9 (26·6–55·8)	79·8 (52·4–112·0)	292·3 (237·8–352·2)	1132·2 (803·2–1568·2)	538·1 (377·4–700·7)	591·8 (288·8–913·7)	1463·4 (1134·2–1870·9)
Northern Mariana Islands	312·4 (249·4–382·1)	1429·5 (1213·7–1685·4)	1395·7 (1074·1–1823·3)	280·2 (218·7–352·3)	4004·4 (3180·1–5012·9)	48·4 (34·2–69·7)	103·4 (67·7–142·2)	300·1 (243·3–362·3)	1130·2 (801·7–1565·4)	532·1 (372·2–694·9)	329·4 (115·7–552·4)	1477·1 (1143·9–1886·5)
Palau	297·8 (239·4–362·3)	1764·4 (1403·8–2207·8)	1381·4 (1062·0–1805·5)	269·8 (211·2–342·6)	3946·7 (3132·5–4958·8)	38·2 (27·1–55·2)	82·4 (53·9–114·9)	304·1 (246·8–366·8)	1154·5 (819·6–1602·2)	532·7 (372·6–695·5)	525·1 (233·1–825·4)	1495·0 (1156·5–1908·2)
Papua New Guinea	270·9 (218·5–331·3)	1729·2 (1448·9–2064·1)	1397·9 (1075·6–1824·2)	263·7 (207·6–333·1)	3995·1 (3174·7–4991·7)	28·0 (19·5–40·3)	54·2 (35·0–76·5)	289·0 (235·1–351·6)	1133·1 (803·9–1570·0)	535·9 (375·5–699·3)	1261·5 (775·3–1755·3)	1473·5 (1141·6–1881·8)
Samoa	286·6 (229·6–349·4)	1446·8 (1223·7–1723·8)	1401·0 (1077·6–1831·8)	274·8 (213·7–344·5)	4027·9 (3206·0–5021·8)	32·0 (22·3–45·5)	63·2 (41·1–88·6)	294·0 (239·4–354·8)	1129·7 (801·4–1565·2)	533·0 (373·1–696·2)	855·0 (471·9–1251·0)	1471·2 (1140·0–1879·6)
Solomon Islands	255·2 (203·9–313·1)	1650·8 (1383·2–1975·4)	1405·4 (1081·5–1836·8)	254·8 (200·6–318·1)	4028·6 (3182·2–5021·9)	24·6 (17·4–34·5)	45·4 (29·5–62·8)	288·4 (236·9–348·6)	1125·4 (798·2–1559·9)	533·7 (373·7–697·2)	1630·5 (1054·8–2235·5)	1467·6 (1137·6–1875·4)
Tokelau	289·8 (232·0–351·8)	1776·3 (1409·6–2234·6)	1403·0 (1079·6–1833·8)	274·8 (216·4–347·8)	4003·9 (3204·6–4925·9)	34·2 (23·6–49·1)	69·1 (45·3–97·3)	292·9 (239·9–354·0)	1135·4 (805·4–1573·0)	538·0 (377·1–701·1)	754·0 (387·8–1130·3)	1470·6 (1139·7–1878·5)
Tonga	291·0 (233·7–356·0)	1320·5 (1122·4–1571·9)	1417·6 (1091·4–1853·1)	282·0 (220·7–356·1)	4063·0 (3261·0–4999·5)	32·1 (22·4–46·1)	62·4 (40·6–87·2)	287·2 (233·4–347·7)	1119·6 (793·7–1552·4)	533·1 (373·2–696·7)	858·9 (463·3–1259·5)	1455·2 (1127·8–1861·6)
Tuvalu	275·5 (220·1–333·8)	1779·4 (1414·8–2238·5)	1402·9 (1079·4–1837·1)	267·5 (206·5–334·6)	4007·3 (3153·3–5002·1)	28·1 (19·4–40·1)	55·1 (35·7–77·8)	290·7 (236·5–351·2)	1142·0 (810·5–1581·6)	536·7 (376·1–700·2)	1169·8 (705·7–1638·8)	1472·7 (1141·1–1881·1)

Location	Schizophrenia	Major depressive disorder	Dysthymia	Bipolar disorder	Anxiety disorders	Anorexia nervosa	Bulimia nervosa	Autism spectrum disorders	Attention-deficit/hyperactivity disorder	Conduct disorder	Idiopathic developmental intellectual disability	Other mental disorders
Vanuatu	269·9 (216·6–328·0)	1622·5 (1359·4–1922·8)	1402·4 (1079·1–1830·9)	266·5 (208·1–335·4)	4029·3 (3240·2–5031·5)	27·3 (19·0–38·5)	51·0 (33·5–71·2)	287·8 (233·2–346·4)	1119·4 (793·7–1552·2)	531·5 (371·9–695·2)	1321·8 (823·8–1847·5)	1468·9 (1138·3–1877·3)
**Southeast Asia**	**298·5 (249·6–353·1)**	**1252·6 (1090·7–1441·1)**	**1398·5 (1140·1–1733·7)**	**331·4 (272·5–399·6)**	**3633·2 (3024·1–4315·0)**	**36·3 (25·6–51·2)**	**74·1 (48·4–103·9)**	**312·5 (257·7–374·4)**	**1000·5 (723·4–1365·7)**	**571·7 (417·2–745·2)**	**886·1 (491·8–1289·8)**	**1405·6 (1080·0–1791·7)**
Cambodia	273·5 (218·8–332·5)	1573·7 (1336·2–1879·7)	1427·1 (1099·3–1867·3)	317·9 (246·9–403·2)	4579·8 (3686·2–5708·6)	28·9 (20·5–40·9)	56·0 (36·4–78·6)	307·1 (250·9–375·2)	917·9 (657·6–1249·4)	542·1 (390·2–703·3)	1368·5 (841·1–1934·7)	1450·3 (1123·9–1855·5)
Indonesia	293·7 (250·1–339·1)	1071·9 (912·1–1244·3)	1380·8 (1159·3–1630·4)	330·6 (279·8–386·7)	3548·0 (3024·6–4128·4)	36·6 (25·6–52·0)	75·1 (48·9–105·3)	310·3 (255·9–371·4)	948·1 (683·3–1313·7)	591·7 (432·0–771·7)	895·5 (506·7–1289·4)	1365·3 (1043·5–1729·7)
Laos	280·3 (225·8–342·8)	1378·3 (1161·7–1621·5)	1410·0 (1085·1–1845·3)	324·7 (255·2–406·3)	4570·7 (3750·1–5565·3)	33·4 (22·8–47·2)	66·2 (43·7–92·2)	310·2 (254·6–375·1)	917·0 (657·8–1248·7)	540·2 (388·6–701·7)	1068·2 (636·5–1527·2)	1464·0 (1134·7–1871·7)
Malaysia	316·8 (254·6–390·7)	2335·5 (2012·7–2708·3)	1399·7 (1077·2–1830·9)	339·4 (262·4–430·2)	4599·3 (3675·4–5813·3)	48·2 (33·5–67·9)	102·9 (68·2–141·8)	324·0 (264·7–389·3)	430·3 (303·7–593·0)	542·2 (390·2–703·4)	420·9 (178·8–688·0)	1474·9 (1142·6–1883·7)
Maldives	313·5 (249·3–384·9)	1609·2 (1370·6–1876·8)	1353·7 (1042·0–1768·5)	338·6 (261·0–427·5)	3579·6 (2858·1–4461·0)	37·7 (26·1–55·1)	84·8 (55·2–117·9)	343·9 (279·4–415·9)	962·3 (691·6–1309·2)	545·1 (392·5–705·8)	555·8 (249·2–865·3)	1522·3 (1175·3–1946·9)
Mauritius	310·5 (249·4–381·0)	2476·1 (2083·9–2904·5)	1412·0 (1087·4–1846·7)	337·5 (265·4–423·2)	3815·0 (3048·8–4763·1)	45·5 (31·7–65·4)	94·7 (62·0–129·8)	319·1 (262·0–383·6)	916·1 (657·3–1247·6)	539·6 (388·2–701·2)	468·8 (184·7–754·8)	1461·9 (1133·1–1869·5)
Myanmar	278·2 (224·0–340·0)	792·3 (665·0–937·0)	1428·2 (1099·8–1867·2)	316·7 (245·5–396·4)	3977·0 (3168·2–4931·5)	32·7 (23·1–45·9)	63·8 (41·3–88·6)	304·7 (248·6–365·9)	909·3 (651·5–1238·1)	539·4 (388·0–701·0)	1122·5 (652·1–1596·4)	1447·7 (1121·9–1851·3)
Philippines	292·1 (249·0–337·2)	1443·3 (1232·1–1672·2)	1381·7 (1160·2–1631·3)	336·9 (287·0–391·8)	4563·4 (3925·5–5289·7)	34·2 (23·9–48·9)	71·2 (46·2–100·0)	315·4 (259·9–379·3)	950·6 (685·1–1317·4)	592·2 (432·5–772·0)	913·2 (502·7–1343·8)	1364·8 (1043·1–1729·5)
Seychelles	313·6 (249·5–382·6)	1292·7 (1093·6–1514·7)	1392·0 (1070·6–1820·8)	334·1 (263·2–417·8)	3737·6 (3005·7–4659·4)	48·0 (33·5–69·0)	104·3 (68·2–142·6)	325·0 (265·2–389·8)	931·0 (668·4–1267·9)	540·6 (389·0–702·0)	414·5 (173·8–670·4)	1482·0 (1147·4–1891·9)
Sri Lanka	300·8 (242·7–368·1)	1490·0 (1275·4–1737·6)	1422·0 (1095·1–1858·0)	335·6 (263·1–420·2)	4035·5 (3254·9–4974·6)	39·9 (27·9–57·1)	80·9 (52·7–112·1)	320·6 (262·3–387·2)	911·3 (653·2–1240·8)	539·6 (388·2–701·1)	650·5 (307·0–995·1)	1452·7 (1125·8–1858·3)
Thailand	301·1 (242·5–370·2)	1478·8 (1267·3–1742·5)	1416·8 (1091·0–1851·6)	329·8 (258·8–419·6)	3399·7 (2716·7–4288·4)	43·4 (30·3–62·8)	89·4 (58·3–123·9)	312·1 (254·0–376·7)	1960·3 (1423·0–2612·8)	541·7 (389·8–703·0)	875·7 (469·8–1312·4)	1458·1 (1130·1–1865·0)
Timor-Leste	262·7 (210·4–321·4)	1443·8 (1218·9–1704·7)	1405·2 (1080·8–1837·2)	316·0 (244·7–394·9)	3795·5 (3049·4–4732·3)	32·3 (22·2–46·0)	63·3 (41·8–88·3)	313·5 (257·3–377·9)	919·2 (659·3–1251·2)	542·5 (390·5–703·7)	1052·7 (580·1–1517·0)	1466·8 (1136·9–1874·8)
Vietnam	323·6 (261·4–399·8)	1093·1 (930·4–1289·9)	1417·7 (1091·9–1853·1)	336·1 (262·6–423·3)	2287·3 (1813·6–2826·6)	32·0 (22·1–45·7)	63·5 (41·3–88·5)	314·9 (257·8–380·6)	922·8 (661·5–1255·8)	543·4 (391·2–704·5)	802·5 (452·6–1161·7)	1457·1 (1129·2–1864·0)
**Sub-Saharan Africa**	**214·2 (178·2–254·3)**	**3265·0 (2853·5–3735·5)**	**1399·3 (1139·7–1756·6)**	**566·4 (458·1–690·1)**	**3462·6 (2839·1–4184·2)**	**31·8 (22·4–44·7)**	**75·7 (49·7–104·0)**	**373·5 (307·4–447·6)**	**583·8 (414·2–797·0)**	**592·7 (430·2–763·1)**	**806·1 (398·8–1237·4)**	**1415·7 (1088·2–1808·5)**
**Central sub-Saharan Africa**	**208·5 (166·2–253·9)**	**4340·0 (3671·9–5082·0)**	**1367·3 (1059·6–1803·5)**	**554·3 (432·0–696·3)**	**3864·0 (3089·6–4826·5)**	**28·5 (20·1–39·5)**	**66·1 (43·2–91·0)**	**370·8 (303·3–446·9)**	**569·6 (403·3–776·8)**	**588·6 (432·7–757·8)**	**1052·6 (572·8–1570·3)**	**1456·9 (1129·1–1864·0)**

Location	Schizophrenia	Major depressive disorder	Dysthymia	Bipolar disorder	Anxiety disorders	Anorexia nervosa	Bulimia nervosa	Autism spectrum disorders	Attention-deficit/hyperactivity disorder	Conduct disorder	Idiopathic developmental intellectual disability	Other mental disorders
Angola	219·8 (176·2–267·7)	4552·4 (3799·6–5360·0)	1375·2 (1065·7–1816·0)	553·9 (432·0–699·0)	3934·1 (3157·2–4978·3)	39·7 (27·2–56·5)	97·2 (63·6–133·0)	366·7 (298·9–442·6)	563·1 (398·9–768·5)	586·2 (430·4–754·5)	388·2 (123·1–676·5)	1448·3 (1122·3–1851·8)
Central African Republic	191·4 (153·8–233·1)	4747·3 (4010·2–5594·5)	1371·5 (1062·8–1808·9)	529·9 (414·9–669·2)	4405·6 (3514·8–5534·0)	22·9 (15·9–32·9)	49·8 (32·7–70·3)	367·0 (298·6–442·9)	565·6 (400·7–771·3)	587·6 (431·8–756·4)	1457·1 (823·9–2119·2)	1451·7 (1125·0–1856·6)
Congo	215·3 (172·9–260·8)	4331·6 (3646·6–5099·6)	1365·2 (1059·8–1801·3)	545·3 (423·9–685·2)	3736·7 (2981·4–4677·4)	38·0 (26·4–53·6)	92·1 (59·8–127·8)	371·9 (304·7–448·8)	567·4 (401·8–773·6)	587·4 (431·6–756·1)	451·9 (157·1–753·3)	1461·9 (1133·0–1869·7)
Democratic Republic of the Congo	204·4 (162·4–250·0)	4242·8 (3594·1–5018·1)	1364·6 (1057·5–1799·3)	556·5 (431·5–700·7)	3815·1 (3036·8–4765·3)	23·5 (16·6–33·2)	52·2 (34·1–73·1)	372·2 (303·2–449·6)	571·7 (404·7–779·6)	589·2 (433·3–758·7)	1322·8 (734·5–1942·4)	1459·7 (1131·3–1867·1)
Equatorial Guinea	236·0 (187·7–287·9)	4549·8 (3816·7–5417·9)	1362·7 (1053·6–1799·1)	553·4 (427·5–698·2)	3804·9 (3033·7–4745·9)	57·2 (40·0–80·9)	155·6 (103·7–211·8)	380·6 (314·7–458·7)	599·0 (423·3–816·3)	610·4 (450·8–782·9)	173·2 (30·6–329·7)	1457·6 (1129·7–1863·5)
Gabon	229·7 (184·3–281·3)	4343·6 (3678·7–5100·1)	1371·4 (1064·0–1810·5)	562·2 (435·5–703·9)	3985·1 (3178·8–5016·1)	51·9 (35·7–75·9)	131·7 (86·8–181·9)	369·1 (300·1–446·9)	561·3 (397·3–765·9)	583·8 (428·4–751·1)	238·2 (54·8–436·1)	1453·8 (1126·6–1859·3)
**Eastern sub-Saharan Africa**	**210·8 (174·3–250·2)**	**3524·8 (3090·4–4042·3)**	**1464·0 (1182·7–1840·5)**	**595·6 (480·3–722·6)**	**3716·3 (3050·0–4530·6)**	**28·3 (19·9–40·3)**	**65·2 (42·7–90·6)**	**378·4 (311·7–454·4)**	**572·4 (404·0–779·4)**	**597·0 (436·2–766·8)**	**997·0 (537·0–1504·4)**	**1419·2 (1091·7–1813·0)**
Burundi	200·4 (160·7–244·9)	3367·9 (2880·1–3987·4)	1466·6 (1120·0–1929·4)	579·4 (448·9–728·0)	3638·6 (2906·1–4560·6)	22·7 (15·9–32·3)	49·4 (31·9–69·1)	378·7 (311·7–456·5)	565·1 (400·2–770·4)	574·6 (414·4–740·4)	1506·4 (877·1–2166·8)	1471·0 (1139·6–1880·3)
Comoros	217·2 (174·5–264·1)	2899·7 (2459·5–3445·4)	1471·3 (1125·8–1940·6)	608·7 (470·6–765·0)	3554·6 (2840·9–4476·6)	27·3 (19·1–38·7)	62·9 (40·2–87·1)	381·4 (313·4–460·4)	572·4 (405·3–780·5)	580·5 (419·6–746·3)	795·9 (324·4–1291·3)	1461·3 (1132·6–1868·7)
Djibouti	220·8 (176·2–269·5)	3126·7 (2640·3–3711·9)	1453·2 (1111·0–1909·9)	607·3 (470·7–768·3)	3254·6 (2590·3–4078·6)	32·3 (22·2–46·1)	78·8 (51·8–109·9)	393·1 (323·6–472·5)	590·8 (418·4–804·0)	595·6 (433·1–761·7)	549·2 (179·0–925·3)	1480·0 (1146·0–1888·7)
Eritrea	207·4 (166·6–251·7)	3505·4 (3001·3–4135·4)	1473·0 (1127·9–1943·6)	584·0 (452·6–737·9)	3732·3 (2989·8–4710·7)	26·8 (18·7–38·5)	61·9 (40·0–86·3)	376·8 (307·9–452·7)	574·1 (406·5–782·8)	581·4 (420·5–747·4)	905·0 (423·4–1409·3)	1455·7 (1128·1–1862·7)
Ethiopia	213·4 (182·1–247·2)	3343·4 (2910·5–3852·5)	1432·1 (1226·6–1693·9)	614·4 (520·4–719·6)	3465·6 (2966·9–4083·7)	26·4 (18·5–37·6)	60·7 (39·2–84·5)	383·2 (315·6–459·4)	581·1 (404·9–802·6)	626·5 (465·2–802·8)	1001·5 (510·9–1520·0)	1367·3 (1045·1–1731·5)
Kenya	216·6 (184·7–250·8)	3411·8 (3023·3–3863·1)	1443·7 (1208·2–1706·7)	601·7 (510·1–704·0)	3418·0 (2921·7–4005·4)	31·9 (22·4–45·6)	76·1 (49·5–106·2)	381·1 (313·8–457·9)	579·3 (403·7–800·2)	625·4 (464·2–801·6)	694·3 (318·5–1094·9)	1360·9 (1039·6–1723·7)
Madagascar	212·3 (170·7–257·2)	3394·6 (2896·7–3965·6)	1473·2 (1127·5–1940·5)	597·4 (464·1–747·5)	3963·2 (3150·3–5003·6)	26·7 (18·4–38·7)	60·7 (39·7–84·3)	378·4 (308·4–455·9)	570·8 (404·3–778·5)	580·2 (419·4–746·1)	939·8 (459·7–1456·8)	1461·6 (1132·8–1868·9)
Malawi	201·8 (161·5–246·2)	2717·3 (2302·1–3201·9)	1482·6 (1135·0–1952·3)	577·4 (447·0–723·5)	3845·7 (3067·5–4836·2)	25·0 (17·5–35·7)	55·4 (36·5–77·1)	372·1 (303·6–447·9)	564·3 (399·6–769·7)	576·8 (416·5–742·6)	1144·9 (582·8–1695·9)	1451·4 (1124·7–1856·2)
Mozambique	203·9 (163·0–248·5)	3646·0 (3116·4–4290·5)	1490·2 (1141·6–1961·2)	578·6 (445·7–730·8)	4035·2 (3240·2–5142·8)	26·2 (18·1–37·3)	58·4 (38·1–82·9)	369·6 (301·5–446·2)	560·7 (397·1–765·0)	575·4 (415·1–741·1)	1156·0 (645·4–1685·2)	1445·3 (1120·0–1847·4)
Rwanda	210·0 (169·6–255·2)	3766·4 (3225·8–4416·1)	1485·3 (1138·7–1960·6)	588·8 (458·2–735·5)	3653·4 (2933·6–4585·9)	28·7 (19·9–41·9)	66·2 (42·8–92·9)	372·4 (306·6–448·2)	566·6 (401·2–773·4)	577·8 (417·3–743·7)	897·3 (472·0–1362·2)	1446·7 (1121·1–1850·2)

Location	Schizophrenia	Major depressive disorder	Dysthymia	Bipolar disorder	Anxiety disorders	Anorexia nervosa	Bulimia nervosa	Autism spectrum disorders	Attention-deficit/hyperactivity disorder	Conduct disorder	Idiopathic developmental intellectual disability	Other mental disorders
Somalia	188·4 (150·3–230·7)	3515·9 (3016·4–4162·0)	1471·7 (1127·4–1942·6)	581·2 (452·6–728·4)	3272·9 (2600·3–4098·4)	15·6 (11·0–22·1)	30·9 (20·1–43·5)	379·0 (308·8–459·5)	577·9 (409·4–787·9)	584·4 (423·2–750·8)	3152·2 (2015·7–4286·5)	1456·6 (1128·8–1863·4)
South Sudan	229·3 (184·4–276·8)	3402·1 (2887·1–3986·4)	1480·5 (1133·2–1945·8)	611·0 (469·3–765·6)	4977·7 (3967·6–6255·4)	44·2 (30·8–64·2)	108·6 (70·6–149·2)	378·8 (311·3–454·6)	571·4 (405·1–778·7)	586·1 (424·6–752·6)	236·7 (42·1–474·1)	1459·5 (1131·2–1866·3)
Tanzania	213·3 (170·5–260·0)	3295·7 (2782·6–3873·1)	1484·0 (1136·1–1950·5)	591·1 (455·1–739·8)	3812·6 (3029·7–4838·8)	31·9 (21·8–45·7)	74·1 (48·7–103·1)	373·5 (305·5–450·8)	563·2 (399·0–768·3)	577·1 (416·6–742·9)	711·6 (322·1–1114·2)	1453·4 (1126·4–1858·6)
Uganda	207·9 (167·1–253·0)	5337·6 (4567·5–6257·5)	1497·8 (1156·1–1942·1)	581·8 (445·7–727·8)	4080·2 (3273·2–5151·0)	29·2 (20·1–42·0)	67·2 (43·6–94·6)	378·8 (309·3–454·5)	568·1 (402·6–774·8)	581·2 (420·2–747·1)	834·7 (394·3–1275·1)	1447·4 (1121·7–1850·8)
Zambia	212·9 (170·0–259·0)	2850·0 (2414·4–3367·8)	1473·1 (1126·2–1938·5)	574·5 (448·5–724·0)	3969·2 (3180·8–5002·1)	34·3 (24·0–49·5)	81·4 (53·6–114·3)	375·8 (308·5–452·4)	566·7 (401·2–772·6)	576·9 (416·5–742·7)	554·8 (203·4–917·8)	1462·2 (1133·3–1870·2)
**Southern sub-Saharan Africa**	**220·9 (187·5–256·8)**	**2919·4 (2569·5–3281·7)**	**1351·4 (1127·8–1623·5)**	**553·2 (459·0–654·1)**	**3658·0 (3100·4–4307·8)**	**42·7 (30·1–61·3)**	**109·2 (71·7–149·9)**	**371·6 (304·9–447·7)**	**575·3 (404·0–789·5)**	**617·9 (456·6–801·4)**	**443·4 (176·1–722·3)**	**1379·9 (1057·1–1747·4)**
Botswana	225·2 (178·9–275·4)	2994·2 (2538·2–3529·5)	1369·9 (1060·7–1806·7)	540·9 (422·3–678·1)	3553·4 (2817·3–4440·4)	50·3 (35·1–72·0)	128·6 (85·0–179·9)	372·0 (303·6–451·4)	570·2 (403·9–777·7)	589·8 (433·9–759·2)	275·4 (76·5–480·7)	1452·5 (1125·6–1858·3)
Eswatini	217·6 (175·7–265·9)	3162·3 (2678·7–3714·6)	1376·2 (1064·2–1814·9)	534·7 (416·3–671·3)	3492·0 (2767·9–4402·8)	43·5 (29·9–62·9)	107·6 (70·6–147·2)	367·0 (302·5–442·8)	570·9 (404·6–778·3)	592·5 (436·8–761·8)	385·2 (131·5–642·6)	1443·1 (1118·2–1846·0)
Lesotho	204·7 (164·3–248·1)	4318·1 (3688·7–5069·3)	1368·1 (1058·2–1802·4)	532·2 (413·4–666·8)	3555·5 (2796·2–4493·9)	32·2 (22·3–46·2)	76·5 (50·0–105·3)	369·5 (300·8–445·7)	571·5 (404·6–779·6)	589·1 (433·2–758·5)	687·7 (299·1–1091·4)	1452·8 (1125·8–1859·2)
Namibia	222·6 (179·4–273·1)	2318·8 (1992·5–2707·4)	1373·6 (1063·3–1812·4)	553·2 (425·8–694·2)	3491·9 (2767·6–4363·4)	46·0 (32·4–65·6)	115·2 (75·0–157·5)	367·9 (300·7–445·1)	566·3 (401·0–772·8)	587·3 (431·5–756·1)	321·9 (98·6–557·4)	1448·3 (1122·4–1852·4)
South Africa	224·8 (193·0–259·0)	3066·9 (2715·1–3449·0)	1342·2 (1137·7–1574·2)	557·0 (473·6–651·4)	3786·1 (3254·7–4408·8)	46·1 (32·3–65·9)	119·2 (78·5–163·8)	373·6 (307·8–448·3)	580·5 (404·5–801·7)	634·4 (467·1–821·3)	318·8 (100·3–546·6)	1354·2 (1034·4–1715·7)
Zimbabwe	202·5 (162·6–247·0)	2086·0 (1789·1–2446·0)	1378·5 (1066·7–1819·3)	538·6 (420·8–679·9)	3137·0 (2486·3–3931·0)	30·0 (21·0–43·5)	69·0 (44·6–95·9)	364·4 (295·9–438·7)	563·5 (399·2–768·9)	587·4 (431·6–756·1)	875·1 (447·2–1337·7)	1442·5 (1117·8–1844·8)
**Western sub-Saharan Africa**	**217·1 (181·1–256·5)**	**2817·9 (2462·3–3217·0)**	**1359·4 (1113·6–1685·7)**	**546·6 (445·2–661·4)**	**3066·5 (2532·6–3683·3)**	**34·0 (23·9–48·1)**	**81·2 (53·6–111·7)**	**370·6 (305·5–443·3)**	**599·6 (421·8–832·2)**	**586·7 (423·0–763·5)**	**626·0 (282·1–1001·2)**	**1408·6 (1081·2–1797·9)**
Benin	213·5 (171·1–259·7)	3195·2 (2710·8–3738·5)	1371·1 (1063·6–1810·8)	545·6 (426·5–684·6)	3228·8 (2562·1–4052·8)	30·1 (21·0–42·7)	70·0 (45·6–97·7)	370·3 (304·2–446·2)	587·8 (406·3–825·5)	568·1 (407·1–745·9)	776·6 (355·8–1201·6)	1454·3 (1127·1–1860·1)
Burkina Faso	207·9 (166·6–253·5)	3113·8 (2664·1–3623·3)	1377·8 (1067·8–1819·4)	535·3 (413·5–671·4)	3116·7 (2474·0–3964·3)	28·5 (20·1–40·4)	65·0 (43·1–90·4)	366·7 (301·3–441·1)	586·5 (405·4–823·7)	569·4 (408·3–747·3)	874·8 (410·4–1340·2)	1445·6 (1120·3–1847·9)
Cabo Verde	229·6 (182·8–280·1)	3620·5 (3102·5–4244·8)	1360·4 (1054·0–1792·3)	578·0 (451·4–725·3)	3085·5 (2426·6–3839·2)	39·4 (27·6–56·4)	98·3 (64·9–137·7)	380·4 (311·0–460·6)	596·8 (412·6–839·0)	569·7 (408·5–747·6)	354·1 (101·5–619·3)	1464·3 (1134·9–1872·2)
Cameroon	213·2 (170·8–260·8)	3364·6 (2897·2–3890·9)	1366·2 (1060·5–1803·7)	534·4 (415·7–673·9)	3200·0 (2541·0–3964·7)	33·1 (22·9–46·9)	79·3 (52·8–108·8)	373·0 (307·0–450·6)	591·5 (408·8–830·7)	569·8 (408·6–747·6)	589·6 (228·3–979·8)	1460·7 (1132·1–1868·2)

Location	Schizophrenia	Major depressive disorder	Dysthymia	Bipolar disorder	Anxiety disorders	Anorexia nervosa	Bulimia nervosa	Autism spectrum disorders	Attention-deficit/hyperactivity disorder	Conduct disorder	Idiopathic developmental intellectual disability	Other mental disorders
Chad	210·6 (169·1–257·0)	3922·7 (3344·9–4583·8)	1365·7 (1060·6–1802·8)	537·8 (420·6–673·5)	3369·7 (2658·8–4216·3)	29·9 (20·9–42·7)	69·2 (45·8–95·8)	373·5 (304·8–450·9)	587·2 (405·7–824·0)	567·9 (406·9–745·8)	840·0 (406·4–1297·4)	1465·5 (1135·9–1873·3)
Côte d'Ivoire	213·5 (172·0–259·1)	2674·0 (2282·2–3130·7)	1354·1 (1053·3–1786·3)	531·5 (413·9–675·0)	3240·3 (2547·0–4012·0)	33·8 (23·3–48·9)	81·4 (54·2–113·1)	379·7 (309·9–456·1)	600·5 (414·6–843·0)	574·2 (412·9–753·1)	529·6 (183·5–883·9)	1476·7 (1143·5–1886·3)
The Gambia	212·3 (169·9–259·9)	4334·6 (3705·4–5111·2)	1367·8 (1062·1–1806·1)	548·5 (426·1–687·8)	3312·7 (2631·9–4182·9)	28·5 (20·1–40·5)	65·5 (43·2–90·5)	371·9 (302·2–447·6)	587·3 (405·9–824·4)	567·1 (406·3–744·7)	807·2 (349·3–1271·9)	1459·4 (1131·1–1866·9)
Ghana	216·9 (172·5–263·7)	3099·0 (2639·0–3636·7)	1375·9 (1065·8–1817·2)	543·9 (418·0–680·3)	3000·5 (2356·6–3735·2)	34·9 (24·4–50·8)	83·7 (55·1–116·5)	368·7 (303·1–444·0)	589·0 (407·1–827·4)	570·5 (409·2–748·5)	543·3 (214·3–904·1)	1446·7 (1121·1–1849·7)
Guinea	209·1 (168·5–253·2)	3113·1 (2644·7–3657·9)	1376·0 (1067·9–1818·5)	537·3 (418·7–669·6)	3338·9 (2620·3–4182·8)	28·9 (20·3–41·6)	65·7 (43·0–90·2)	368·1 (300·0–442·7)	582·9 (402·8–817·8)	567·8 (406·9–745·7)	812·2 (354·5–1280·1)	1451·6 (1125·0–1855·5)
Guinea-Bissau	203·8 (164·5–249·2)	3241·9 (2766·6–3805·2)	1375·5 (1066·1–1817·0)	526·6 (409·0–660·1)	3093·5 (2446·8–3840·6)	27·3 (18·9–39·0)	61·9 (40·5–86·2)	366·2 (296·3–442·2)	586·8 (405·5–824·2)	568·6 (407·5–746·4)	825·7 (350·5–1321·9)	1447·9 (1122·0–1851·2)
Liberia	207·0 (166·6–251·4)	3330·3 (2836·8–3911·5)	1360·8 (1058·0–1796·1)	543·3 (420·0–681·0)	3506·7 (2790·6–4378·2)	24·0 (16·7–34·7)	53·2 (34·6–75·5)	375·6 (306·1–453·7)	592·4 (409·2–831·4)	569·6 (408·5–747·5)	1203·7 (640·1–1782·2)	1469·4 (1138·6–1878·0)
Mali	211·2 (168·2–257·8)	2251·3 (1914·4–2659·2)	1367·0 (1062·3–1806·6)	542·0 (417·3–679·9)	2726·0 (2163·6–3396·9)	28·5 (19·5–41·4)	65·3 (42·4–91·5)	373·3 (305·6–449·0)	589·1 (407·0–826·8)	569·6 (408·4–747·4)	827·2 (364·7–1295·0)	1462·4 (1133·5–1870·0)
Mauritania	220·3 (176·2–268·8)	2366·0 (2014·5–2792·3)	1369·8 (1064·0–1810·6)	556·1 (435·8–694·3)	2746·2 (2166·9–3428·3)	33·9 (23·8–48·3)	80·7 (53·8–112·7)	374·4 (306·5–452·8)	587·1 (405·9–824·4)	567·6 (406·7–745·3)	557·6 (208·7–913·3)	1458·4 (1130·4–1865·4)
Niger	207·7 (166·7–253·4)	2974·7 (2525·0–3490·3)	1369·0 (1062·6–1808·8)	542·8 (417·0–682·0)	2967·8 (2332·1–3679·7)	24·7 (17·2–35·1)	54·9 (35·8–76·7)	371·2 (305·3–447·4)	589·1 (407·4–827·7)	568·0 (407·1–745·9)	1075·3 (527·3–1637·5)	1458·0 (1130·0–1864·9)
Nigeria	222·6 (190·3–257·0)	2559·1 (2215·9–2918·0)	1348·8 (1143·6–1584·2)	554·2 (469·1–647·3)	2991·8 (2568·5–3493·3)	37·9 (26·6–54·0)	92·2 (60·8–128·2)	369·1 (304·5–441·9)	609·7 (432·8–839·5)	604·3 (433·9–782·7)	486·5 (190·5–788·3)	1352·4 (1032·4–1711·9)
São Tomé and Príncipe	222·8 (179·0–271·3)	2430·9 (2074·0–2889·5)	1362·0 (1057·2–1797·0)	564·8 (439·1–707·8)	3083·8 (2436·8–3905·4)	33·1 (22·7–46·7)	78·7 (52·2–109·4)	376·1 (304·0–452·0)	592·5 (409·6–832·5)	568·3 (407·2–746·2)	528·5 (182·0–874·9)	1465·6 (1136·0–1873·6)
Senegal	216·2 (173·7–262·2)	2758·4 (2345·9–3227·1)	1368·2 (1061·7–1808·0)	552·7 (429·5–693·0)	2893·4 (2262·3–3589·2)	30·9 (21·3–44·7)	73·3 (48·2–102·5)	375·6 (304·9–452·4)	596·4 (412·0–838·2)	574·4 (413·2–753·3)	586·1 (202·2–993·7)	1457·8 (1129·9–1864·4)
Sierra Leone	208·0 (166·5–254·6)	3145·1 (2671·3–3705·2)	1364·1 (1060·0–1800·9)	538·3 (419·0–676·7)	3663·3 (2904·5–4604·2)	26·7 (18·7–38·2)	60·8 (39·4–85·1)	372·4 (304·3–447·7)	588·1 (406·5–825·6)	567·1 (406·1–744·6)	1021·0 (534·4–1537·9)	1464·8 (1135·4–1873·0)
Togo	208·7 (166·7–255·8)	3245·9 (2757·7–3822·5)	1376·4 (1065·5–1817·5)	539·5 (418·8–681·1)	3592·5 (2848·6–4512·5)	27·4 (19·3–38·9)	63·2 (40·8–86·9)	368·3 (300·3–444·6)	592·5 (409·2–832·5)	573·5 (412·3–752·5)	922·6 (456·0–1425·5)	1444·6 (1119·4–1846·9)

Note: North Africa & Middle East and South Asia are both super-regions and regions.

eFigure 3: Proportion of global mental disorder DALYs attributable to each disorder for both sexes and all ages in 2019

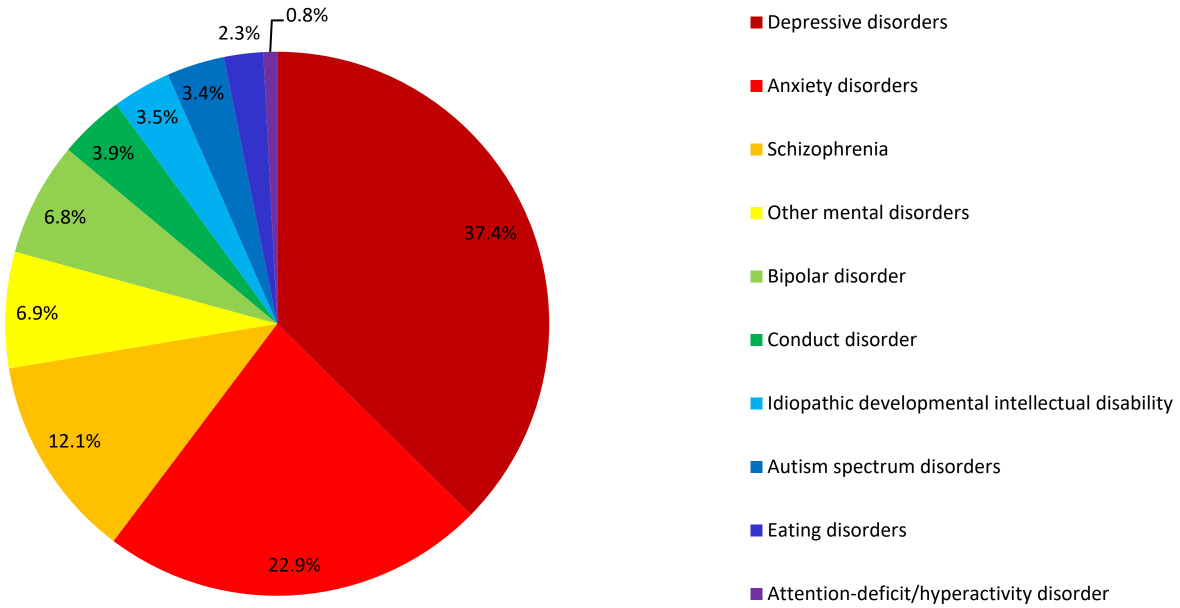

eTable 9a: All-cause rankings of YLD and DALY rates for mental disorders for males by all ages and five age groups in 2019

YLDs/DALYs for males					
All ages	***0-14 years***	***15-24 years***	***25-49 years***	***50-69 years***	***70+ years***
4/21 Depressive disorders	3/20 Conduct disorder	2/5 Depressive disorders	3/11 Depressive disorders	5/18 Depressive disorders	11/31 Depressive disorders
8/29 Anxiety disorders	10/33 Anxiety disorders	4/9 Anxiety disorders	5/18 Schizophrenia	15/37 Anxiety disorders	21/55 Anxiety disorders
17/43 Schizophrenia	15/40 Autism spectrum disorders	9/29 Conduct disorder	8/20 Anxiety disorders	17/42 Schizophrenia	26/62 Other mental disorders
23/56 Other mental disorders	18/51 ID intellectual disability	12/32 Bipolar disorder	16/31 Other mental disorders	19/50 Other mental disorders	38/84 Schizophrenia
26/64 Bipolar disorder	29/66 Depressive disorders	15/39 Schizophrenia	17/41 Bipolar disorder	29/64 Bipolar disorder	43/93 Bipolar disorder
31/77 Autism spectrum disorders	35/75 ADHD	22/44 Autism spectrum disorders	31/56 Autism spectrum disorders	44/88 Autism spectrum disorders	52/106 Autism spectrum disorders
32/78 Conduct disorder	53/97 Bipolar disorder	27/51 ID intellectual disability	40/73 ID intellectual disability	65/118 ID intellectual disability	91/129 ID intellectual disability
45/92 ID intellectual disability	67/105 Eating disorders	28/54 Other mental disorders	42/77 Eating disorders	120/148 ADHD	146/153 ADHD
63/119 Eating disorders	87/119 Schizophrenia	35/63 Eating disorders	71/120 ADHD	N/A Eating disorders	N/A Eating disorders
71/130 ADHD	89/122 Other mental disorders	46/75 ADHD	N/A Conduct disorder	N/A Conduct disorder	N/A Conduct disorder

Note: This table shows YLD and DALY rankings for each mental disorder. Mental disorders are ranked out of all Level 3 causes within the GBD study. Disorders are ordered from highest to lowest ranking for the overall age group (ie, all ages). Each colour represents a different mental disorder. Cells marked 'N/A' (in grey) show disorders for which burden was not estimated within this age group. ID=idiopathic developmental. ADHD=attention-deficit/hyperactivity disorder. DALYs=disability-adjusted life-years. YLDs=years lived with disability.

eTable 9b: All-cause rankings of YLD and DALY rates for mental disorders for females by all ages and five age groups in 2019

**YLDs/DALYs for females**					
All ages	***0-14 years***	***15-24 years***	***25-49 years***	***50-69 years***	***70+ years***
4/11 Depressive disorders	4/21 Anxiety disorders	2/2 Depressive disorders	4/5 Depressive disorders	4/8 Depressive disorders	11/23 Depressive disorders
8/20 Anxiety disorders	10/29 Conduct disorder	4/5 Anxiety disorders	6/10 Anxiety disorders	15/28 Anxiety disorders	18/33 Anxiety disorders
19/44 Schizophrenia	19/45 Depressive disorders	13/25 Bipolar disorder	11/21 Schizophrenia	19/38 Schizophrenia	29/68 Other mental disorders
29/56 Bipolar disorder	20/50 ID intellectual disability	18/35 Eating disorders	18/35 Bipolar disorder	24/51 Other mental disorders	36/76 Schizophrenia
31/63 Other mental disorders	35/73 Autism spectrum disorders	23/39 Schizophrenia	23/41 Other mental disorders	26/57 Bipolar disorder	41/85 Bipolar disorder
44/88 ID intellectual disability	51/96 Bipolar disorder	26/47 Conduct disorder	33/54 Eating disorders	58/116 ID intellectual disability	80/129 ID intellectual disability
47/92 Eating disorders	56/99 ADHD	28/48 ID intellectual disability	42/65 ID intellectual disability	66/123 Autism spectrum disorders	81/130 Autism spectrum disorders
54/99 Conduct disorder	60/101 Eating disorders	45/64 Other mental disorders	61/99 Autism spectrum disorders	134/155 ADHD	150/157 ADHD
68/120 Autism spectrum disorders	91/122 Schizophrenia	54/79 Autism spectrum disorders	91/137 ADHD	N/A Eating disorders	N/A Eating disorders
98/150 ADHD	94/129 Other mental disorders	68/99 ADHD	N/A Conduct disorder	N/A Conduct disorder	N/A Conduct disorder

Note: This table shows YLD and DALY rankings for each mental disorder. Mental disorders are ranked out of all Level 3 causes within the GBD study. Disorders are ordered from highest to lowest ranking for the overall age group (ie, all ages). Each colour represents a different mental disorder. Cells marked 'N/A' (in grey) show disorders for which burden was not estimated within this age group. ID=idiopathic developmental. ADHD=attention-deficit/hyperactivity disorder. DALYs=disability-adjusted life-years. YLDs=years lived with disability.

eTable 10: Age-standardised rates and number of DALYs for mental disorders by location in 2019

Location	Age-standardised DALY rates per 100,000	95% uncertainty intervals (age-standardised rates)	Total DALYs in 1,000s	95% uncertainty intervals (total DALYs)
**Global**	**1566·2**	**(1160·1–2042·8)**	**125311·3**	**(92995·0–163177·9)**
**Central Europe, eastern Europe, and central Asia**	**1400·9**	**(1039·5–1840·6)**	**6450·2**	**(4793·7–8461·7)**
**Central Asia**	**1346·7**	**(993·7–1776·3)**	**1254·6**	**(925·7–1655·3)**
Armenia	1404·7	(1033·8–1855·4)	46·5	(34·6–61·2)
Azerbaijan	1286·8	(946·7–1694·7)	141·1	(103·8–186·2)
Georgia	1400·1	(1033·6–1848·2)	57·2	(42·3–75·3)
Kazakhstan	1383·7	(1024·4–1823·0)	258·1	(192·2–340·8)
Kyrgyzstan	1383·4	(1014·5–1828·4)	86·5	(63·5–114·0)
Mongolia	1494·0	(1100·9–1970·6)	49·9	(36·7–65·5)
Tajikistan	1298·6	(958·3–1707·9)	115·2	(85·2–152·5)
Turkmenistan	1333·6	(982·7–1756·1)	66·6	(49·0–88·0)
Uzbekistan	1319·6	(969·9–1743·9)	433·3	(318·5–572·3)
**Central Europe**	**1341·6**	**(992·9–1773·0)**	**1741·8**	**(1289·0–2274·4)**
Albania	1365·4	(1008·0–1813·3)	40·9	(30·2–53·9)
Bosnia and Herzegovina	1391·4	(1020·1–1834·0)	52·7	(39·0–69·4)
Bulgaria	1348·6	(990·8–1784·8)	108·8	(79·6–142·9)
Croatia	1451·3	(1059·1–1919·1)	71·6	(52·5–93·8)
Czechia	1384·9	(1014·4–1824·7)	167·5	(123·1–220·0)
Hungary	1393·9	(1013·9–1851·4)	156·5	(114·8–206·7)
Montenegro	1417·3	(1039·0–1868·5)	9·8	(7·2–12·9)
North Macedonia	1360·1	(1002·2–1787·3)	33·1	(24·4–43·4)
Poland	1259·0	(929·6–1651·3)	544·9	(401·8–708·4)
Romania	1368·1	(1007·4–1804·9)	298·8	(221·5–391·8)
Serbia	1383·8	(1018·8–1825·3)	136·7	(100·0–179·0)
Slovakia	1373·1	(1014·9–1817·2)	85·3	(62·8–111·6)
Slovenia	1462·3	(1071·4–1928·5)	35·1	(25·9–45·9)
**Eastern Europe**	**1462·2**	**(1083·3–1926·2)**	**3453·8**	**(2562·0–4531·6)**
Belarus	1601·8	(1173·4–2105·4)	173·7	(127·8–229·0)
Estonia	1572·8	(1144·8–2081·1)	24·0	(17·5–31·5)
Latvia	1614·1	(1184·0–2122·2)	35·8	(26·3–47·1)
Lithuania	1716·2	(1256·4–2262·0)	55·6	(40·6–73·2)
Moldova	1534·9	(1125·3–2024·9)	65·1	(47·4–85·1)
Russia	1407·6	(1039·4–1850·1)	2290·6	(1692·9–3009·9)
Ukraine	1575·6	(1168·8–2080·6)	809·0	(601·6–1059·4)
**High income**	**1924·4**	**(1423·8–2520·8)**	**22095·2**	**(16419·8–28834·3)**
**Australasia**	**2393·1**	**(1756·3–3140·9)**	**713·6**	**(526·9–932·7)**
Australia	2399·5	(1750·7–3159·3)	607·7	(447·0–797·0)
New Zealand	2354·3	(1724·7–3112·9)	106·0	(78·0–139·5)
**High income Asia Pacific**	**1333·1**	**(977·2–1748·1)**	**2690·6**	**(1986·6–3489·4)**
Brunei	1248·8	(911·1–1653·7)	5·9	(4·3–7·9)
Japan	1333·3	(981·4–1749·9)	1803·3	(1336·1–2339·9)
Singapore	1377·7	(1005·3–1808·3)	86·8	(63·7–113·9)
South Korea	1334·6	(975·7–1758·5)	794·6	(582·7–1039·0)
**High income North America**	**2100·2**	**(1570·7–2748·8)**	**7981·4**	**(5984·0–10366·6)**
Canada	1756·8	(1287·5–2314·8)	665·0	(487·1–864·9)

Location	Age-standardised DALY rates per 100,000	95% uncertainty intervals (age-standardised rates)	Total DALYs in 1,000s	95% uncertainty intervals (total DALYs)
Greenland	2357·5	(1725·1–3114·9)	1·4	(1·0–1·8)
USA	2137·7	(1596·6–2794·9)	7314·8	(5485·0–9495·3)
**Southern Latin America**	**1791·7**	**(1301·5–2356·6)**	**1253·4**	**(914·2–1644·1)**
Argentina	1706·4	(1248·3–2250·2)	799·6	(586·0–1051·5)
Chile	1994·2	(1452·8–2631·3)	389·0	(285·2–511·2)
Uruguay	1793·7	(1312·7–2371·5)	64·7	(47·4–84·9)
**Western Europe**	**2002·5**	**(1465·7–2655·8)**	**9456·2**	**(6960·6–12392·8)**
Andorra	1956·5	(1429·2–2590·8)	1·8	(1·3–2·4)
Austria	1905·2	(1388·1–2517·6)	182·9	(134·3–240·0)
Belgium	1875·1	(1368·7–2467·3)	228·2	(167·5–299·8)
Cyprus	1915·2	(1390·1–2540·7)	27·3	(19·9–36·0)
Denmark	1794·5	(1302·6–2376·0)	112·7	(82·6–148·1)
England	1965·6	(1440·0–2602·5)	1189·7	(876·3–1557·3)
Finland	1887·4	(1387·4–2496·9)	110·4	(81·2–145·3)
France	2045·3	(1488·7–2717·3)	1443·0	(1054·7–1901·6)
Germany	1898·5	(1368·3–2514·7)	1761·9	(1296·3–2308·1)
Greece	2259·8	(1663·9–2967·5)	259·5	(192·6–340·3)
Iceland	1761·2	(1283·1–2342·1)	6·4	(4·7–8·5)
Ireland	2201·8	(1612·1–2884·0)	113·5	(83·3–147·8)
Israel	1906·2	(1394·8–2492·7)	177·2	(130·3–231·6)
Italy	1953·9	(1434·1–2586·2)	1297·5	(961·1–1685·8)
Luxembourg	1849·7	(1352·3–2442·0)	12·4	(9·1–16·3)
Malta	1902·9	(1387·4–2522·0)	9·0	(6·6–11·8)
Monaco	2110·2	(1522·3–2815·4)	0·9	(0·6–1·1)
Netherlands	2069·4	(1519·2–2734·4)	384·9	(282·7–505·2)
Northern Ireland	2207·1	(1593·5–2925·4)	45·3	(32·8–59·7)
Norway	1945·3	(1427·4–2579·7)	110·5	(81·1–145·4)
Portugal	2316·5	(1672·3–3081·5)	277·4	(204·4–363·2)
San Marino	2048·2	(1501·8–2723·7)	0·7	(0·5–1·0)
Scotland	1837·1	(1342·7–2423·5)	109·0	(80·1–142·9)
Spain	2192·2	(1614·3–2905·1)	1103·0	(816·9–1457·1)
Sweden	2016·6	(1473·7–2652·7)	218·8	(161·1–286·6)
Switzerland	2088·6	(1523·3–2768·6)	199·1	(145·9–262·8)
Wales	1905·8	(1390·9–2507·5)	64·9	(47·8–85·4)
**Latin America and Caribbean**	**1831·3**	**(1343·6–2404·4)**	**11154·3**	**(8189·6–14636·5)**
**Andean Latin America**	**1700·6**	**(1237·5–2265·5)**	**1085·3**	**(787·9–1449·5)**
Bolivia	1842·1	(1349·0–2448·8)	210·8	(154·5–280·4)
Ecuador	1799·3	(1312·7–2383·5)	317·3	(230·5–422·5)
Peru	1606·5	(1165·0–2139·2)	557·2	(403·3–741·5)
**Caribbean**	**1777·0**	**(1294·0–2339·1)**	**869·8**	**(634·1–1144·4)**
Antigua and Barbuda	1663·0	(1218·4–2193·3)	1·6	(1·2–2·1)
Bahamas	1661·3	(1207·6–2202·7)	6·8	(5·0–9·0)
Barbados	1686·0	(1234·6–2228·6)	5·6	(4·1–7·4)
Belize	1681·2	(1226·3–2228·0)	6·9	(5·0–9·1)
Bermuda	1737·1	(1262·2–2298·9)	1·3	(0·9–1·7)
Cuba	1806·4	(1318·1–2385·5)	233·7	(171·2–308·4)

Location	Age-standardised DALY rates per 100,000	95% uncertainty intervals (age-standardised rates)	Total DALYs in 1,000s	95% uncertainty intervals (total DALYs)
Dominica	1642·3	(1209·7–2164·9)	1·2	(0·9–1·6)
Dominican Republic	1826·8	(1330·7–2409·3)	199·5	(145·2–263·5)
Grenada	1684·1	(1243·2–2224·0)	1·9	(1·4–2·5)
Guyana	2003·8	(1459·0–2629·0)	15·8	(11·4–20·8)
Haiti	1746·3	(1287·1–2284·4)	205·8	(149·7–270·7)
Jamaica	1629·0	(1191·8–2144·9)	48·8	(35·7–64·4)
Puerto Rico	1652·6	(1199·7–2192·3)	64·4	(47·2–84·8)
Saint Kitts and Nevis	1840·1	(1344·4–2450·9)	1·2	(0·9–1·6)
Saint Lucia	1698·2	(1250·8–2246·4)	3·3	(2·4–4·3)
Saint Vincent and the Grenadines	1704·9	(1244·8–2251·1)	2·1	(1·5–2·7)
Suriname	1959·6	(1432·8–2575·4)	11·7	(8·6–15·4)
Trinidad and Tobago	1777·4	(1304·7–2339·2)	26·9	(19·9–35·3)
Virgin Islands	1746·3	(1276·6–2298·2)	2·0	(1·4–2·6)
**Central Latin America**	**1611·1**	**(1187·0–2114·8)**	**4142·4**	**(3051·0–5433·2)**
Colombia	1461·2	(1076·3–1928·2)	733·2	(539·6–966·1)
Costa Rica	1656·4	(1207·0–2185·6)	83·3	(60·7–110·0)
El Salvador	1680·6	(1228·5–2217·0)	106·3	(77·8–140·4)
Guatemala	1706·5	(1249·4–2259·4)	288·1	(208·9–383·1)
Honduras	1590·4	(1152·0–2099·2)	147·1	(106·9–195·9)
Mexico	1653·5	(1210·7–2171·4)	2137·3	(1565·1–2802·7)
Nicaragua	1657·1	(1212·4–2194·3)	106·6	(77·5–141·4)
Panama	1551·7	(1135·2–2038·2)	65·3	(47·8–85·8)
Venezuela	1626·2	(1185·1–2135·5)	475·3	(345·6–624·8)
**Tropical Latin America**	**2115·1**	**(1554·7–2776·4)**	**5056·7**	**(3735·4–6628·2)**
Brazil	2118·4	(1558·3–2783·3)	4916·6	(3635·8–6444·2)
Paraguay	1997·6	(1453·8–2640·9)	140·1	(102·0–184·8)
**North Africa and Middle East**	**1957·6**	**(1445·0–2569·8)**	**12116·5**	**(8906·4–15976·0)**
Afghanistan	2042·9	(1492·7–2692·0)	682·8	(499·8–910·7)
Algeria	1861·0	(1375·3–2462·0)	791·6	(583·9–1048·9)
Bahrain	1980·8	(1449·7–2613·3)	32·9	(24·1–43·4)
Egypt	1814·9	(1349·9–2391·5)	1760·3	(1298·0–2325·9)
Iran	2295·8	(1702·2–3033·6)	2053·9	(1516·2–2714·8)
Iraq	1844·2	(1358·2–2442·2)	767·7	(561·5–1023·4)
Jordan	1897·7	(1395·8–2501·6)	219·6	(159·9–289·7)
Kuwait	1859·7	(1366·4–2471·5)	93·1	(67·9–123·2)
Lebanon	2126·0	(1552·3–2804·7)	111·9	(81·8–147·3)
Libya	1967·2	(1448·8–2597·2)	144·8	(106·1–190·5)
Morocco	2055·8	(1500·8–2701·3)	766·1	(558·4–1010·7)
Oman	1809·5	(1319·1–2395·0)	89·7	(65·1–119·2)
Palestine	2396·9	(1749·9–3172·1)	110·7	(80·3–147·3)
Qatar	1814·9	(1326·3–2387·0)	61·6	(44·7–81·2)
Saudi Arabia	1846·3	(1356·1–2433·4)	743·3	(542·0–984·3)
Sudan	1945·0	(1416·4–2541·7)	742·6	(537·8–983·2)
Syria	1954·4	(1449·8–2579·3)	291·4	(215·4–386·5)
Tunisia	2106·0	(1540·8–2770·6)	258·0	(188·2–340·1)
Turkey	1807·8	(1317·4–2378·2)	1603·1	(1166·4–2106·2)

Location	Age-standardised DALY rates per 100,000	95% uncertainty intervals (age-standardised rates)	Total DALYs in 1,000s	95% uncertainty intervals (total DALYs)
United Arab Emirates	1712·2	(1256·7–2253·0)	192·4	(140·0–253·3)
Yemen	2041·0	(1499·6–2658·8)	586·7	(425·8–769·8)
**South Asia**	**1575·1**	**(1162·0–2043·7)**	**28157·3**	**(20742·5–36673·5)**
Bangladesh	1650·8	(1208·9–2188·3)	2630·5	(1921·6–3487·2)
Bhutan	1579·4	(1167·9–2076·7)	12·0	(8·8–15·8)
India	1561·6	(1155·0–2027·4)	21894·6	(16120·6–28435·8)
Nepal	1773·7	(1309·2–2335·1)	514·4	(378·5–676·6)
Pakistan	1601·0	(1188·7–2105·8)	3105·7	(2281·3–4118·3)
**Southeast Asia, east Asia, and Oceania**	**1267·8**	**(938·2–1659·5)**	**30272·0**	**(22494·6–39466·4)**
**East Asia**	**1248·4**	**(922·5–1628·9)**	**20998·6**	**(15677·0–27272·5)**
China	1248·2	(922·4–1627·4)	20293·8	(15145·6–26345·1)
North Korea	1260·3	(931·9–1658·9)	362·0	(268·1–475·0)
Taiwan (province of China)	1246·9	(913·5–1639·5)	342·7	(252·8–450·3)
**Oceania**	**1386·2**	**(1021·1–1825·8)**	**171·9**	**(126·1–227·5)**
American Samoa	1309·6	(967·1–1736·2)	0·7	(0·5–1·0)
Cook Islands	1429·6	(1047·5–1889·5)	0·3	(0·2–0·4)
Federated States of Micronesia	1360·4	(1003·8–1800·0)	1·4	(1·0–1·8)
Fiji	1359·0	(1003·2–1796·7)	12·4	(9·1–16·4)
Guam	1455·3	(1061·9–1914·0)	2·5	(1·9–3·3)
Kiribati	1372·3	(1015·3–1810·1)	1·5	(1·1–2·1)
Marshall Islands	1350·8	(997·4–1792·2)	0·8	(0·6–1·0)
Nauru	1405·2	(1030·0–1868·8)	0·1	(0·1–0·2)
Niue	1408·1	(1035·7–1875·0)	0·0	(0·0–0·0)
Northern Mariana Islands	1351·4	(989·6–1779·7)	0·6	(0·5–0·8)
Palau	1397·2	(1030·2–1855·6)	0·3	(0·2–0·4)
Papua New Guinea	1389·6	(1021·0–1833·0)	126·9	(93·2–168·1)
Samoa	1344·9	(992·3–1776·5)	2·7	(2·0–3·6)
Solomon Islands	1384·0	(1025·5–1808·8)	8·3	(6·1–10·9)
Tokelau	1409·6	(1036·2–1871·2)	0·0	(0·0–0·0)
Tonga	1327·4	(978·3–1761·2)	1·3	(0·9–1·7)
Tuvalu	1410·5	(1034·4–1871·5)	0·2	(0·1–0·2)
Vanuatu	1374·8	(1015·8–1810·7)	3·8	(2·8–5·0)
**Southeast Asia**	**1293·4**	**(964·4–1697·1)**	**9101·5**	**(6759·1–11965·0)**
Cambodia	1439·5	(1054·5–1897·6)	235·5	(171·5–310·3)
Indonesia	1241·4	(928·7–1631·9)	3384·7	(2534·6–4441·6)
Laos	1401·4	(1030·6–1848·8)	98·2	(71·5–130·6)
Malaysia	1609·2	(1174·7–2136·2)	524·6	(382·6–696·0)
Maldives	1375·6	(1016·7–1804·8)	7·3	(5·3–9·6)
Mauritius	1554·0	(1136·1–2057·3)	22·3	(16·3–29·3)
Myanmar	1219·3	(894·7–1612·4)	679·2	(499·4–899·7)
Philippines	1409·4	(1048·0–1857·0)	1531·7	(1140·2–2023·5)
Seychelles	1317·3	(968·5–1742·1)	1·5	(1·1–1·9)
Sri Lanka	1379·4	(1012·2–1823·9)	318·6	(235·7–421·1)
Thailand	1341·9	(984·5–1760·1)	1074·8	(795·1–1404·8)
Timor-Leste	1322·7	(969·0–1743·6)	16·1	(11·7–21·3)
Vietnam	1150·2	(851·5–1513·1)	1195·1	(881·8–1561·3)

Location	Age-standardised DALY rates per 100,000	95% uncertainty intervals (age-standardised rates)	Total DALYs in 1,000s	95% uncertainty intervals (total DALYs)
**Sub-Saharan Africa**	**1669·3**	**(1226·5–2200·3)**	**15065·9**	**(11006·3–20015·8)**
**Central sub-Saharan Africa**	**1917·0**	**(1388·9–2552·9)**	**2109·0**	**(1524·5–2798·0)**
Angola	1964·8	(1427·4–2613·1)	473·5	(342·2–628·7)
Central African Republic	2039·3	(1483·9–2710·2)	92·0	(66·6–122·1)
Congo	1899·3	(1380·6–2515·5)	90·3	(65·4–120·3)
Democratic Republic of the Congo	1894·1	(1373·6–2522·6)	1396·8	(1005·5–1858·1)
Equatorial Guinea	1968·4	(1425·8–2605·7)	24·3	(17·3–32·4)
Gabon	1934·6	(1407·5–2561·0)	32·0	(23·2–42·3)
**Eastern sub-Saharan Africa**	**1762·1**	**(1292·6–2322·5)**	**5947·7**	**(4329·3–7919·6)**
Burundi	1721·0	(1258·7–2290·4)	165·0	(119·1–218·9)
Comoros	1633·9	(1200·3–2158·6)	11·0	(8·0–14·6)
Djibouti	1649·6	(1206·3–2188·0)	18·2	(13·2–24·2)
Eritrea	1750·0	(1279·3–2309·6)	100·8	(72·9–133·9)
Ethiopia	1706·1	(1261·1–2250·3)	1498·7	(1095·9–1984·8)
Kenya	1709·0	(1260·8–2246·8)	746·6	(546·8–992·7)
Madagascar	1766·1	(1286·0–2315·9)	398·1	(287·0–525·3)
Malawi	1605·5	(1186·8–2118·2)	248·6	(180·7–332·5)
Mozambique	1799·4	(1317·4–2383·3)	415·3	(299·8–552·3)
Rwanda	1800·4	(1318·0–2378·2)	199·3	(144·6–264·6)
Somalia	1775·1	(1289·9–2351·9)	285·6	(206·4–379·3)
South Sudan	1838·3	(1346·5–2417·7)	137·9	(100·1–183·2)
Tanzania	1721·6	(1260·2–2274·5)	799·3	(582·2–1068·6)
Uganda	2153·3	(1564·3–2863·1)	671·9	(484·6–895·7)
Zambia	1629·3	(1187·5–2160·7)	246·7	(177·7–328·3)
**Southern sub-Saharan Africa**	**1598·7**	**(1176·4–2091·7)**	**1230·8**	**(901·6–1616·3)**
Botswana	1604·6	(1174·1–2110·6)	36·8	(26·7–48·7)
Eswatini	1618·7	(1186·4–2129·8)	16·8	(12·2–22·2)
Lesotho	1843·4	(1350·2–2449·7)	36·6	(26·7–48·5)
Namibia	1470·7	(1074·0–1940·5)	32·8	(23·9–43·4)
South Africa	1640·1	(1207·5–2150·4)	926·2	(680·8–1217·9)
Zimbabwe	1385·0	(1020·4–1821·9)	181·6	(132·3–241·7)
**Western sub-Saharan Africa**	**1533·4**	**(1128·6–2021·3)**	**5778·4**	**(4235·8–7696·2)**
Benin	1629·6	(1192·8–2171·9)	164·8	(119·7–220·4)
Burkina Faso	1601·1	(1166·4–2120·9)	290·1	(209·2–386·4)
Cabo Verde	1722·7	(1254·6–2305·3)	9·6	(7·0–12·9)
Cameroon	1649·4	(1199·8–2179·2)	405·3	(291·6–539·2)
Chad	1782·4	(1305·4–2373·3)	215·7	(155·8–289·1)
Côte d'Ivoire	1515·4	(1109·0–2000·5)	339·8	(246·8–452·3)
The Gambia	1854·8	(1349·1–2459·3)	34·6	(25·0–46·0)
Ghana	1584·2	(1163·2–2084·5)	451·7	(327·8–599·6)
Guinea	1616·2	(1171·7–2158·7)	165·6	(120·2–221·3)
Guinea-Bissau	1608·3	(1171·5–2118·7)	25·6	(18·5–34·2)
Liberia	1670·9	(1226·7–2204·4)	70·5	(51·1–93·4)
Mali	1393·6	(1016·3–1836·2)	243·8	(177·3–327·1)
Mauritania	1423·9	(1037·3–1873·8)	49·9	(36·3–66·3)
Niger	1569·2	(1144·2–2069·8)	270·8	(196·6–361·9)

Location	Age-standardised DALY rates per 100,000	95% uncertainty intervals (age-standardised rates)	Total DALYs in 1,000s	95% uncertainty intervals (total DALYs)
Nigeria	1472·7	(1086·1–1941·2)	2609·1	(1898·9–3467·8)
São Tomé and Príncipe	1472·1	(1075·5–1948·5)	2·8	(2·0–3·7)
Senegal	1505·9	(1107·0–1988·4)	196·0	(143·4–260·5)
Sierra Leone	1660·7	(1218·4–2179·4)	118·1	(85·4–156·8)
Togo	1671·2	(1219·8–2221·8)	114·7	(83·7–152·6)

etext 1: Author contributions

Managing the estimation or publications process

Alize J Ferrari, Damian F Santomauro, Charlie Ashbaugh, Simon I Hay, Theo Vos, and Harvey A Whiteford.

Writing the first draft of the manuscript

Alize J Ferrari, Damian F Santomauro, Ana M Mantilla Herrera, Jamileh Shadid, and Harvey A Whiteford.

Primary responsibility for applying analytical methods to produce estimates

Alize J Ferrari, Damian F Santomauro, and Ana M Mantilla Herrera.

Primary responsibility for seeking, cataloguing, extracting, or cleaning data; designing or coding figures and tables

Alize J Ferrari, Damian F Santomauro, Ana M Mantilla Herrera, Jamileh Shadid, Holly E Erskine, and Fiona J Charlson.

Providing data or critical feedback on data sources

Alize J Ferrari, Damian F Santomauro, Ana M Mantilla Herrera, Jamileh Shadid, Holly E Erskine, Fiona J Charlson, Louisa Degenhardt, James G Scott, John J McGrath, Peter Allebeck, Traolach Brugha, Xiaochen Dai, Lalit Dandona, Rakhi Dandona, Florian Fischer, Juanita A Haagsma, Josep Maria Haro, Christian Kieling, Ann Kristin Skrindo Knudsen, G Anil Kumar, Janni Leung, Modhurima Moitra, Ali H Mokdad, Mariam Molokhia, Scott B Patten, Cassandra E I Szoeke, Mohsen Naghavi, Christopher J L Murray, Theo Vos, and Harvey A Whiteford.

Development of methods or computational machinery

Alize J Ferrari, Damian F Santomauro, Xiaochen Dai, Ali H Mokdad, Mohsen Naghavi, Simon I Hay, Christopher J L Murray, Theo Vos, and Harvey A Whiteford.

Providing critical feedback on methods or results

Alize J Ferrari, Damian F Santomauro, Ana M Mantilla Herrera, Jamileh Shadid, Holly E Erskine, Fiona J Charlson, Louisa Degenhardt, James G Scott, John J McGrath, Peter Allebeck, Nicholas J K Breitborde, Traolach Brugha, Xiaochen Dai, Lalit Dandona, Rakhi Dandona, Florian Fischer, Juanita A Haagsma, Josep Maria Haro, Christian Kieling, Ann Kristin Skrindo Knudsen, G Anil Kumar, Azeem Majeed, , Philip B Mitchell, Ali H Mokdad, Mariam Molokhia, Scott B Patten, Michael R Phillips, Joan B Soriano, Murray B Stein, Cassandra E I Szoeke, Mohsen Naghavi, Simon I Hay, Christopher J L Murray, Theo Vos, and Harvey A Whiteford.

Drafting the manuscript or revising it critically for important intellectual content

Alize J Ferrari, Damian F Santomauro, Ana M Mantilla Herrera, Jamileh Shadid, Holly E Erskine, Fiona J Charlson, Louisa Degenhardt, John J McGrath, Peter Allebeck, Corina Benjet, Nicholas J K Breitborde, Traolach Brugha, Florian Fischer, Juanita A Haagsma, Josep Maria Haro, Christian Kieling, Ann Kristin Skrindo Knudsen, Azeem Majeed, Philip B Mitchell, Ali H Mokdad, Mariam Molokhia, George C Patton, Michael R Phillips, Joan B Soriano, Dan J Stein, Cassandra E I Szoeke, Mohsen Naghavi, Simon I Hay, Christopher J L Murray, and Harvey A Whiteford.

Managing the overall research enterprise

Alize J Ferrari, Damian F Santomauro, Louisa Degenhardt, Lalit Dandona, Ali H Mokdad, George C Patton, Mohsen Naghavi, Simon I Hay, Christopher J L Murray, Theo Vos, and Harvey A Whiteford.
